# Non-epitaxial growth of highly oriented transition metal dichalcogenides with density-controlled twin boundaries

**DOI:** 10.1016/j.xinn.2023.100502

**Published:** 2023-08-22

**Authors:** Juntong Zhu, Zhili Hu, Shasha Guo, Ruichun Luo, Maolin Yu, Ang Li, Jingbo Pang, Minmin Xue, Stephen J. Pennycook, Zheng Liu, Zhuhua Zhang, Wu Zhou

**Affiliations:** 1 School of Physical Sciences, CAS Key Laboratory of Vacuum Physics, University of Chinese Academy of Sciences, Beijing 100049, China; 2 State Key Laboratory of Mechanics and Control for Aerospace Structures, Key Laboratory for Intelligent Nano Materials and Devices of Ministry of Education, and Institute for Frontier Science, Nanjing University of Aeronautics and Astronautics, Nanjing 210013, China; 3 School of Materials Science and Engineering, Nanyang Technological University, Singapore 639798, Singapore; 4 Environmental Chemistry and Materials Centre, Nanyang Environment and Water Research Institute, Nanyang Technological University, Singapore 637141, Singapore

## Abstract

Twin boundaries (TBs) in transition metal dichalcogenides (TMDs) constitute distinctive one-dimensional electronic systems, exhibiting intriguing physical and chemical properties that have garnered significant attention in the fields of quantum physics and electrocatalysis. However, the controlled manipulation of TBs in terms of density and specific atomic configurations remains a formidable challenge. In this study, we present a non-epitaxial growth approach that enables the controlled and large-scale fabrication of homogeneous catalytically active TBs in monolayer TMDs on arbitrary substrates. Notably, the density achieved using this strategy is six times higher than that observed in convention chemical vapor deposition (CVD)-grown samples. Through rigorous experimental analysis and multigrain Wulff construction simulations, we elucidate the role of regulating the metal source diffusion process, which serves as the key factor for inducing the self-oriented growth of TMD grains and the formation of unified TBs. Furthermore, we demonstrate that this novel growth mode can be readily incorporated into the conventional CVD growth method by making a simple modification of the growth temperature profile, thereby offering a universal approach for engineering of grain boundaries in two-dimensional materials.

## Public summary

• Differences in diffusivity of metal sources trigger the non-epitaxial growth of twin boundaries.
• The growth mechanism of high-density 1D twin boundaries on arbitrary substrates is revealed.
• A possible universal strategy for grain boundary engineering in 2D materials is proposed.
• Density-controllable twin boundaries provide a promising platform for exploring novel quantum states in 1D electronic systems.

## Graphical abstract

**Figure:**
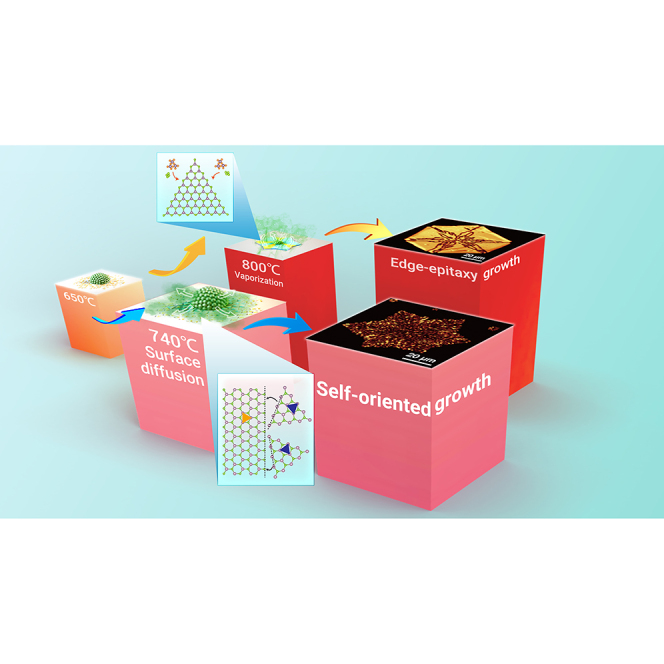

## Introduction

Transition metal dichalcogenides (TMDs) have garnered significant attention due to their fascinating physicochemical properties and emerging applications.^1^^,^^2^^,^^3^ Defect engineering, involving the introduction of specific point defects (eg, vacancies^4^ or dopants^5^) or one-dimensional defects (e.g., grain boundaries [GBs], domain boundaries,^6^^,^^7^ or edges^8^) into the two-dimensional (2D) TMD crystal lattices, plays a crucial role in enhancing and diversifying the intrinsic properties of 2D materials. For instance, catalytic activity for hydrogen evolution can be enhanced by introducing strained sulfur vacancies into the MoS_2_ basal planes,^9^ and the electrical transport properties of MoS_2_ can be tuned by GBs with varying misorientation angles.^10^ Owing to the diverse atomic configurations adopted by GBs in TMDs, contingent upon the misorientation angle, GB engineering emerges as an exceptionally versatile means to manipulate material properties.^11^ Compared to small-angle GBs with sparsely separated 5|7 dislocation cores (i.e., pentagon-heptagon pairs) or 4|6 membered rings (i.e., tetragon-hexagon pairs),^4^ 60° GBs in TMDs, commonly referred to as twin boundaries (TBs), exhibit a distinct atomic configuration, primarily comprising strings of four-membered rings separated by individual eight-membered rings.^4^^,^^12^^,^^13^^,^^14^ These TBs serve as remarkable one-dimensional electron systems, showcasing notable features such as quantum well states,^15^ charge density wave ordering,^16^ and Tomonaga-Luttinger liquid behavior.^17^ Furthermore, the incorporation of eight-membered rings into the TBs, forming 4|8 membered pairs, significantly enhances the catalytic activity for the hydrogen evolution reaction (HER).^6^^,^^18^ Consequently, the controlled and large-scale fabrication of TBs, featuring designated atomic structure and controlled density, in monolayer TMDs holds crucial importance for fully exploring their potential in electronic and electrocatalytic applications.

Numerous approaches have been developed to construct and control TBs in TMDs.^19^^,^^20^ For instance, TBs can be generated by inducing Se deficiency through thermal annealing^19^ or incorporating excess Mo atoms into the lattice during molecular beam epitaxial (MBE) growth on lattice-matched substrates.^20^ Both approaches result in the creation of high-density inversion domains in MoSe_2_ surrounded by TBs. However, the size of such inversion domains is typically limited to a few nanometers, leading to the formation of short segments of TBs. This limitation in size may cause experimentally measured properties to deviate from the intrinsic properties of TBs.^21^ Moreover, the TB segments originated from structural inversion in MBE-grown samples consist exclusively of four-membered rings. On the other hand, the chemical vapor deposition (CVD) growth method, utilizing van der Waals epitaxy on single-crystalline sapphire or graphene substrates,^22^ can produce much longer TBs, typically on the order of micrometers, through the coalescence of 60° rotated grains. These TBs often contain a higher proportion of eight-membered rings,^12^ a structural feature that benefits catalytic applications. However, the density of TBs in such CVD-grown samples is constrained by the low density of nucleation sites, resulting in poor tunability.

Herein, we propose a novel non-epitaxial growth approach to produce uniform TBs in TMDs with adjustable density on arbitrary substrates, including amorphous SiO_2_, glass, single-crystalline sapphire, and mica. During the nucleation phase, a high density of small precursor particles diffuses and re-deposits surrounding larger precursor mounds at low temperatures, acting as nucleation sites. In the subsequent growth stage, the significantly different diffusivities of metal feedstocks on the bare substrate and the as-formed TMD surface limit the growth and size of the satellite TMD grains evolved from the surrounding tiny particles. This phenomenon facilitates the self-orientation of these grains as they merge with the larger central grains evolved from the precursor mounds. By adjusting the growth temperature, we can effectively control the densities of nuclei and TBs, enabling large-scale TB-density engineering. This growth mechanism is well supported by experimental analysis of intermediate products and multigrain Wulff construction simulations with a newly developed phenomenological method. Furthermore, we demonstrate that the self-oriented growth can be achieved by incorporating a precipitation phase into the temperature profile in the conventional CVD method. The resulting TBs in monolayer MoSe_2_ obtained from this new growth strategy contain highly reactive eight-membered rings, leading to substantially improved HER performance.

## Results and discussion

### Synthesis and characterization of TB-rich MoSe_2_

**Figure 1:**
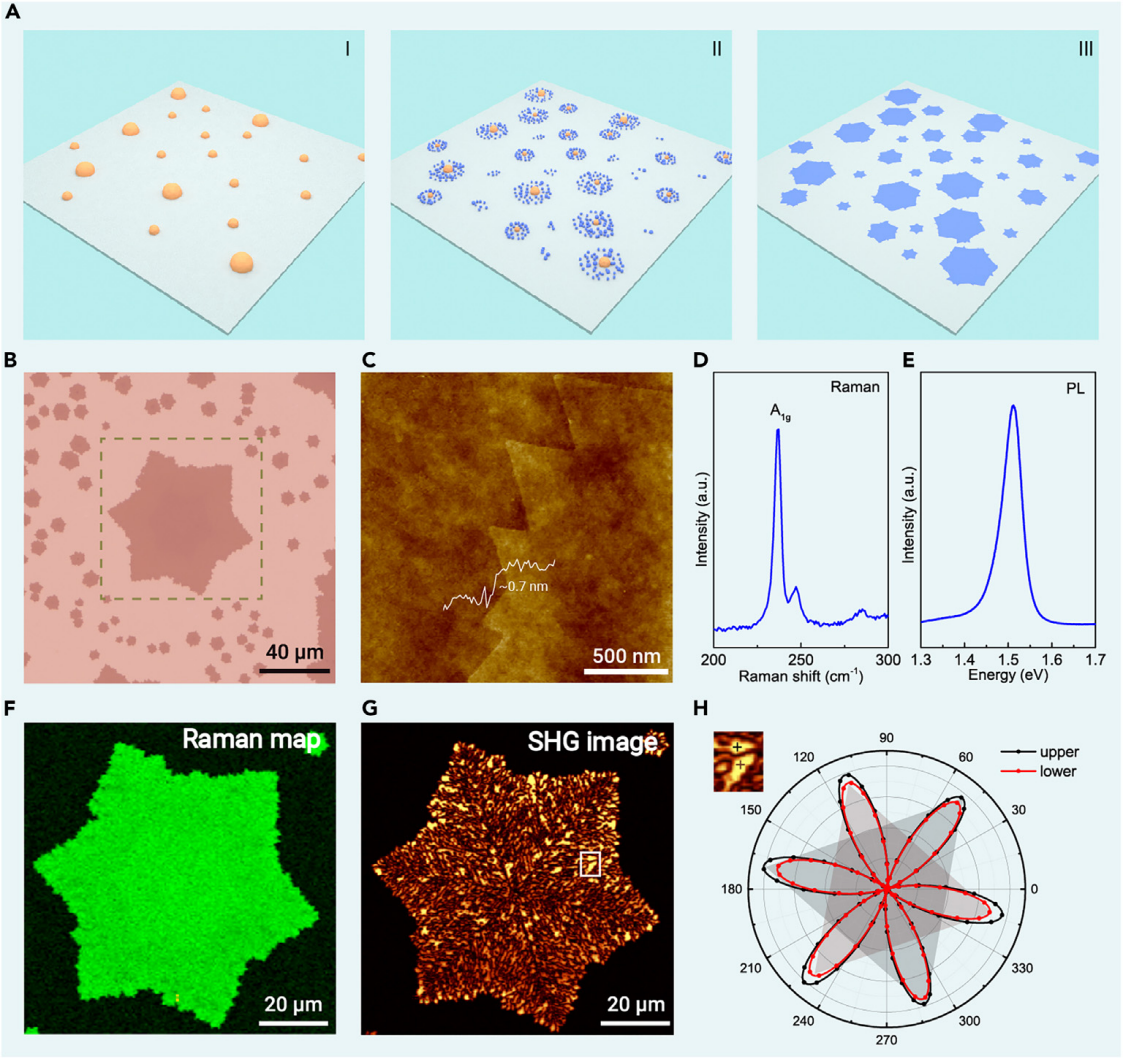
Growth and characterization of MoSe_2_ with a high density of TBs (A) Schematic of the growth process. (i) Mo precursor (yellow mounds) is spin coated onto the substrate; (ii) small Mo-containing particles (blue spheres) diffuse out from the central precursor mounds at 660°C; (iii) fuzzy hexagonal or Star-of-David-shaped MoSe_2_ flakes (blue flakes) grow at 740°C. (B) Optical image of a MoSe_2_ flake grown on the SiO_2_/Si substrate. (C) AFM image of the as-grown MoSe_2_ flake with jagged edges. (D and E) Raman (D) and PL (E) spectra of the as-grown MoSe_2_ monolayer. (F and G) Raman intensity mapping (F) and polarized SHG mapping (G) of the fuzzy Star-of-David-shaped MoSe_2_ flake highlighted in (B). The Raman intensity mapping of the A_1g_ peak exhibits uniform contrast, while the SHG mapping contains a high density of low-contrast lines. (H) Polarization-resolved SHG spectra of adjacent domains as marked by the white frame in (G). The direction of each intensity maximum points to the armchair direction.

Figure 1A illustrates the growth process schematically (see section “material and methods” and Figure S1 for details). Initially, the Mo precursor dissolved in KOH solution is spin coated onto the SiO_2_/Si substrate, leading to the formation of discrete and sparsely distributed mounds as the solution dries (Figure S2). When the growth temperature reaches 660°C, the Mo precursors diffuse into numerous Mo-containing particles, serving as nucleation sites for the growth of MoSe_2_ (Figure S3), which contrasts the vaporization and redeposition of metal sources in conventional CVD methods.^23^ Finally, MoSe_2_ flakes grow from the central mounds and the surrounding particles, seamlessly fusing into larger MoSe_2_ flakes with fuzzy hexagonal or Star-of-David shapes at 740°C (Figures 1B and S4). Atomic force microscopy (AFM) measurements (Figure 1C) confirm that the resulting flakes are monolayers. Additionally, the characteristic Raman peak of the A_1g_ mode is observed at 238 cm^−1^ (Figure 1D), and the photoluminescence (PL) peak of the A exciton is found at 1.54 eV (Figure 1E), both verifying the formation of monolayer crystalline MoSe_2_. Raman mapping of the A_1g_ mode (Figure 1F) shows homogeneous spectroscopic quality in the as-formed MoSe_2_ monolayer. Notably, unlike typical exfoliated single crystals (Figure S5B), the as-grown MoSe_2_ monolayers exhibit jagged edges and an extra defect-related Raman peak at 247 cm^−1^ (Figure 1D).^24^

We utilized polarized second harmonic generation (SHG) mapping to investigate the presence of GBs in the as-grown MoSe_2_ at the macroscopic scale. As depicted in Figure 1G, the pervasive low-contrast lines observed within the monolayer MoSe_2_ flakes indicate a high density of GBs,^25^ which can account for the sharp defect-related Raman peak observed in Figure 1D. To identify the tilt angle of these GBs, we collected polarization-resolved SHG spectra from adjacent grains, where the directions of the intensity maxima of the 6-fold anisotropic polarization pattern point to the armchair directions of the MoSe_2_ lattice. As demonstrated in Figure 1H, the polarization patterns from the two adjacent grains exhibit the same armchair directions, possibly with a relative rotation of 60°. This, in conjunction with the clear presence of a GB between the two grains (inset of Figure 1H), implies that the two grains are misoriented by 60° and separated by a TB. Moreover, the polarization-resolved SHG images display similar levels of intensity inside each MoSe_2_ flake (Figure S6), in contrast to the substantial intensity difference observed between flakes with non-60° relative rotations, suggesting that the GBs (the low-contrast lines in the SHG images) within individual MoSe_2_ flakes are uniformly TBs.

### Atomic structural analysis

**Figure 2:**
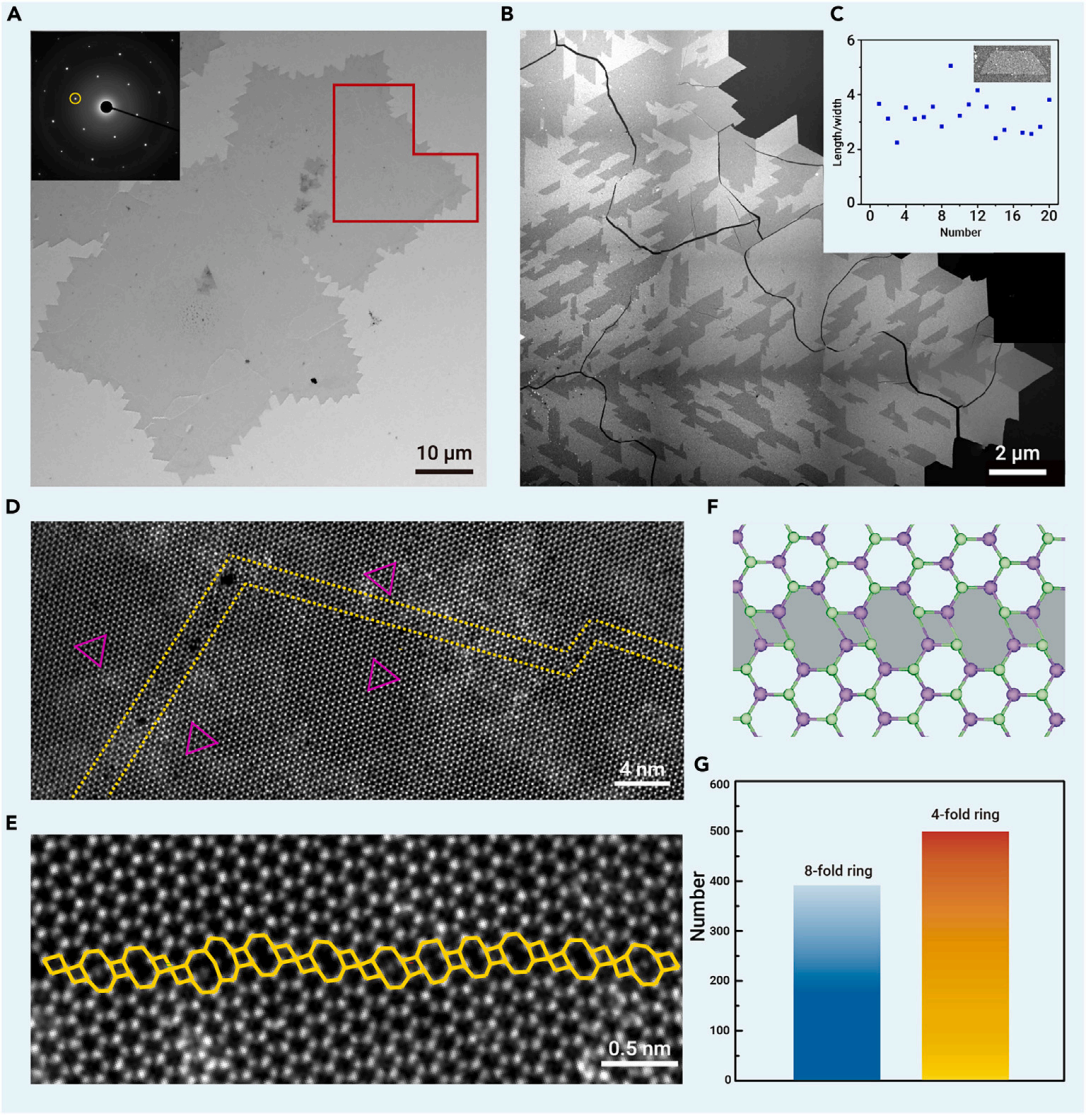
Distribution and atomic structure of the high-density TBs in MoSe_2_ (A) Low-magnification BF-TEM image of MoSe_2_ flakes. Inset: SAED pattern acquired from the upper-right corner of the flake. (B) DF-TEM image of the selected region highlighted by the red lines in (A). The image was stitched by multiple DF-TEM images at higher magnification. The black lines inside the MoSe_2_ flake are cracks formed during the sample transfer. (C) Statistics of the aspect ratio of the twin domains. Inset shows the DF-TEM image of a trapezoid twin domain. (D) HAADF-STEM image of a segmented TB. The violet triangles illustrate the inversed lattice orientations on both sides of the TB, and the yellow dashed lines depict the TB region. (E) High-resolution HAADF-STEM image of a TB in MoSe_2_. Alternating four- and eight-membered rings are marked by yellow tetragons and octagons. (F) The top view of a TB model with consecutive four- and eight-membered rings. The green and purple balls represent the Se and Mo atoms, respectively. (G) Statistics for eight- and four-membered rings in TBs from analysis of 10 different sample regions.

Transmission electron microscopy (TEM) was employed to analyze the distribution of TBs in the monolayer MoSe_2_ flakes. Figure 2A presents a TEM bright-field (BF) image of the flakes with jagged edges. The corresponding selected area electron diffraction (SAED) pattern shows only one set of diffraction spots, indicating the absence of non-60° GBs. To reveal the distribution of twin crystal domains, dark-field (DF) TEM imaging was performed by selecting one of the first-order diffraction spots to form the diffraction-contrast image. As shown in Figure 2B, distinct domains with alternating contrast are clearly visible across the entire MoSe_2_ flake, arising from the 3-fold symmetry of the MoSe_2_ lattice and the 60° rotation between adjacent twin domains. Typically, the twin domains show a trapezoidal shape with a length-to-width ratio of approximately 3 (Figure 2C), which differs from the equilateral triangular MoSe_2_ inversion domains induced by Se deficiency.^19^ The density of TB is estimated to be 5.1 μm/μm^2^ (see details in Note S1 and Figure S7), which is 25 times higher than that observed in typical Star-of-David-shaped MoS_2_ flakes (0.2 μm/μm^2^) grown by the CVD method on SiO_2_/Si substrates^12^ and six times higher than that in the highly oriented MoS_2_ film (0.8 μm/μm^2^) grown on sapphire.^22^

The atomic configuration of the TBs was elucidated by aberration-corrected scanning transmission electron microscopy (STEM) high-angle annular dark-field (HAADF) imaging. As depicted by the yellow dashed lines in Figure 2D, a TB comprising multiple segments is highlighted, while the crystallographic orientations of the two adjacent domains are denoted by the violet triangles. The magnified HAADF-STEM image (Figure 2E) provides a direct visualization of the atomic arrangements along the TB, characterized by a distinctive alternation of four- and eight-membered rings, exhibiting a molybdenum-rich structure (a structural model is shown in Figure 2F). The relative populations of eight- and four-membered rings within the TBs are summarized in Figure 2G, based on an analysis performed across 10 randomly selected regions (Figure S8). The proportion of eight-membered rings within our sample is approximately 40%, a figure that significantly exceeds those previously reported within literature.^4^^,^^12^ The incorporation of a substantial proportion of reactive eight-membered rings within the TBs is anticipated to introduce a dramatic enhancement in the electrocatalytic activity of the as-grown TB-rich monolayer MoSe_2_. Further discussion on this aspect will be provided in the subsequent sections.

### Non-epitaxial growth mechanism

To investigate the mechanism underlying the formation of high-density TBs in MoSe_2_, we conducted a thorough examination of intermediate products at various growth temperatures. This was accomplished in conjunction with the execution of phenomenological simulations and density functional theory (DFT) calculations. Given the comprehensive information gathered, we propose a self-oriented nucleation and growth mechanism as the genesis of high-density TBs in monolayer TMDs.

**Figure 3:**
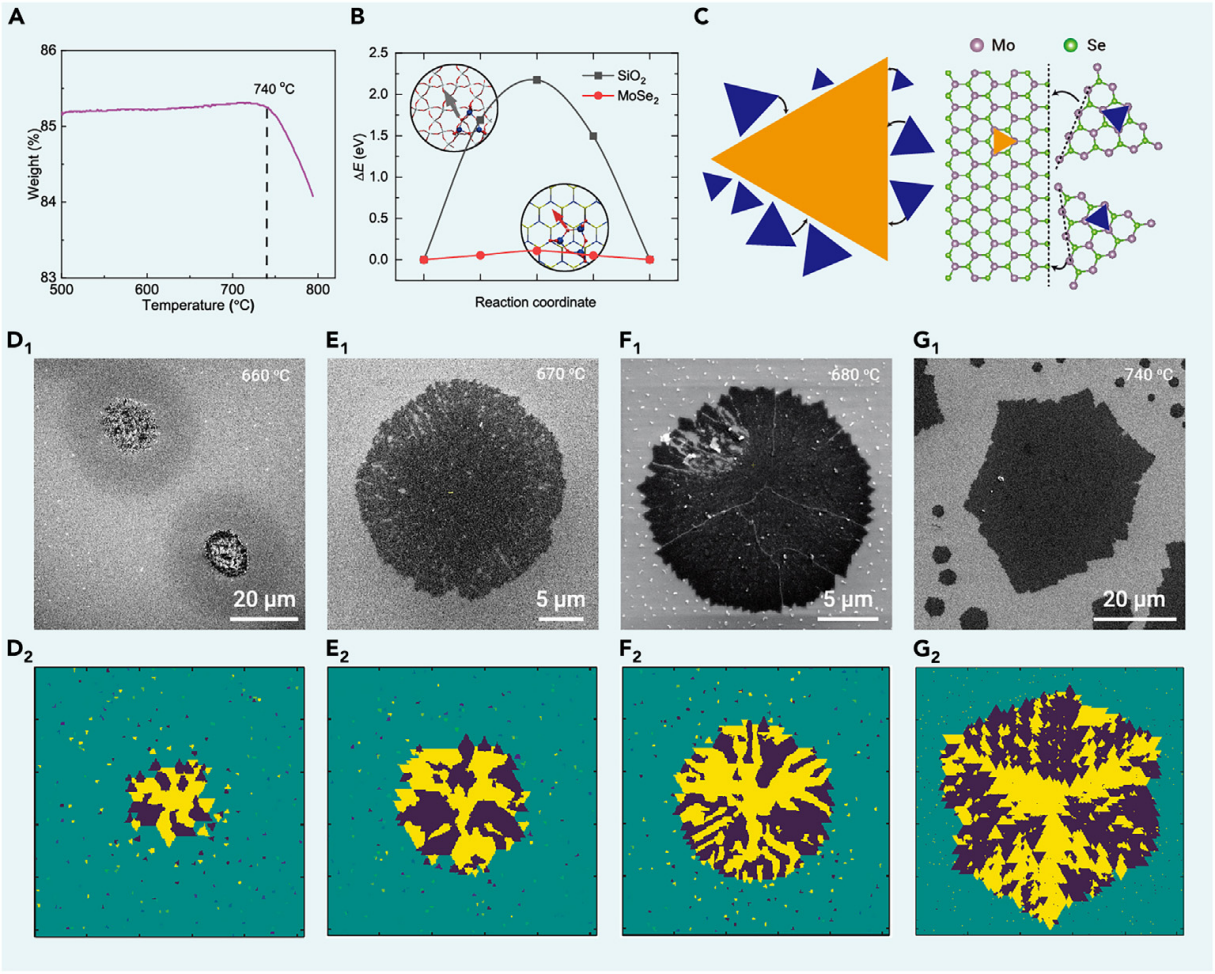
Self-oriented growth of MoSe_2_ with high-density TBs (A) TGA measurement of the Mo precursor. The weight is normalized to the total weight of the Mo precursors containing (NH_4_)_6_Mo_7_O_24_·4H_2_O and KOH at room temperature (Figure S9). The TGA result indicates that the state of the precursor and the growth mode of MoSe_2_ should differ dramatically below and above 740°C. (B) The calculated diffusion energy barriers of Mo_3_O_9_ on the surfaces of SiO_2_ and MoSe_2_, respectively. The diffusion barrier of Mo_3_O_9_ on the surface of SiO_2_ is much higher than that on the surface of MoSe_2_. (C) Schematic of the formation of TBs via self-oriented nucleation and growth. (D_1_–G_1_) SEM images of samples grown at 660°C, 670°C, 680°C, and 740°C, respectively. The morphology of the products transforms from a large mound to an incomplete rim and finally to a fuzzy Star-of-David-shaped flake. (D_2_–G_2_) Phenomenological simulations of MoSe_2_ samples in D_1_–G_1_. The fractal landlocked distribution and length/height ratio of trapezoidal twinned domains in G_2_ are similar to the experimental results revealed by DF-TEM imaging.

Thermogravimetric analysis (TGA) (Figure 3A) demonstrates that the sublimation or decomposition of the Mo precursor is negligible within the temperature range of 500°C and 740°C. Within the temperature bracket of 660°C and 740°C, Mo precursors, reportedly in the form of Mo_3_O_9_ molecules, predominantly diffuse from the precursor mounds as surface adsorbates,^26^^,^^27^ functioning as the primary source of Mo feedstock. Notably, the presence of circular clouds composed of minuscule particles surrounding the large Mo precursor mounds is observed in the intermediate sample obtained at 660°C (Figures 3D_1_, S3, and S10). This observation appears to challenge the established concept of Ostwald ripening, whereby larger particles tend to evolve through consumption of their smaller counterparts. This discrepancy suggests that the diffused Mo_3_O_9_ molecules have interacted with the surrounding Se to form MoO_x_Se_y_ alloys (Figure S10). These alloys, possessing higher melting points, are inclined to deposit more readily on the substrate. Conversely, when the identical precursor was annealed at 660°C without an Se atmosphere, such minuscule particle clouds were absent (Figure S11).

Following the formation of particle clouds, both the central Mo precursor mounds and the minuscule particles in their vicinity function as nucleation sites for the subsequent growth of MoSe_2_ grains. Apart from the local feedstock present at these nucleation sites, the Mo precursor mounds also act as the principal suppliers of Mo feedstock through surface diffusion, given the negligible source of vapor-phase metal at lower temperatures. DFT calculations reveal that the primary form of Mo feedstock (i.e., Mo_3_O_9_) diffuses 10 orders of magnitude faster on the newly formed MoSe_2_ surfaces than on the bare SiO_2_ substrate (Figure 3B; Table S1). Owing to this significant discrepancy in diffusion rates, isolated MoSe_2_ grains distant from the Mo precursor mounds struggle to secure a sufficient supply of Mo feedstock, resulting in their slow growth. Consequently, a sizable central MoSe_2_ “continent” emerges from each Mo precursor mound, while only small MoSe_2_ “islands” evolve from the surrounding tiny MoO_x_Se_y_ particles, adopting random orientations due to the absence of van der Waals epitaxy. As the growth proceeds, the central MoSe_2_ continent expands, gradually incorporating the surrounding MoSe_2_ islands. Importantly, upon contacting with the large MoSe_2_ continents, the small MoSe_2_ islands undergo reorientation to align with the lattice of the large domains. This alignment is achieved via capillary forces,^28^ generating a seamless multigrain flake (Figure 3C) that exhibits either 0° or 60° misorientation crossing all GBs. We refer to this non-epitaxial growth model as the self-oriented growth mode.

Within the self-oriented growth mode, the disparity in diffusivity of Mo_3_O_9_ on SiO_2_ and MoSe_2_ surfaces, as well as the growth rate of MoSe_2_, significantly influences the morphological evolution of the growth. As temperature escalates, the growth rate surges at a rate much more pronounced than the diffusion rate of feedstocks (see the details in Note S2). Therefore, we primarily examine the impact of temperature on the growth rate in the subsequent discussion. At low temperatures (≤670°C), the expansion of the central MoSe_2_ continent is comparatively sluggish, permitting a small amount of Mo feedstock to reach offshore MoSe_2_ nucleation sites through surface diffusion. This results in the formation of a cloud composed of either small MoO_x_Se_y_ particles or diminutive MoSe_2_ grains (Figure 3D_1_). Due to the isotropic diffusion of Mo feedstock on SiO_2_, this cloud is circular and ultimately evolves into a loosely packed rounded rim of MoSe_2_ as the growth continues (Figures 3E_1_ and S12). Upon reaching a temperature of 680°C, the MoSe_2_ continent consumes the Mo feedstock at an accelerated rate, causing even slower growth of the cloud, which consequently results in a densely packed rounded MoSe_2_ rim (Figures 3F_1_ and S13). At elevated temperatures of 710°C and 740°C, the MoSe_2_ continent nearly depletes all available Mo feedstock, culminating in a fuzzy Star-of-David shape (see details in Note S3; Figures 3G_1_ and S14).

Our theory regarding the self-oriented nucleation and growth of multiple-twinned crystals is further supported by phenomenological simulations utilizing a custom multigrain Wulff construction algorithm (see section “material and methods” for details). In this context, we employ the kinetic constant *k* of growth fronts of MoSe_2_ grains as a descriptor to represent the influence of the growth temperature via an Arrhenius equation (see details in Note S2),^29^ with a higher *k* corresponding to a higher temperature. As depicted in Figures 3D_2_–3G_2_, an increase in *k* triggers a morphological evolution in the sample. It transforms from a central MoSe_2_ continent accompanied by a circular cloud of diminutive surrounding MoSe_2_ grains (Figure 3D_2_) to a loosely packed rounded MoSe_2_ rim (Figure 3E_2_), and subsequently to a compact rounded MoSe_2_ rim (Figure 3F_2_). In an extreme scenario, where the difference between mobilities of feedstock on the substrate surfaces and MoSe_2_ is virtually infinite and the Mo feedstock is in excess, the large-scale simulation (Figure 3G_2_) reproduces almost identically the morphology and distribution of twin crystals observed experimentally in Figure 2B. This outcome is in line with our previous predictions.

### TB engineering and HER performance

**Figure 4:**
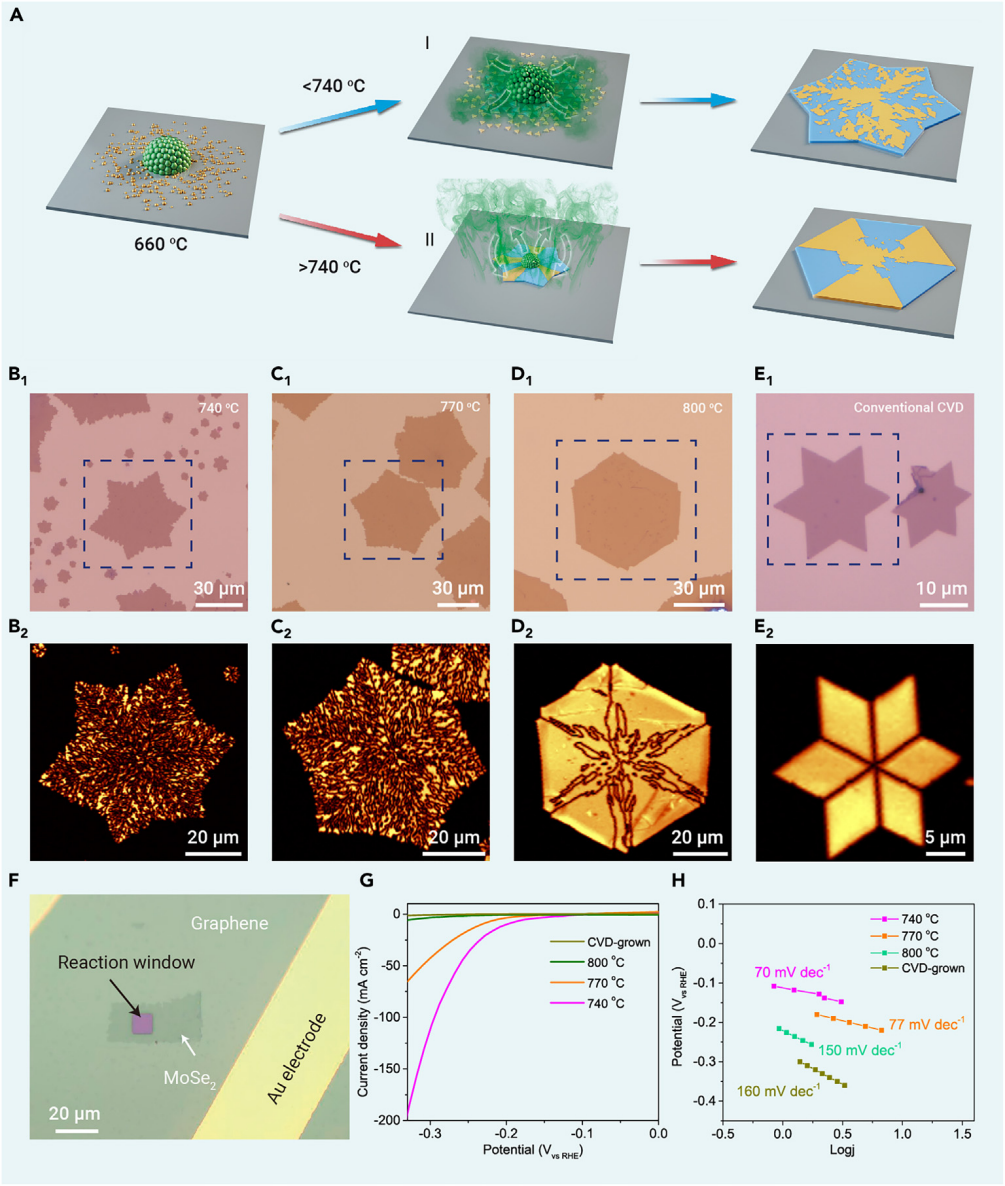
TB-density engineering in MoSe_2_ monolayers and HER performance (A) Schematic of the self-oriented growth and the edge-epitaxial growth modes at different temperatures. (i) Below 740°C, where the gas-phase feedstock is negligible, the diffusion and redeposition of Mo precursors from the central mounds onto the substrate surface produces many MoSe_2_ islands, and the expansion of the central MoSe_2_ “continent” is accompanied by the annexation of surrounding islands under self-oriented growth. In contrast, (ii) above 740°C, the gas-phase feedstock dominates, resulting in fewer islands on the substrate, and the expansion of the continent is dominated by epitaxial growth along the edges. The green arrows represent the migration of Mo sources. (B–D) Optical (B_1_–D_1_) and SHG (B_2_–D_2_) images of monolayer MoSe_2_ grown at 740°C, 770°C, and 800°C for 3 min, respectively, using the OH-assisted CVD method. (E) Optical (E_1_) and SHG (E_2_) images of MoSe_2_ grown at 740°C by a conventional molten-salt-assisted CVD method without precipitation stage. The as-grown MoSe_2_ flake contains only six TBs. (F) Optical micrograph of a MoSe_2_ micro-device. The micro-device includes a PMMA reaction window on the MoSe_2_ basal plane and graphene and Au electrodes. (G and H) Polarization curves of the current density (G) and the corresponding Tafel plots (H) of the micro-devices for the basal planes of 740°C, 770°C, and 800°C-grown and CVD-grown MoSe_2_, respectively.

The aforementioned discussion implies that, at low growth temperatures, the diffusion and redeposition of metal source play a critical role in facilitating the self-oriented nucleation and growth of TMDs with a high density of TBs (Figure 4Ai). Conversely, when the growth temperature exceeds 740°C, the sublimation of the Mo precursor becomes substantial (Figure 3A), and the formation of surrounding MoO_x_Se_y_ particles via diffusion and deposition is significantly reduced. This results in a marked decrease in the number of domains and GBs within each MoSe_2_ flake (Figure 4Aii). Correspondingly, the high-temperature growth of MoSe_2_ flakes is predominantly guided by edge-epitaxial growth using a vapor-phase metal source (Figure S15). This sharply contrasts with the self-oriented growth observed at lower temperatures, which operates via surface diffusion and the annexation of surrounding smaller islands.

The temperature-dependent competition between these two distinct growth mechanisms suggests that the density of TBs can be engineered simply through the careful modulation of the growth temperature. The SHG images in Figures 4B_2_–D_2_ indeed manifest the decrease of TB density in MoSe_2_ samples as the growth temperature rises from 740°C to 770°C and finally 800°C. In addition, the transition from the self-oriented growth to edge-epitaxial growth, concurrent with rising temperature, likely reduces the availability of Mo feedstock due to the rapid depletion of the Mo mounds. Consequently, the monolayer flake edges evolve from Mo-terminating zigzag edges to Se-terminating edges, accompanying an increase in Se chemical potential (see the details in Note S4 and Figure S16). This transformation reshapes the flakes from a Star-of-David shape (740°C; Figure 4B_1_) into a hexagon (800°C; Figure 4D_1_). Notably, the edge regions of these samples exhibit lower TB densities, primarily due to the surrounding grains, which serve as nuclei for the subsequent growth of MoSe_2_, are denser near the central mound, and become sparser as they move away from the central mound (Figure S3). This variation in nucleus distribution influences the TB density in the edge regions. Additionally, the edge epitaxy assumes a dominant role during the high-temperature growth (>740°C), contributing further to the lower grain density in the edge region. Consequently, the TBs in the edge regions of hexagonal-shaped MoSe_2_ flakes are particularly scarce (Figure 4D_1_). Remarkably, this self-oriented nucleation and growth strategy exhibits insensitivity toward the growth substrate (Table S1). We demonstrate that MoSe_2_, with high-density TBs, can also be successfully grown on amorphous glass and single-crystalline sapphire and mica (Figure S17). Furthermore, this approach can be extended to the growth of other TMD materials, such as WSe_2_ (Figure S18), thus serving as a universal methodology for TB engineering in 2D TMD materials.

Given the high proportion of the catalytically active eight-membered rings in our TBs (Figure 2G), we have adopted a single-entity on-chip micro-electrochemical cell (Figure S19) to assess the HER performance of the above-mentioned monolayer MoSe_2_ samples with varying TB densities. For a systematic comparison, we examined monolayer MoSe_2_ samples grown under 740°C, 770°C, and 800°C, displaying an averaged TB density of 5.1, 3.2, and 1 μm/μm^2^, respectively. These results were compared with a MoSe_2_ monolayer sample grown using the conventional CVD method, which exhibited negligible TBs (∼0.2 μm/μm^2^ in density). To exclude the potential impacts from the Au electrode^30^ and edges of MoSe_2_,^18^ the as-prepared materials were first transferred onto HER-inert graphene electrodes, and then reaction windows were precisely defined on the basal plane of the MoSe_2_ devices, while the rest of the sample area was protected by poly(methylmethacrylate) (PMMA) films (∼1 μm thick) (Figure 4F). Interestingly, we discover that the presence of TBs with eight-membered rings can remarkably boost the HER activity of MoSe_2_, as shown by the escalating HER current density in line with increasing TB density in these samples. Significantly, the 740°C-grown MoSe_2_, possessing ultra-high-density TBs, demonstrated an approximately 100-times increase in current density at −300 mV vs. reversible hydrogen electrode (RHE) when compared to that in the conventional CVD-grown MoSe_2_. The potential influence of Se vacancies^9^^,^^31^ on the observed catalytic trend can be excluded by the similar Se vacancy concentrations across the samples (Figure S20). We also conducted electrochemical stability tests of 740°C-grown MoSe_2_. Figure S21 demonstrates that the TB-rich MoSe_2_ samples exhibit decent stability, maintaining their performance over a period of 15 h at a current density of 10 mA cm^−2^. Together, these results suggest that the key catalytic contributors are TBs with eight-membered rings, while the contributions from Se vacancies and the pristine MoSe_2_ basal plane are negligible. This observation is fully consistent with previous *ab initio* calculations.^13^^,^^32^ It is worth noting that the monolayer MoSe_2_ prepared at 740°C showed the best HER activity with an overpotential of −195 mV at a current density of 10 A cm^−2^ (Figure 4G) and a Tafel slope of 70 mV dec^−1^ (Figure 4H), which are superior to previously reported GB-rich monolayer TMD materials (Table S2).

### Extending non-epitaxial TB growth to conventional CVD methods

**Figure 5:**
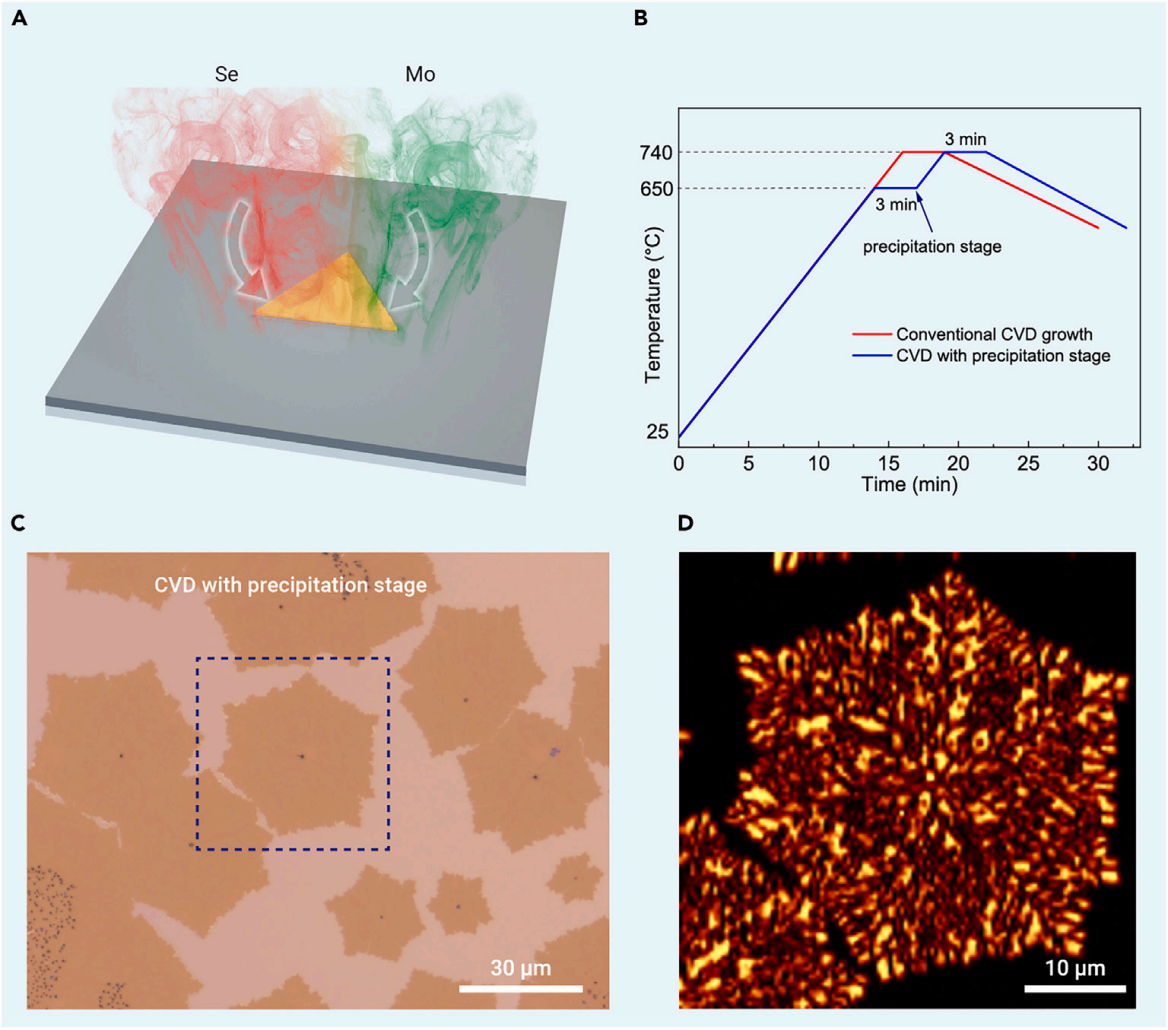
Growth of high-density TBs by a modified CVD method (A) Schematic of conventional CVD growth method using vaporized metal source. (B) Temperature profiles for a conventional CVD growth process (red) and a modified CVD process with a precipitation stage at 650°C (blue). (C and D) Optical (C) and SHG (D) images of MoSe_2_ grown at 740°C using the modified CVD method with a precipitation stage, showing a high density of TBs.

In conventional CVD growth methods that use solid-state metal sources (Figure 5A), such as metal powders,^33^ metal oxides,^34^ and pure TMD powders,^35^ the metal precursor powders typically undergo a vaporization-redeposition process. Therefore, if we add a precipitation stage to the temperature profile prior to TMD growth, the vaporized metal source could pre-deposit onto the substrate, generating large feedstock mounds similar to those observed in our OH-assisted CVD method discussed above and introducing high density of TBs through the self-oriented growth mode. We test this hypothesis using molten-salt-assisted CVD growth.^23^ A precipitation stage at 650°C was added to the temperature profile, which is below the MoSe_2_ growth temperature (Figure 5B; see more experimental details in section “material and methods”). This stage enables the deposition of large MoO_x_ particles onto the substrate surface (Figure S22). During the subsequent temperature ramp from 660°C to 740°C, the metal feedstock diffusing out from the large MoO_x_ particles generates a high density of nucleation sites, which then evolve into small MoSe_2_ domains and merge with the central continents following the self-oriented nucleation and growth (Figure 4Ai). This modified CVD route produces MoSe_2_ monolayers with high-density TBs (Figures 5C and 5D), providing strong evidence for our theory. In contrast, the molten-salt-assisted CVD growth, when performed without the precipitation stage, mainly produces Star-of-David MoSe_2_ with only six TBs (Figures 4E_1_ and S23).

Based on the results presented, we propose that surface diffusion and redeposition of metal precursors cannot be completely eliminated in CVD growth. As such, the self-oriented nucleation and growth mechanism should come into play to different extents in all CVD growth. The observed evolution of TB density and morphology of TMD at different temperatures (Figures 4B_2_–4D_2_) offers guidance for growing monolayer TMDs for specific applications. For instance, for electronic device applications where high-quality single crystals are preferred, conditions of low growth temperature and high metal flux conditions may not be ideal due to the dominance of the self-oriented nucleation and growth mechanism. On the contrary, elevated growth temperatures that inhibit the pre-deposition of metal precursors facilitates the edge-epitaxial growth of high-quality single crystals, which can also explain the use of ultra-high temperatures (over 950°C) for wafer-scale TMD single-crystal growth.^36^^,^^37^^,^^38^

## Conclusion

We have unveiled and demonstrated a non-epitaxial growth strategy that introduces highly reactive TBs in MoSe_2_ and WSe_2_ monolayers through a self-oriented growth mechanism. The synthesized TB-rich MoSe_2_ monolayers featuring plentiful eight-membered rings indeed exhibit impressive electrocatalytic performance. The mechanism is built on the surface diffusion and redeposition of metal sources at moderate temperatures, as well as the significant difference in the diffusivities of metal feedstocks on the surfaces of substrate and the newly formed TMD. We demonstrate that both self-oriented growth and the conventional edge-epitaxial growth modes are prevalent in various CVD-based methods. Therefore, the density and atomic structure of TBs can be engineered by modulating the competition between these two growth modes through adjusting the growth temperature. Overall, our study paves the way to large-scale GB engineering of TMD monolayers via CVD growth and provides a promising platform to explore novel quantum states in 1D electronic systems and their potential applications in electronics and electrocatalysis.

## Material and methods

Please refer to the supplemental information for details on methods.

## Lead contact website

http://zhouwu.ucas.ac.cn

## Supplemental information

Document S1. Figures S1–S23, Tables S1 and S2, and Notes S1–S4

Document S2. Article plus supplemental information

## References

[1] Wang Q.H., Kalantar-Zadeh K., Kis A. (2012). Electronics and optoelectronics of two-dimensional transition metal dichalcogenides. Nat. Nanotechnol..

[2] Manzeli S., Ovchinnikov D., Pasquier D. (2017). 2D transition metal dichalcogenides. Nat. Rev. Mater..

[3] Zou X., Xu Y., Duan W. (2021). 2D materials: rising star for future applications. Innovation.

[4] Zhou W., Zou X., Najmaei S. (2013). Intrinsic structural defects in monolayer molybdenum disulfide. Nano Lett..

[5] Zhou J., Lin J., Sims H. (2020). Synthesis of Co-doped MoS_2_ monolayers with enhanced valley splitting. Adv. Mater..

[6] Zhu J., Wang Z.-C., Dai H. (2019). Boundary activated hydrogen evolution reaction on monolayer MoS_2_. Nat. Commun..

[7] Lin J., Fang W., Zhou W. (2013). AC/AB stacking boundaries in bilayer graphene. Nano Lett..

[8] Zhao X., Fu D., Ding Z. (2018). Mo-terminated edge reconstructions in nanoporous molybdenum disulfide film. Nano Lett..

[9] Li H., Tsai C., Koh A.L. (2016). Activating and optimizing MoS_2_ basal planes for hydrogen evolution through the formation of strained sulphur vacancies. Nat. Mater..

[10] Ly T.H., Perello D.J., Zhao J. (2016). Misorientation-angle-dependent electrical transport across molybdenum disulfide grain boundaries. Nat. Commun..

[11] Man P., Srolovitz D., Zhao J. (2021). Functional grain boundaries in two-dimensional transition-metal dichalcogenides. Acc. Chem. Res..

[12] van der Zande A.M., Huang P.Y., Chenet D.A. (2013). Grains and grain boundaries in highly crystalline monolayer molybdenum disulphide. Nat. Mater..

[13] Yu M., Zhu C., He Y. (2021). Polymorphism of segmented grain boundaries in two-dimensional transition metal dichalcogenides. Nano Lett..

[14] Batzill M. (2018). Mirror twin grain boundaries in molybdenum dichalcogenides. J. Phys. Condens. Matter.

[15] Xia Y., Zhang J., Jin Y. (2020). Charge density modulation and the Luttinger liquid state in MoSe_2_ mirror twin boundaries. ACS Nano.

[16] Barja S., Wickenburg S., Liu Z.-F. (2016). Charge density wave order in 1D mirror twin boundaries of single-layer MoSe_2_. Nat. Phys..

[17] Ma Y., Diaz H.C., Avila J. (2017). Angle resolved photoemission spectroscopy reveals spin charge separation in metallic MoSe_2_ grain boundary. Nat. Commun..

[18] Ouyang Y., Ling C., Chen Q. (2016). Activating inert basal planes of MoS_2_ for hydrogen evolution reaction through the formation of different intrinsic defects. Chem. Mater..

[19] Lin J., Pantelides S.T., Zhou W. (2015). Vacancy-induced formation and growth of inversion domains in transition-metal dichalcogenide monolayer. ACS Nano.

[20] Liu H., Jiao L., Yang F. (2014). Dense network of one-dimensional midgap metallic modes in monolayer MoSe_2_ and their spatial undulations. Phys. Rev. Lett..

[21] Jolie W., Murray C., Weiß P.S. (2019). Tomonaga-Luttinger liquid in a box: electrons confined within MoS_2_ mirror-twin boundaries. Phys. Rev. X.

[22] Yu H., Liao M., Zhao W. (2017). Wafer-scale growth and transfer of highly-oriented monolayer MoS_2_ continuous films. ACS Nano.

[23] Zhou J., Lin J., Huang X. (2018). A library of atomically thin metal chalcogenides. Nature.

[24] Zhao S., Lu M., Xue S. (2019). A Se vacancy induced localized Raman mode in two-dimensional MoSe2 grown by CVD. arXiv.

[25] Yin X., Ye Z., Chenet D.A. (2014). Edge nonlinear optics on a MoS_2_ atomic monolayer. Science.

[26] Karma A., Plapp M. (1998). Spiral surface growth without desorption. Phys. Rev. Lett..

[27] Zhu J., Xu H., Zou G. (2019). MoS_2_–OH bilayer-mediated growth of inch-sized monolayer MoS_2_ on arbitrary substrates. J. Am. Chem. Soc..

[28] Artyukhov V.I., Hu Z., Zhang Z. (2016). Topochemistry of bowtie- and star-shaped metal dichalcogenide nanoisland formation. Nano Lett..

[29] Meca E., Lowengrub J., Kim H. (2013). Epitaxial graphene growth and shape dynamics on copper: phase-field modeling and experiments. Nano Lett..

[30] Shi Y., Wang J., Wang C. (2015). Hot electron of Au nanorods activates the electrocatalysis of hydrogen evolution on MoS_2_ nanosheets. J. Am. Chem. Soc..

[31] Xia B., Wang T., Jiang X. (2018). Ar^2+^ beam irradiation-induced multivancancies in MoSe_2_ nanosheet for enhanced electrochemical hydrogen evolution. ACS Energy Lett..

[32] Zhu C., Yu M., Zhou J. (2020). Strain-driven growth of ultra-long two-dimensional nano-channels. Nat. Commun..

[33] Gong Y., Lin J., Wang X. (2014). Vertical and in-plane heterostructures from WS_2_/MoS_2_ monolayers. Nat. Mater..

[34] Wang X., Gong Y., Shi G. (2014). Chemical vapor deposition growth of crystalline monolayer MoSe_2_. ACS Nano.

[35] Yang T., Zheng B., Wang Z. (2017). Van der Waals epitaxial growth and optoelectronics of large-scale WSe_2_/SnS_2_ vertical bilayer p–n junctions. Nat. Commun..

[36] Liu L., Li T., Ma L. (2022). Uniform nucleation and epitaxy of bilayer molybdenum disulfide on sapphire. Nature.

[37] Wang J., Xu X., Cheng T. (2022). Dual-coupling-guided epitaxial growth of wafer-scale single-crystal WS_2_ monolayer on vicinal a-plane sapphire. Nat. Nanotechnol..

[38] Liu F., Chen K., Xue D. (2023). How to fast grow large-size crystals?. Innovation.

