# Supplementary material for: Non-epitaxial growth of highly oriented transition metal dichalcogenides with density-controlled twin boundaries
Source: Innovation (Camb). 2023 Aug 22;4(6):100502. doi: 10.1016/j.xinn.2023.100502 (PMC10493259; doi:10.1016/j.xinn.2023.100502)
Supplement: Document S1. Figures S1–S23, Tables S1 and S2, and Notes S1–S4 [file mmc1.pdf]

The Innovation, Volume *4*

Supplemental Information

Non-epitaxial growth of highly oriented transition metal dichalcogenides with density-controlled twin boundaries

Juntong Zhu, Zhili Hu, Shasha Guo, Ruichun Luo, Maolin Yu, Ang Li, Jingbo Pang, Minmin Xue, Stephen J. Pennycook, Zheng Liu, Zhuhua Zhang, and Wu Zhou

Methods

Engineering TBs in $MoSe_2$ and WSe_2 monolayers by OH-assisted CVD method.

We first dissolved 45 mg of ammonium molybdate tetrahydrate and 128 mg of KOH or NaOH in 10 ml of deionized water, forming Mo precursor, OH^- ions were used to guarantee the monolayer nature of the grown TMD[1]. The Mo precursor was then spin-coated onto a clean SiO_2/Si substrate (300 nm thick SiO_2 over Si) at 8000 rpm. Mo precursor-covered substrate was put into tube furnace, the details of the growth process and parameters are depicted in **Supplemental Figure 1**, the carrier gas is 5% Ar/H_2. TB engineered $MoSe_2$ monolayers were grown at 740, 770 and 800 °C for 3 minutes. The synthesis recipe for WSe_2 is similar to that of $MoSe_2$, where the sodium tungstate, sapphire and 10% Ar/H_2 are used as the W source, growth substrate and carrier gas, respectively. We found that the Mo precursors grown at 650 °C remain in their original state without any diffusion of Mo nucleation site (**Supplemental Figure 2c**). When the growth temperature rises to 660 °C, high density Mo nucleation sites start to diffuse out from the central mound (**Supplemental Figure 3**). Therefore, the investigated intermediate products were grown at 660, 670, 680, 710 and 740 °C (**Figure 3D-G**), being cooled directly after reaching the target temperature without maintaining a constant temperature. All the reactions were carried out under atmospheric pressure.

Growth of $MoSe_2$ monolayers by molten-salt-assisted CVD method.

A powder mixture of 3 mg MoO_3 and 0.5 mg NaCl in an aluminium oxide boat was placed in the centre of the quartz tube. We added a precipitation stage to the

temperature profile prior to TMDs growth at 650 °C, which exceeds the melting point of the MoO_3 and NaCl mixture, ensuring that sufficient metal source is pre-deposited onto the substrate to activate self-oriented growth. The typical Star-of-David $MoSe_2$ (**Figure 4E**) was produced at 740 °C without the precipitation stage at 650 °C.

TEM and STEM sample preparation.

We used a poly (methyl methacrylate) (PMMA) assisted method to prepare STEM samples. A thin layer of PMMA (Allersist, AR-P 672.045) film was spin coated onto the SiO_2/Si substrate and then baked at 85 °C for 10 min. The PMMA-covered substrate was then immersed in 2M NaOH solution for corroding SiO_2 layers of the substrate. The PMMA/sample film was immersed in deionized water and washed after the separation from the substrate. After that, the film was fished out by a Cu TEM grid (Quantifoil, 50 nm Cu foil of 200 mesh). Finally, the PMMA support layer was dissolved by soaking the grid in acetone at 80 °C for 2 minutes.

Material characterization.

The morphology was characterized by SEM (FEI Helios G4) and Raman spectroscopy (Alpha 300RS+, WITec). Raman and PL spectroscopy was performed with a 532nm laser whose power is ~1.5 mW at room temperature (Alpha 300RS+, WITec). SHG mapping was obtained under excitation from a 1064 nm laser with an average power of 20 mW (Rainbow 1064 OEM). AFM images were obtained using a Bruker Dimension Icon AFM system. Bright and dark-field TEM characterization was

carried out on JEOL 2100Plus TEM with an accelerating voltage of 80 kV. STEM measurements were performed on an aberration-corrected Nion U-HERMES100 microscope operating at 60 kV. The probe-forming semi-angle is 32 mrad, and the collection half angle for HAADF imaging is between 75 and 210 mrad. The beam current was about 3 pA for a single shot image. TGA measurements were produced on TGA 50.

Multi-grain Wulff construction algorithm.

The phase-field method can simulate multi-grain shapes such as the David star shape of MoS_2 flakes[27], but is not employed here due to its low efficiency under the situation where hundreds of $MoSe_2$ grains are involved in a single simulation. Instead, we use a self-developed multi-grain Wulff construction algorithm, which is more efficient to predict the growth morphology of multi-grain systems without growth instabilities (thus the growth shape of any standalone single grains is simply their Wulff shape). For the ease of description, the single-grain Wulff shape is designated to be the equilibrium shape of TMDs, *i.e.* triangle, in the following discussion.

In the algorithm, N grains coexist in a 2D canvas. Each grain has a fixed center. An order parameter φ is used to represent the state of each pixel/point of the canvas, where $\varphi = 1$ means the coverage of grains and $\varphi = 0$ denotes the bare substrate. Initially each grain occupies a predefined area, or a nucleus. The jth edge of the ith grain moves away from the grain center according to,

$$\dot{l_{ij}} = k(\xi_{ij} - \xi_{eq}),$$

where l_{ij} is the distance between the edge and the grain center (*i.e.*, growth rate), k the kinetic constant, ξ the dimensionless supersaturation, ξ_{eq} the equilibrium value of ξ, and ξ_{ij} the mean ξ along the edge exposed to the bare substrate. At each pixel, the dimensionless supersaturation $\xi = c/c_s$, where c is the feedstock concentration, and c_s is the nominal concentration of precursor in solid 2D crystals. In each time step of the simulation, if a pixel of the canvas has $\varphi=0$ and is inside the enclosure of the three edge extension lines of grain i, where $i=1,2,\ldots,N$, and at least one of its neighboring pixels is already occupied by grain i, then this pixel belongs to grain i.

Meanwhile, the evolution of ξ needs to be solved by the equation,

$$\dot{\xi} = \nabla(D\nabla\xi) - \frac{\xi}{\tau_s} + F - \frac{\delta\varphi}{\delta t},$$

where F is the deposition flux, D is the diffusion coefficient of ξ, τ_s is the desorption time, and the last term denotes the rate of the change of φ.

Phenomenological simulation setups.

A 200×200 canvas is used in each simulation. Initially, a central grain is placed at the center pixel of the canvas and its orientation is fixed to 30° such that a tip always points upward (the north direction). This grain is also a permanent part of the central “continent”. Except for the central grain, each satellite grain is initially assigned with a random grain orientation and occupies a random pixel. Once a satellite grain attaches to the “continent”, it self-orients by rotation such that it differs from its neighboring grain by either 60° or 0°. The initial value of ξ is 0.1 everywhere. To mimic the effect of self-oriented growth, ξ within a distance of 5 pixels to the canvas center is set to a

value ξ_c, where $\xi_c = t$ when t<50, and ξ_c=50 otherwise. To exhibit the effect of a non-uniform diffusivity D, we set D=100 where φ=1, and D=0.01 where φ=0. We further simplify the simulation by presuming the small growth rates for all edges of each grain. Different k is tested to examine the effect of temperature. Other parameters in simulations include N=200, F=0, τ_S=1, and ξ_{eq}=0.1. With a time step of 1 and a grid size of 1, the simulation is solved with the finite difference method.

First-principles calculations of diffusion coefficients.

First-principles calculations were performed using the Vienna Ab-initio Simulation Package (VASP) code[2], with the Perdew-Burke-Ernzerh (PBE) parametrization of the generalized gradient approximation (GGA) as the exchange correlation potential and projector-augmented wave (PAW) method for the core region. The kinetic energy cutoff was chosen to be 400 eV for the plane-wave expansion, and a vacuum region of 20 Å was set to avoid spurious interaction between adjacent slabs. Structures were fully relaxed until the force on each atom was less than 0.01 eV/Å. The Brillouin zone integration was sampled by a 5 × 5 × 1 k-grid mesh for the $MoSe_2$ and SiO_2 supercells with lattice constants of ~15 Å. The energy barriers of Mo_3O_9 migration were computed with the climbing image nudged elastic band (NEB) method.

According to the random walk model for diffusion, the diffusion coefficient, D_{H_a}, of activated Mo_3O_9 molecules can be computed as

$$D_{H_a} = \frac{va^2}{z},$$

where a is the traveling distance of Mo_3O_9 in a single hop, v denotes the microscopic

jump frequency, and z is the number of neighboring sites to which Mo_3O_9 can hop. According to the transition state theory, the microscopic jump frequency v is related to the activation energy E_{act} for surface diffusion, determined by the following equation

$$v = \frac{kT}{h}\exp\left(\frac{-E_{act}}{kT}\right),$$

where T is temperature, k the Boltzmann constant, and h the Planck constant.

Fabrication of micro-electrochemical device and electrocatalytic measurement.

First, we grew large-scale continuous graphene films on the Cu foils by the CVD method. Second, the as-grown graphene films were transferred onto pre-patterned chips using the PMMA-assisted method, then as-grown $MoSe_2$ monolayers were transferred on the graphene surface. Third, an annealing process at 200 °C under high-vacuum conditions (1×10^{-5} torr) was used to eliminate residual molecules between the graphene and MoS_2 films to obtain optimized interfaces. Fourth, e-beam lithography (EBL) was used to fabricate the electrodes (Cr/Au, 2 nm/60 nm) on graphene to connect the device. Fifth, a 500 nm-thick layer of PMMA film was spin-coated on the device chip, and then EBL was employed to remove the PMMA film from the interested region where six-point star or hexagonal $MoSe_2$ flakes exist, making sure it is the only exposed region of the active catalyst in the HER test.

The typical four-electrode micro-electrochemical measurements were conducted in a 0.5M H_2SO_4 electrolyte solution. The scan rate was set to be 5 mV per step. The electrocatalytic current and conductance current are simultaneously detected.

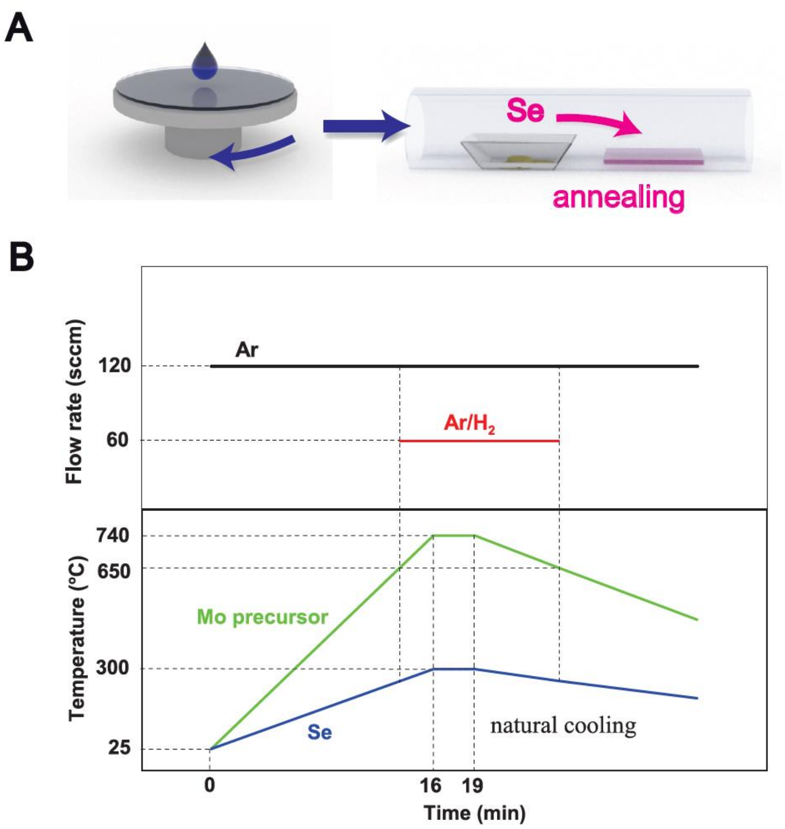

Figure S1 Parameters for the growth of $MoSe_2$ by OH-assisted CVD method. **(A)** Schematic diagram of OH-assisted CVD method, including spin-coating Mo precursor solution and annealing under Se atmosphere. **(B)** Detailed growth parameters of OH-assisted CVD method. When the temperature rises to 650 °C, Se powders partially evaporate into the ambient air. At the same time, the introduced reducing gas (H_2) starts to initiate the reaction between Se and MoO_x precursors. With the increase of temperature, the growth rate of $MoSe_2$ increases and eventually forms the highly crystalline monolayer $MoSe_2$.

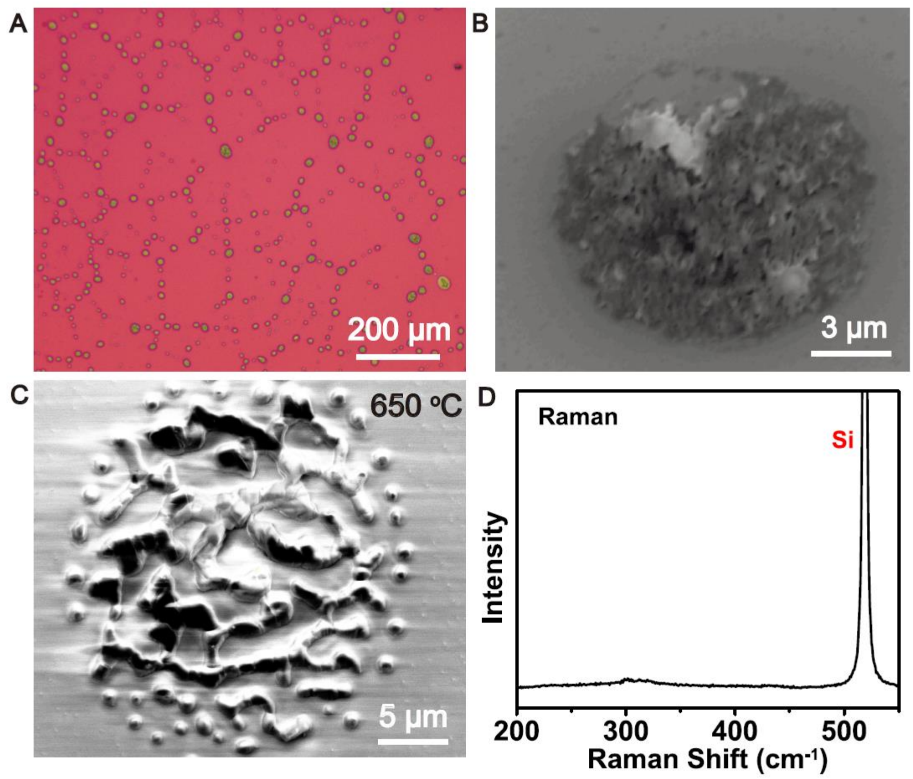

Figure S2 Morphology of Mo precursor after spin-coating and annealed at 650 °C. **(A and B)** Optical and SEM images of spin-coated Mo precursor. Due to the poor hydrophilicity of SiO_2/Si, the Mo precursor solute is deposited on the substrate surface in the form of large mounds immediately after evaporation of water. **(C)** SEM image of Mo precursor annealed at 650 °C, remaining in their original state without the diffusion of tiny Mo-contained particles. **(D)** Raman spectrum of 650 °C-grown samples absents the characteristic peaks of $MoSe_2$.

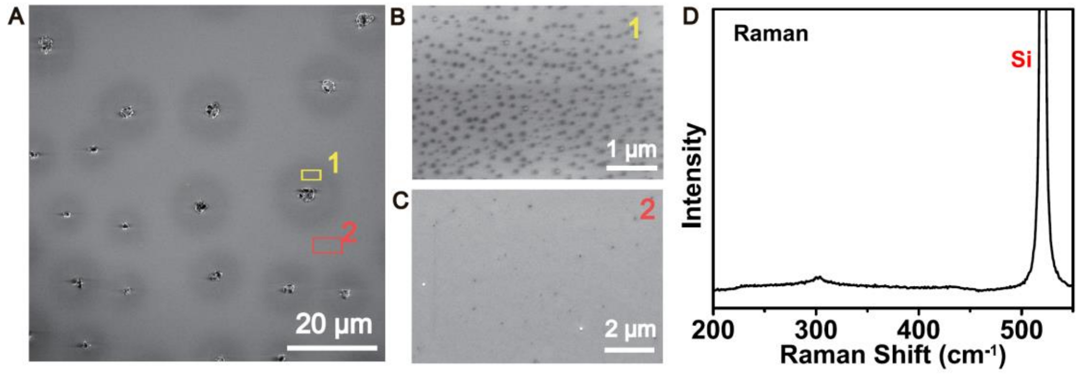

Figure S3 Morphology of Mo precursor annealed at 660 °C. **(A)** Low-magnification SEM image of Mo precursor annealed at 660 °C. **(B and C)** Magnified SEM image of region 1 and 2 in **A**. A high-density of Mo nucleation sites begins to diffuse out from central mounds at 660 °C. **(D)** Raman spectrum of the annealed Mo precursor indicates that the diffused particles have not formed $MoSe_2$ crystals.

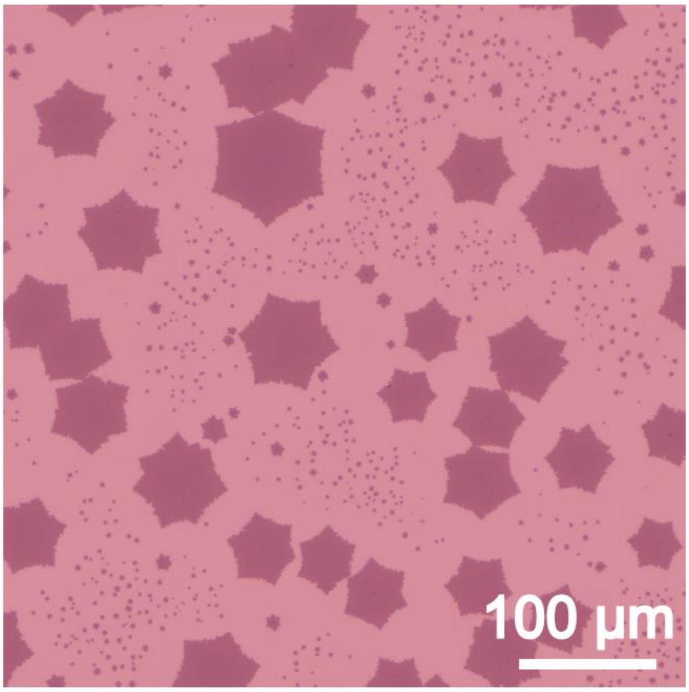

Figure S4 Optical image of the $MoSe_2$ grown at 740 °C for 3 min. The average transverse length of the fuzzy hexagonal $MoSe_2$ flake is about 50 µm.

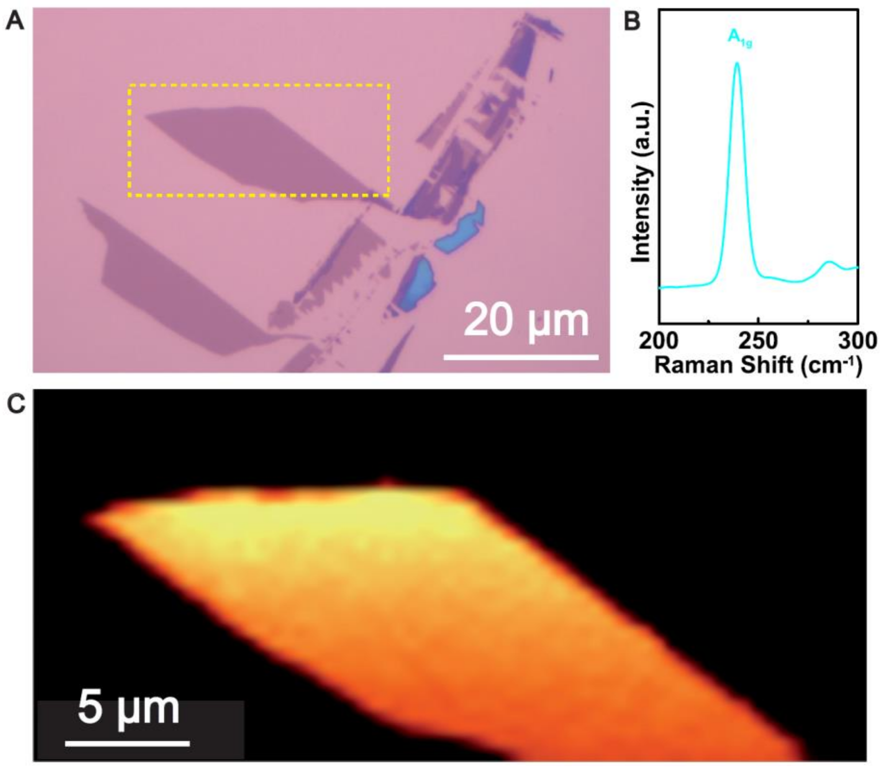

Figure S5 Morphology and spectroscopy of exfoliated $MoSe_2$ single-crystal. **(A)** Optical image of exfoliated $MoSe_2$ nanosheet. **(B)** Raman spectrum of exfoliated $MoSe_2$. **(C)** SHG image of exfoliated $MoSe_2$ in **A**. The uniform contrast indicates the single-crystal nature of the exfoliated $MoSe_2$.

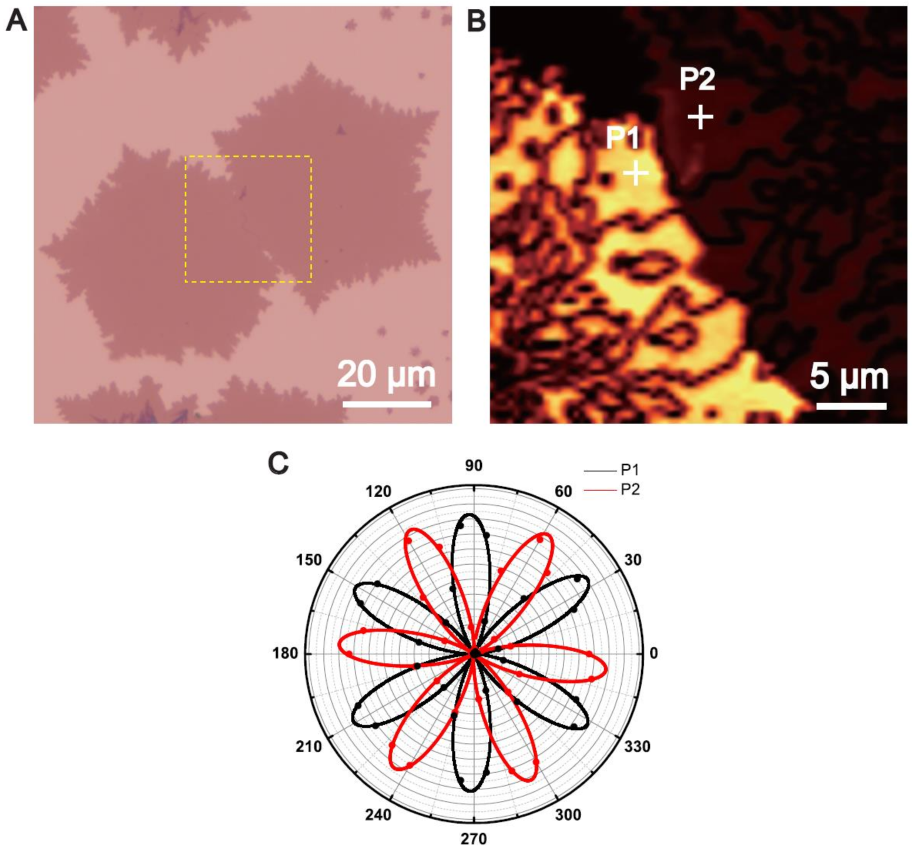

Figure S6 Spectroscopy of as-grown TB-rich $MoSe_2$. (A) Optical image of as-grown $MoSe_2$ monolayers. **(B)** Polarized SHG images of the adjacent $MoSe_2$ flakes. (**C**) Polarization-resolved SHG spectra of adjacent flakes marked by the white crosses in **B**. The difference in orientation angle between the two flakes is about 25º

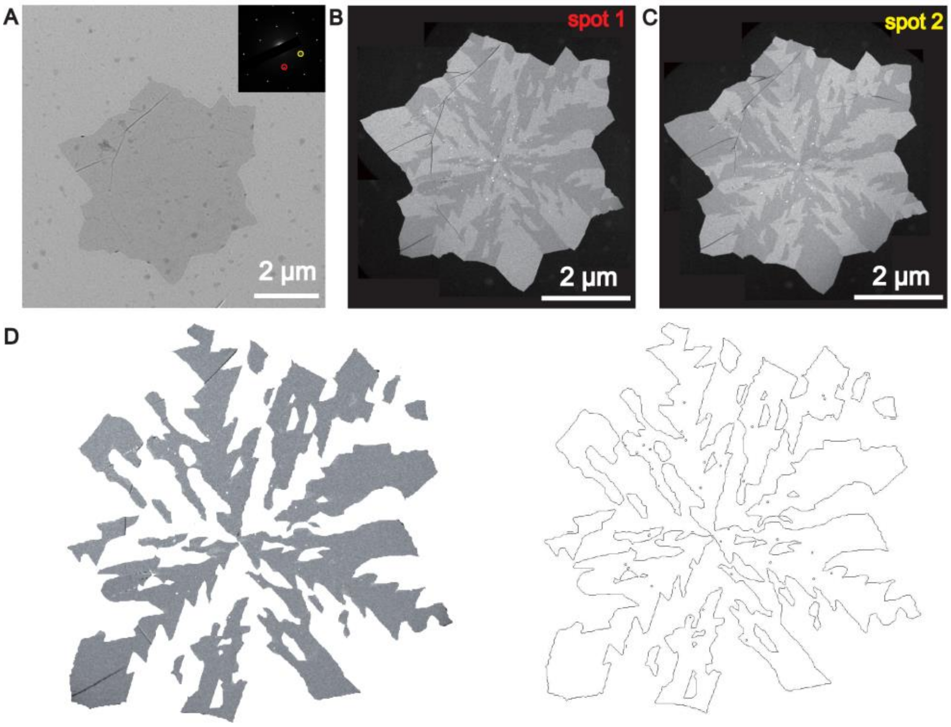

Figure S7 Structure of TB-rich $MoSe_2$ flake. **(A)** Bright-field image of 740 °C-grown $MoSe_2$ specimen, with diffraction pattern inseted. **(B and C)** Dark-field images collected from the selected diffraction spots in the inseted diffraction pattern of **A**. **B** and **C** show the opposite contrast, confirming their twinned structure. **(D)** Calculation of total length of TB in $MoSe_2$ (**A**).

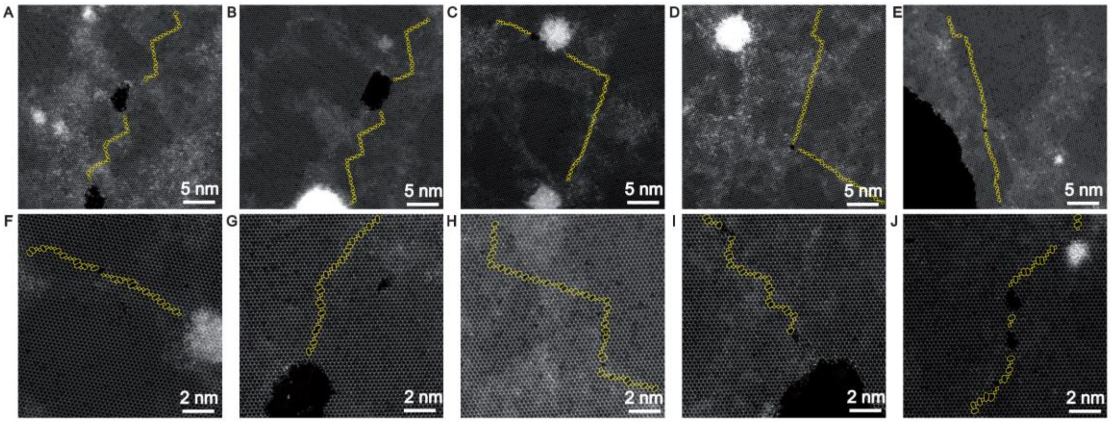

Figure S8 Microstructure of TBs in 10 different areas. **(A-J)** STEM images show the TBs with a high percentage of 8-membered rings.

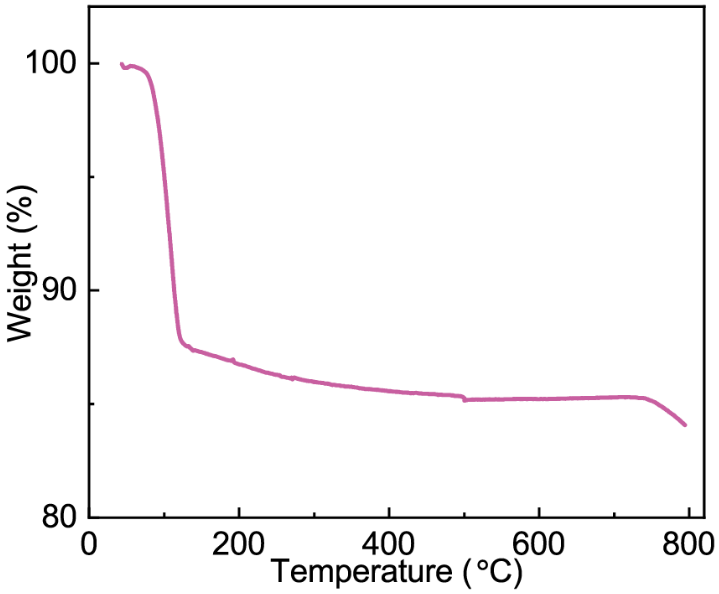

Figure S9 TGA curve of Mo precursor from room temperature to 800 °C.

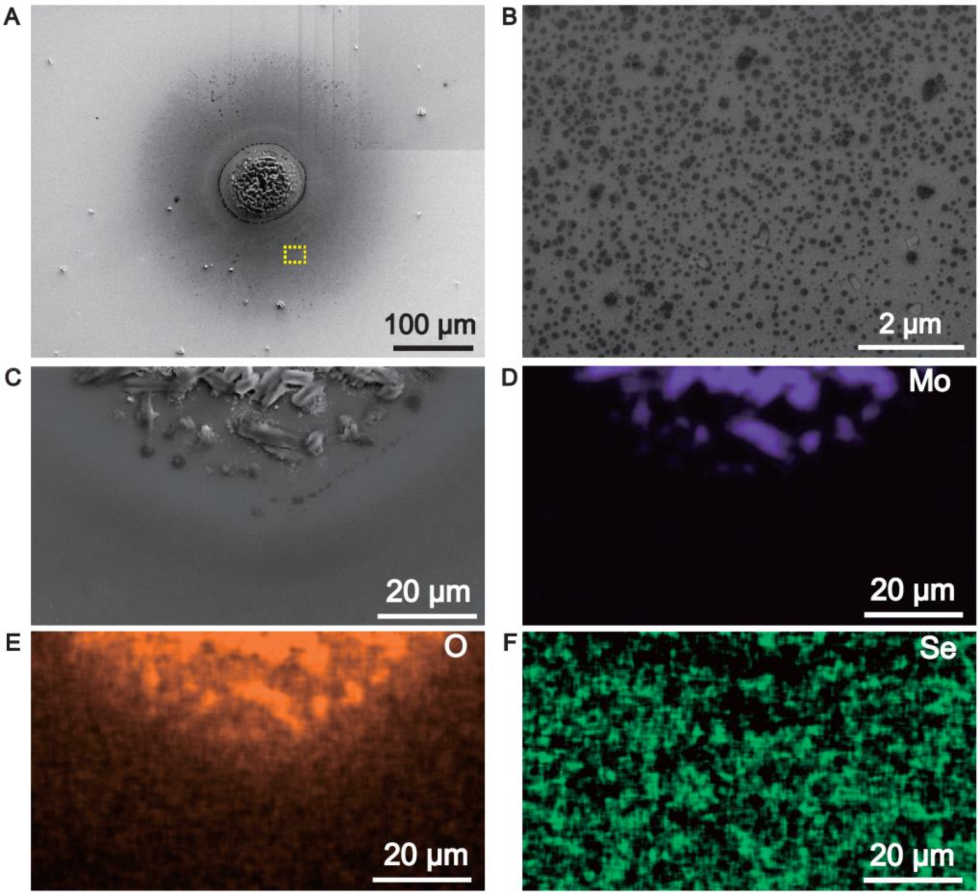

Figure S10 Structure of 660 °C-grown $MoSe_2$ on Si substrate. **(A)** Low-magnification SEM image of 660 °C-grown $MoSe_2$ on Si substrate. **(B)** The magnified SEM image of the marked region in **A** shows a high-density of tiny particles around the Mo precursor mound. **(C-F)** SEM image (**c**) and corresponding EDX maps of Mo (**D**), O (**E**) and Se (**F**) elements. The inner mound shows obvious Mo and O signals but a low Se signal intensity, indicating its MoO_x structure. Meanwhile, the Se signal intensity of the surrounding tiny particles is significantly stronger than that of the inner Mo precursor mound, confirming the selenization of diffused tiny Mo particles, which prevents the Ostwald ripening and generates a high density of diffused tiny particles. It is worth noting that, in order to exclude the influence of substrate on the distribution of O, the $MoSe_2$ is grown on Si substrate. The as-grown samples have the same morphology as those grown on SiO_2 substrate (**Figure S3**).

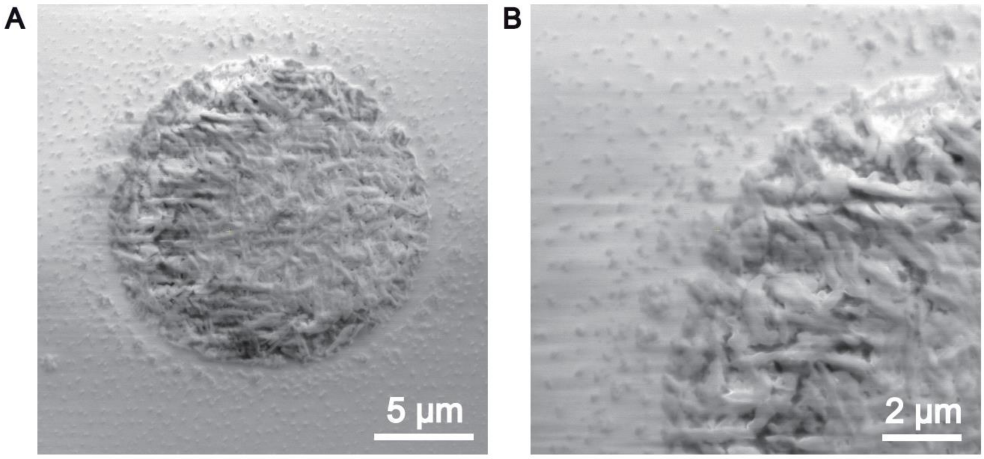

Figure S11 Structure of 660 °C-grown $MoSe_2$ without Se atmosphere. **(A)** Low-magnification SEM image of 660 °C-grown $MoSe_2$ on Si substrate. **(B)** The magnified SEM image shows sparse large-sized particles around the central mound and no dense tiny Mo feedstock.

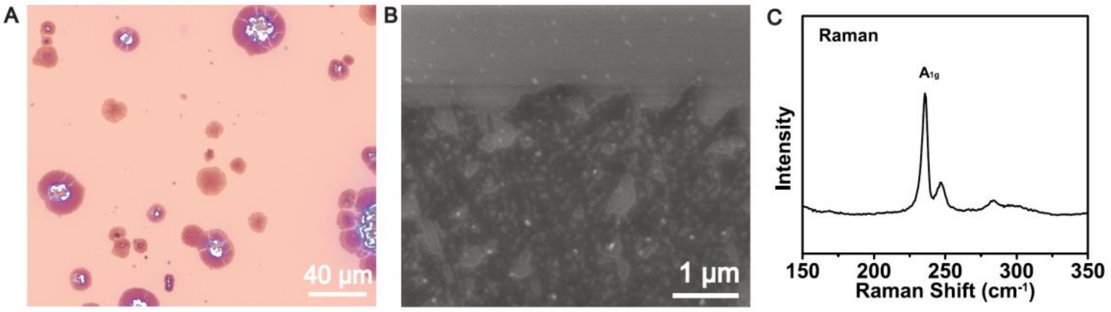

Figure S12 Structure of 670 °C-grown $MoSe_2$. **(A)** Low-magnification optical image of 670 °C-grown $MoSe_2$. Tiny grains grow around the large Mo precursor mounds. **(B)** Enlarged SEM image of circular $MoSe_2$. The loosely packed rounded rim of $MoSe_2$ contains many holes and tiny particles. **(C)** Raman spectrum of 670 °C-grown $MoSe_2$ with a typical peak at 239 cm^{-1}.

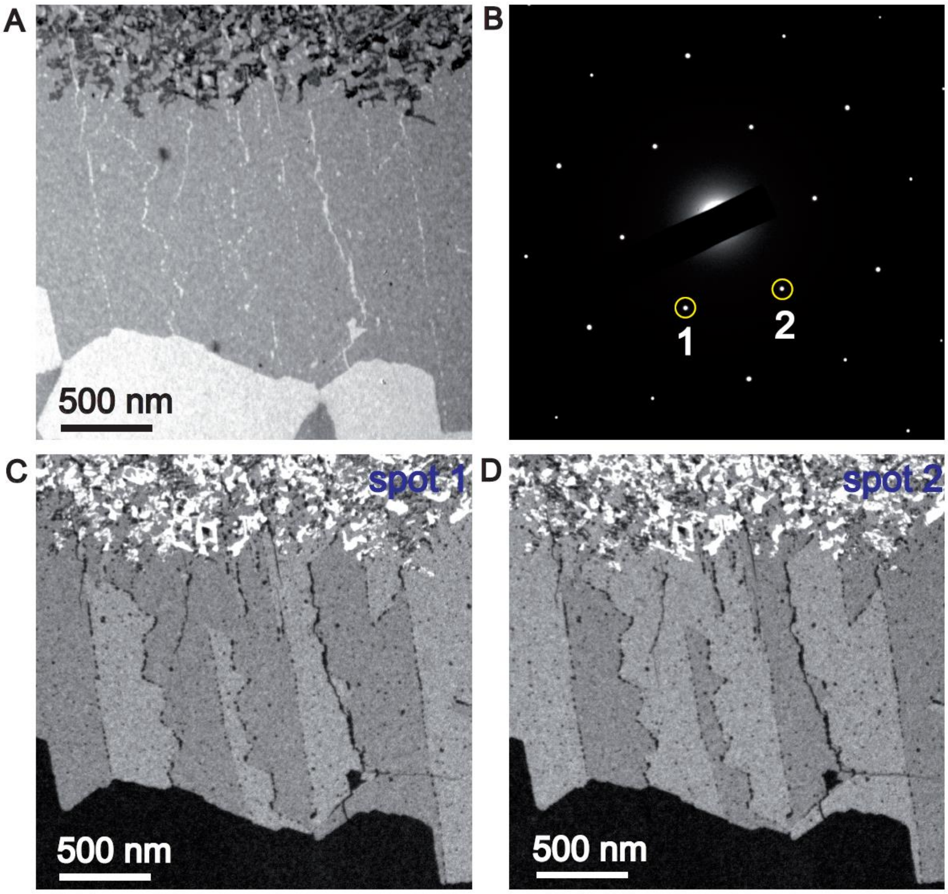

Figure S13 TEM images of 680 °C-grown $MoSe_2$. **(A)** Bright-field TEM image of as-grown $MoSe_2$ shows a relatively complete structure. **(B)** electron diffraction pattern of $MoSe_2$ in **A**. **(C and D)** Dark-field images collected from the selected diffraction spot 1 (**C**) and 2 (**D**) in **B**, respectively. The opposite contrast of **C** and **D** is consistent with their twin structure. The above results show that once the islands merge, TBs are formed.

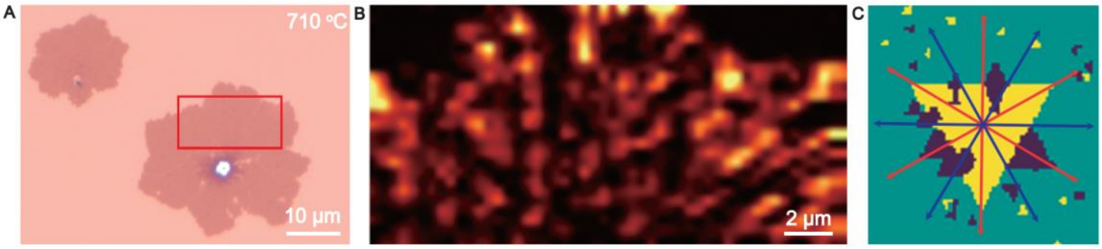

Figure S14 Structure of 710 °C-grown $MoSe_2$. **(A)** Optical image of an irregular shaped 710 °C-grown $MoSe_2$ with jagged edges, different from the rounded $MoSe_2$ grown at 680 °C. **(B)** SHG image of $MoSe_2$ grown at 710 °C shows high-density TBs and the twinned crystals show a nanoribbon shape. (**C**) Schematic diagram of a fuzzy Star-of-David shape appears.

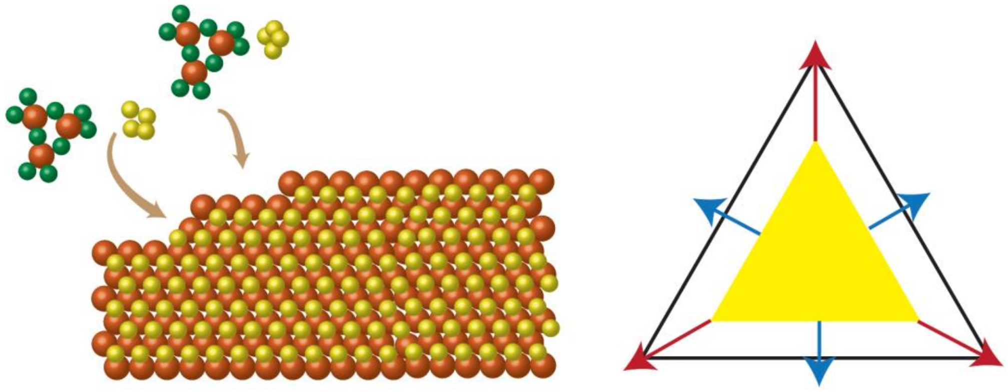

Figure S15 $MoSe_2$ grows epitaxially along the edges by vapor-phase metal sources. When the growth temperature is above the melting point of Mo precursor, vapor-phase Mo sources become the main source of Mo feedstock. The ratio of the growth rate along the three tip directions (red arrows) to the three edge directions (blue arrows) is 2:1.

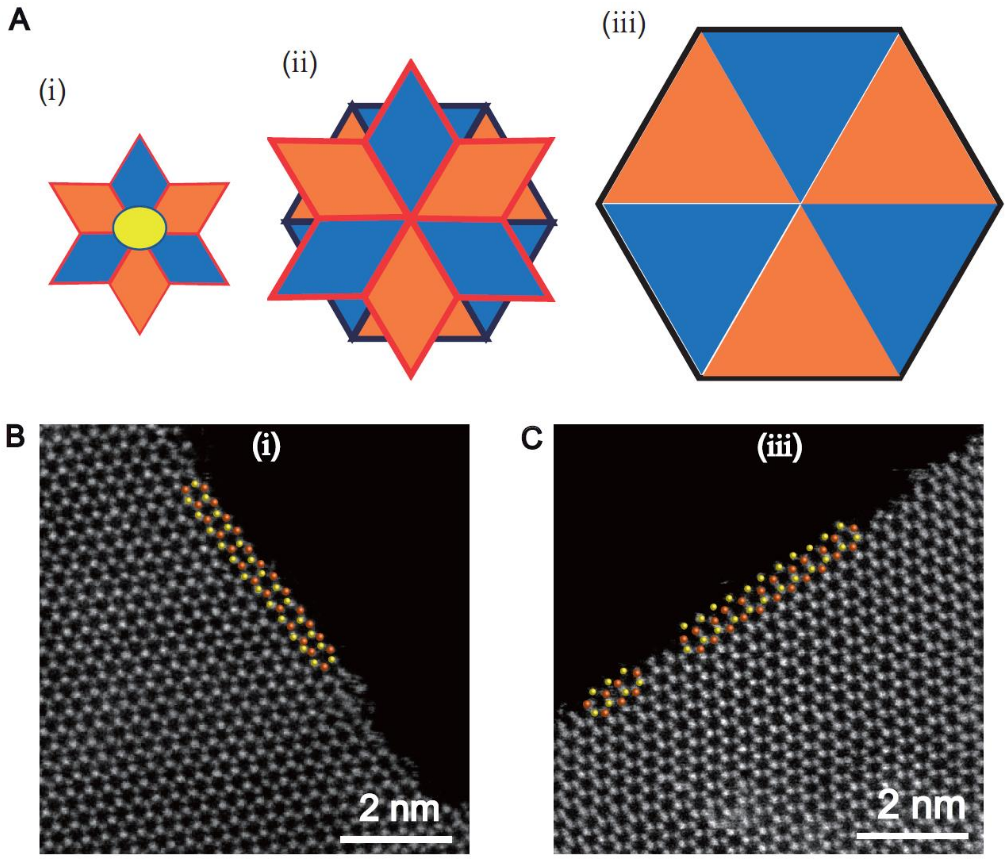

Figure S16 Edge termination of fuzzy David star (740 °C-grown sample) and hexagonal $MoSe_2$ (800 °C-grown sample). (A) (**i**) At a temperature below 740 °C, $MoSe_2$ self-oriented grows in a Mo-rich environment (large Mo precursor mounds provide a sufficient source of Mo) and forms a Mo termination as a matter of course; (**ii**) At higher growth temperatures above 740 °C, the mound is quickly exhausted, resulting in a reduction in the abundance of Mo feedstock. Consequently, once Mo feedstock becomes too scarce compared to Se, the edge termination of the subsequently grown $MoSe_2$ is Se; (**iii**) When the growth of $MoSe_2$ occurs in a Se-rich environment, the final sample with Se termination has a hexagonal shape. The red and black lines in edge refer to the Mo and Se terminations, respectively. **(B and C)** Edge termination of 740 °C-grown (**B**) and 800 °C-grown samples (**C**).

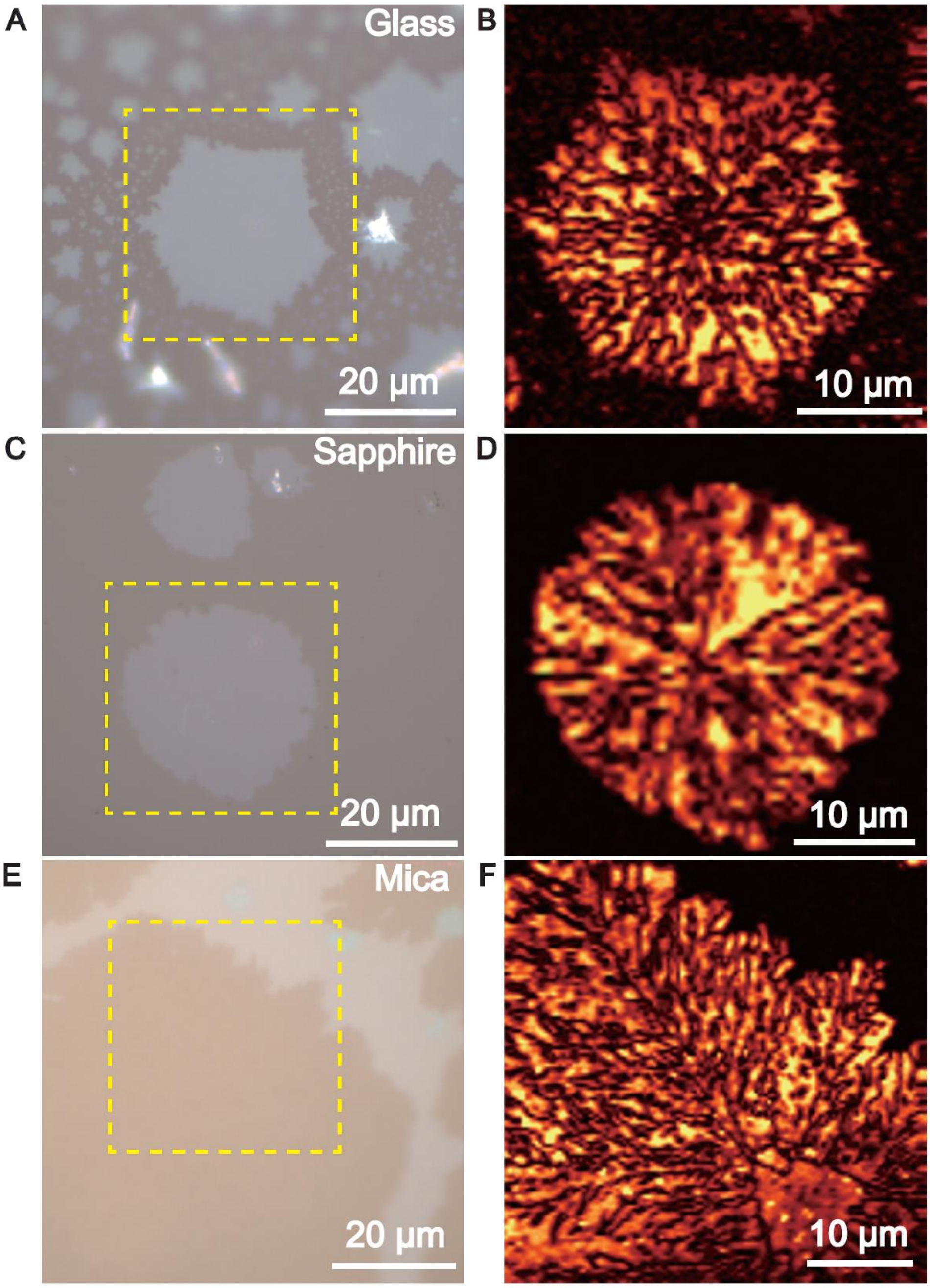

Figure S17 TB-rich $MoSe_2$ grown on arbitrary substrates. (A and B) Optical and SHG images of $MoSe_2$ grown on glass. $MoSe_2$ hexagon contains high density TBs. **(C and D)** Optical and SHG images of $MoSe_2$ grown on sapphire. $MoSe_2$ circle contains high density TBs. **(E and F)** Optical and SHG images of $MoSe_2$ grown on mica. Large-size $MoSe_2$ domain contains high density TBs.

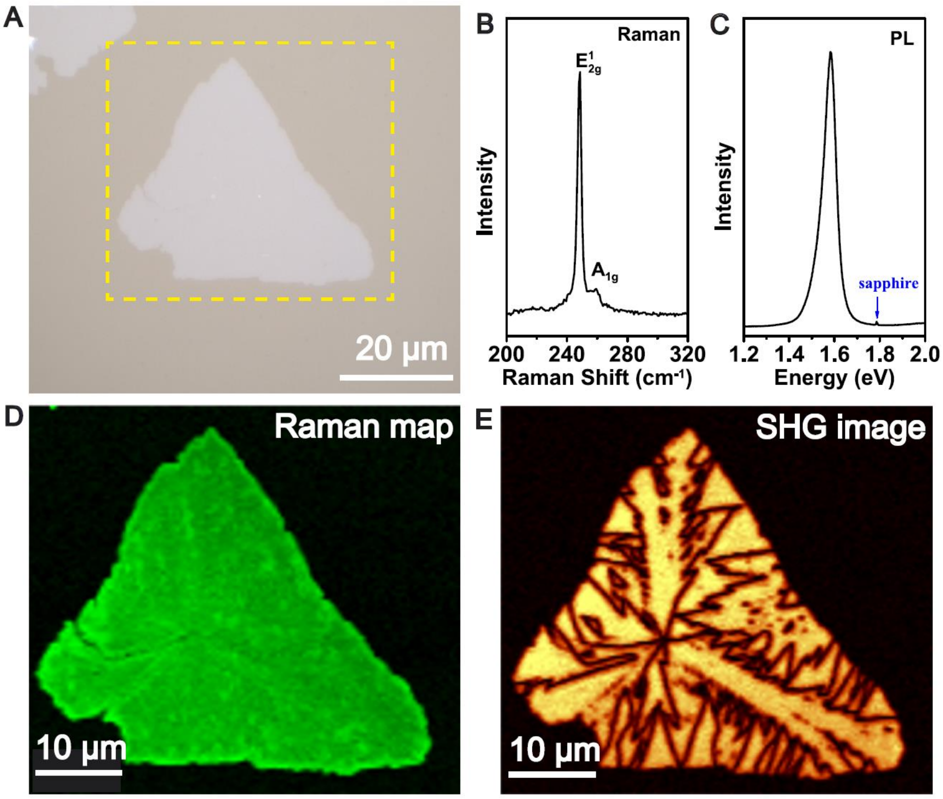

Figure S18 Morphology and spectroscopy of TB-rich WSe_2. (A) Optical image of WSe_2 grown by OH-assisted CVD method. **(B)** Raman spectrum of WSe_2 monolayers. The obvious typical peak at 240 cm^{-1} belongs to the in-plane E^1_{2g} mode of natural WSe_2. **(C)** PL spectrum of WSe_2 monolayers confirms its high crystallinity. **(D)** Raman intensity mapping of the E^1_{2g} peak. The uniform intensity demonstrates the high degree of uniform spectroscopic quality of as-grown WSe_2 flake. **(E)** SHG image of triangular WSe_2 flake. A high-density of TB in WSe_2 is similar to the as-grown $MoSe_2$.

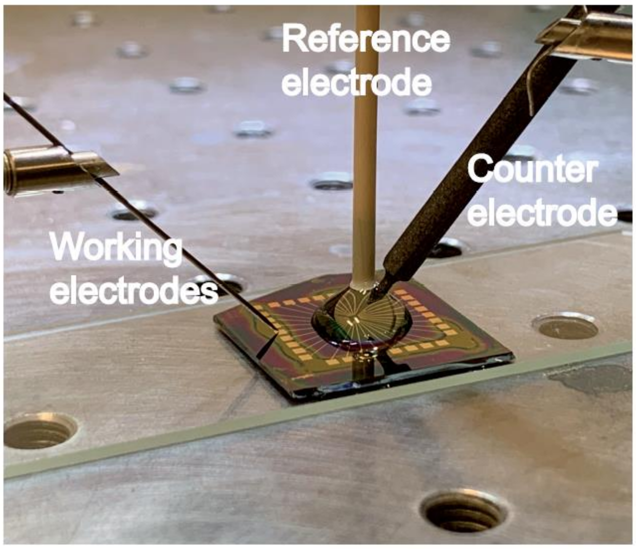

Figure S19 Photograph of a micro-electrochemical cell.

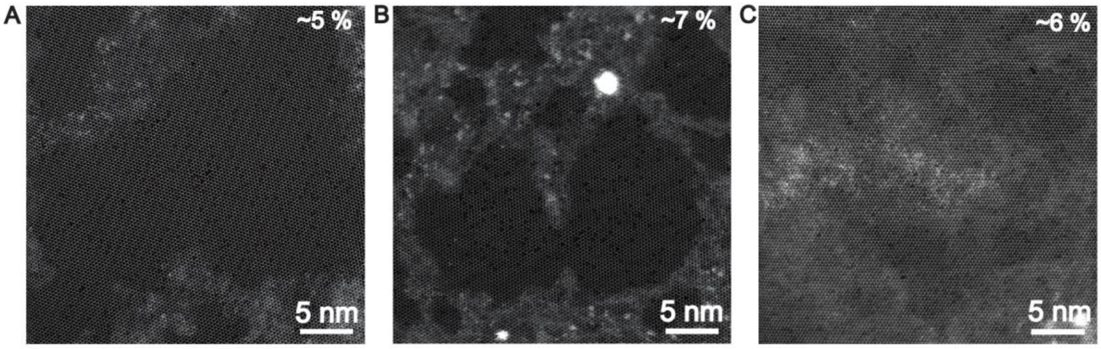

Figure S20 Atomic structure of the resultant $MoSe_2$ monolayers basal planes. High resolution STEM images of (**A**) 740 °C, (B) 800 °C and (**C**) David star shaped CVD-grown $MoSe_2$. The Se vacancy concentrations of 740 °C, 800 °C and CVD-grown $MoSe_2$ are 5%, 7% and 6%, respectively. The Se vacancy concentration in these samples are qualitatively similar, suggesting that the major catalytic contributors are TBs with 8-membered rings, while the contributions from Se vacancies and the pristine $MoSe_2$ basal plane are negligible.

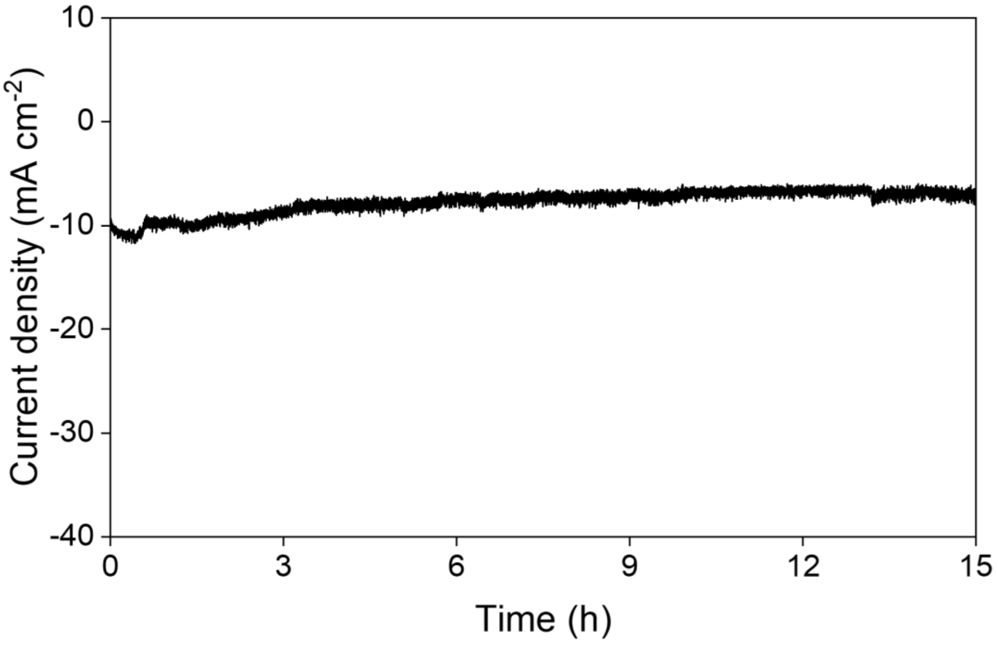

Figure S21 Electrochemical stability test of 740 °C-grown-$MoSe_2$ monolayers.

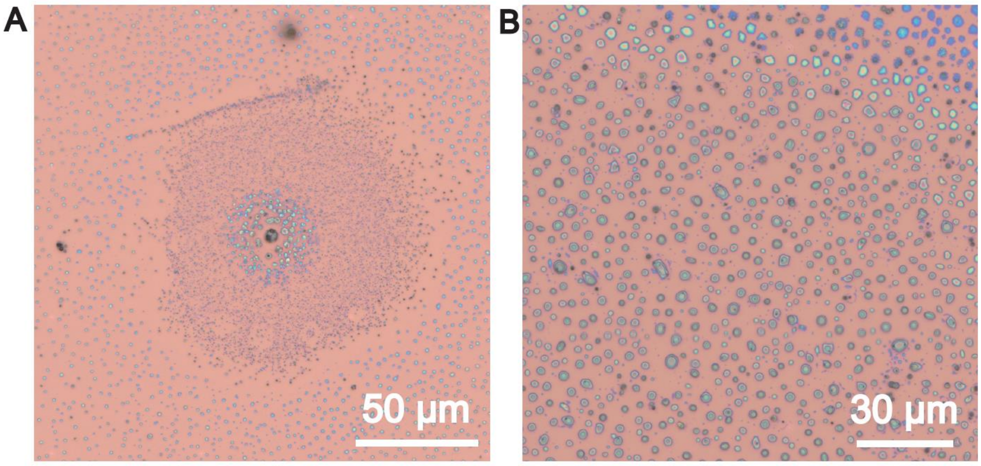

Figure S22 Morphology of intermediate products grown by CVD method at 650 °C. (A and B) Optical images of different regions. The abundant MoO_x particles precipitated onto the substrate surface.

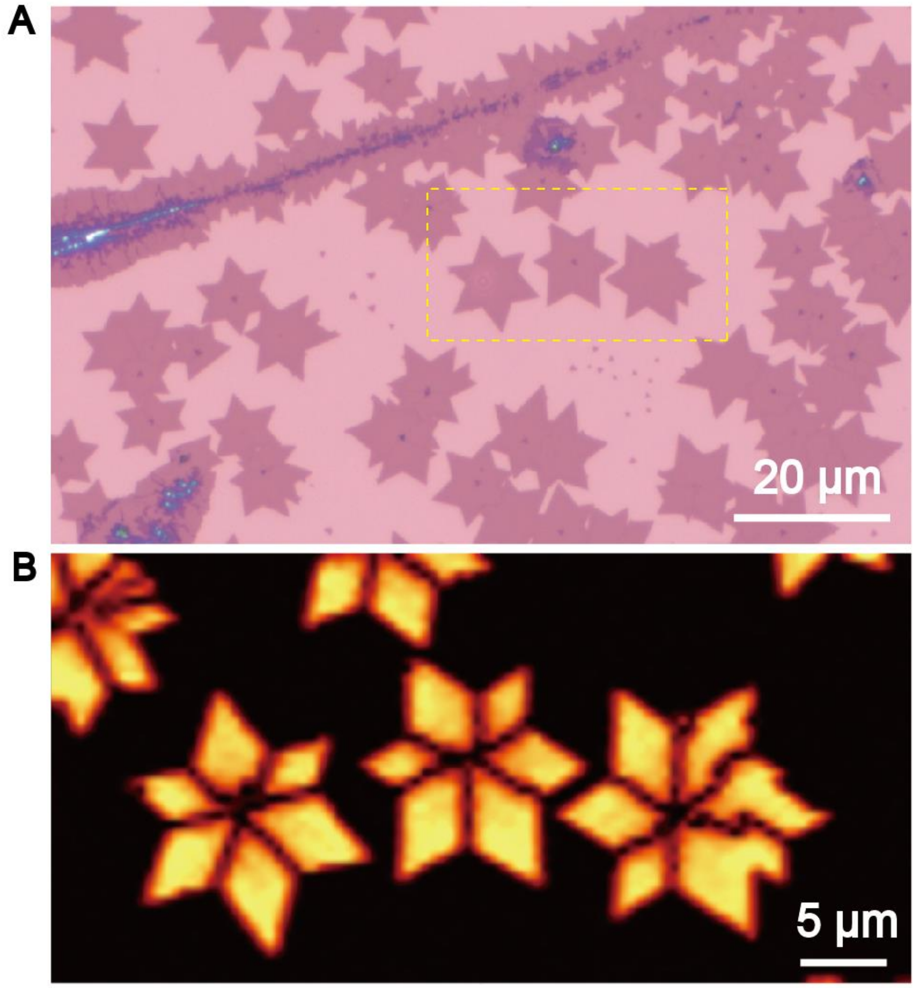

Figure S23 Morphology and spectroscopy of $MoSe_2$ grown by CVD method without precipitation stage. **(A)** Optical image of typical David star shaped $MoSe_2$. **(B)** Corresponding SHG image of these David stars in **A** shows that they only have six TBs.

T		D (m^2/s)
1000K	$MoSe_2$	6.42504E-08
	SiO_2 (glass)	3.96233E-18
	Sapphire	1.75183E-26

Table S1 Diffusion coefficient of Mo_3O_9 on the surface of $MoSe_2$, SiO_2 (glass) and sapphire at 1000 K. Due to the immense difference in diffusivities of metal feedstocks on various growth substrates and the as-formed TMD surface, high-density of TBs can be formed by the self-oriented nucleation and growth strategy.

MoS_2 or $MoSe_2$ Monolayers	η10	Tafel	References
GB-rich MoS_2 (random 4, 5, 7, 8-membered rings)	-325	95	[3]
GB-rich MoS_2 (1-3 layers)	-250	110	[4]
Segmented MoS_2 grain boundaries	-240	75	[5]
MoS_2 GB nano-channel	-266	90	[6]
Janus SMoSe	-250		[7]
TB-rich $MoSe_2$ (1D 4-membered rings)	-620	115	[8]
TB-rich $MoTe_2$ (1D 4-membered rings)	-520	72	[8]
**TB-rich $MoSe_2$ (high-density 8-membered rings)**	**-195**	**70**	**This work**

Table S2 HER performances of other grain boundary engineered TMDs.

Supplementary Notes

Supplementary Note 1

Calculation of the length of the TB per unit area. To get the total boundary length, we used Photoshop software to cut out the darker part of the flake, and used the Laplacian second-order zero-cross operator in MATLAB software to detect its edges, resulting in a domain boundary outline totaling 16560 pixels (**Figure S7d**). Considering that the 2-micron ruler occupies 380 pixels, we can calculate that the total boundary length is about 87.2 microns. The entire area of this flake is about 17.1 μm^2, so the length of the TB per unit area in our $MoSe_2$ is about 5.1 μm/μm^2. We also used the same method to calculate the TB length in typical MoS_2 David star grown on SiO_2/Si substrate[9] the highly-oriented MoS_2 film grown on the sapphire substrate[10], the results are 0.2 μm/μm^2 and 0.8 μm/μm^2, respectively.

Supplementary Note 2

Effect of temperature on the growth rate of $MoSe_2$, the diffusion rate of feedstocks and the kinetic constant (*k*). With the increase of growth temperature from T_1 to T_2, the growth rate of $MoSe_2$ increases by a factor of $\exp(-\Delta E_{\mathrm{growth}}/k_B T_2)\,/\exp(-\Delta E_{\mathrm{growth}}/k_B T_1) = \exp(\Delta E_{\mathrm{growth}}/k_B * (1/T_1 - 1/T_2))$. Likewise, the diffusion rate of feedstocks increases by a factor of $\exp(\Delta E_{\mathrm{diffuse}}/k_B * (1/T_1 - 1/T_2))$. Since $\Delta E_{\mathrm{growth}}$ is much larger than $\Delta E_{\mathrm{diffuse}}$, the growth rate should be significantly more sensitive to the temperature than the diffusion rate of feedstocks. Therefore, we focus on the effect of T on growth rates.

The influence of temperature on *k* can be inferred from Arrhenius equation[11], which formulates *k* as,

$$k = k^0 \exp(-E_a/k_B T)$$

where T is the local temperature, k_B is the Boltzmann constant, E_a is the activation energy, k^0 the prefactor.

According to

$$\dot{l_{ij}} = k(\xi_{ij} - \xi_{eq}),$$

we mentioned in **Methods**, the growth rate $(\dot{l_{ij}})$ can be represented by k, which is related to the growth temperature and follows an Arrhenius type behavior. We then provide a qualitative trend of k of 0.001, 0.01 and 0.1 at 660, 670 and 680 ℃. To obtain large-size simulation at 740 ℃, we used an extreme case to simplify the model, where the k can be considered as 1.

Supplementary Note 3

Formation process of $MoSe_2$ in a Star-of-David shape. To better understand the formation process of a fuzzy Star-of-David shape, we consider an extreme case where isolated islands cannot grow at all and suppose the Mo feedstock is excessive so that it distributes evenly on the continent surface. Indeed, the growth of $MoSe_2$ single crystals exhibits anisotropic behavior, with distinct growth rates along different directions. The ratio of the growth rate along the three tip directions to the three edge directions is 2:1 (**Figure S15**). In the case of a continent consisting of multiple grains with two opposite orientations (violet and yellow in **Figure S14C**), the radial growth rate of the continent away from its center is largest along six tip directions (red lines in **Figure S14C**) of either orientation (denoted as v_l), and smaller along bisectors of tip directions (denoted as v_s) (blue lines in **Figure S14C**), and the ratio is $v_l/v_s=\sqrt{3}:1$. As a result of these different growth rates, even though the precursor distribution is initially isotropic, the variation in growth rate among different directions can contribute to the formation of a fuzzy Star-of-David shape in the final samples.

Supplementary Note 4

Morphology evolution of $MoSe_2$ flakes from 740 °C to 800 °C. The diffusivity difference of Mo_3O_9 on SiO_2 and $MoSe_2$ surfaces strongly influences the growth morphology from 660 °C to 740 °C. A fuzzy David star shape $MoSe_2$ is grown at 740 °C. Interestingly, this fuzzy David star shape does not keep at an even longer growth time (740 °C for 3 min). In that case, the longer growth time of the continent makes Mo feedstock supplied by the central mound is no longer over abundant even for the growth of the continent itself. Then, the continent "coastline" closer to the center grows faster because it can obtain relatively more feedstock. This makes the flake shape more isotropic, resulting in a 6-point star shape (**Figure 4B$_1$**). However, when increase the annealing temperature over 740 °C, the transition of growth mode from self-oriented to edge-epitaxial is likely to reduce the abundance of Mo feedstock. Then, once Mo feedstock becomes too scarce compared to Se, the edge termination would switch from Mo to Se atoms (**Figure S16**), resulting in a multi-grain hexagon flake shape at 800 °C (**Figure 4D$_1$**).

References

1. Zhu, J., Xu, H., Zou, G. et al. (2019). MoS_2–OH bilayer-mediated growth of inch-sized monolayer MoS2 on arbitrary substrates. J. Am. Chem. Soc. ***141***, 5392-5401.
2. Kresse, G. & Furthmüller, J. (1996). Efficiency of ab-initio total energy calculations for metals and semiconductors using a plane-wave basis set. Comp. Mater. Sci. ***6***, 15-50.
3. Zhu, J., Wang, Z.-C., Dai, H. et al. (2019). Boundary activated hydrogen evolution reaction on monolayer MoS_2. Nat. Commun. ***10***, 1348.
4. He, Y., Tang, P., Hu, Z. et al. (2020). Engineering grain boundaries at the 2D limit for the hydrogen evolution reaction. Nat. Commun. ***11***, 57.
5. Yu, M., Zhu, C., He, Y. et al. (2021). Polymorphism of segmented grain boundaries in two-

dimensional transition metal dichalcogenides. Nano Lett. ***21***, 6014-6021.

6. Zhu, C., Yu, M., Zhou, J. et al. (2020). Strain-driven growth of ultra-long two-dimensional nano-channels. Nat. Commun. ***11***, 772.

7. Zhang, J., Jia, S., Kholmanov, I. et al. (2017). Janus monolayer transition-metal dichalcogenides. ACS Nano ***11***, 8192-8198.

8. Kosmala, T., Coy Diaz, H., Komsa, H. P. et al. (2018). Metallic twin boundaries boost the hydrogen evolution reaction on the basal plane of molybdenum selenotellurides. Adv. Energy Mater. ***8***, 1800031.

9. van der Zande, A. M., Huang, P. Y., Chenet, D. A. et al. (2013). Grains and grain boundaries in highly crystalline monolayer molybdenum disulphide. Nat. Mater. ***12***, 554-561.

10. Yu, H., Liao, M., Zhao, W. et al. (2017). Wafer-scale growth and transfer of highly-oriented monolayer MoS_2 continuous films. ACS Nano ***11***, 12001-12007.

11. Tabatabaei, F., Boussinot, G., Spatschek, R. et al. (2017). Phase field modeling of rapid crystallization in the phase-change material AIST. J. Appl. Phys. ***122***, 045108.
