# Supplementary material for: Non-epitaxial growth of highly oriented transition metal dichalcogenides with density-controlled twin boundaries
Source: Innovation (Camb). 2023 Aug 22;4(6):100502. doi: 10.1016/j.xinn.2023.100502 (PMC10493259; doi:10.1016/j.xinn.2023.100502)
Supplement: Document S2. Article plus supplemental information [file mmc2.pdf]

ARTICLE

www.the-innovation.org

Non-epitaxial growth of highly oriented transition metal dichalcogenides with density-controlled twin boundaries

Juntong Zhu,[1,5,*] Zhili Hu,[2,5] Shasha Guo,[3,5] Ruichun Luo,[1] Maolin Yu,[2] Ang Li,[1] Jingbo Pang,[1] Minmin Xue,[2] Stephen J. Pennycook,[1] Zheng Liu,[3,4,*] Zhuhua Zhang,[2,*] and Wu Zhou[1,*]

*Correspondence: zhujuntong@ucas.ac.cn (J.Z.); z.liu@ntu.edu.sg (Z.L.); chuwazhang@nuaa.edu.cn (Z.Z.); wuzhou@ucas.ac.cn (W.Z.)

Received: May 25, 2023; Accepted: August 21, 2023; Published Online: August 22, 2023; https://doi.org/10.1016/j.xinn.2023.100502

© 2023 The Authors. This is an open access article under the CC BY license (http://creativecommons.org/licenses/by/4.0/).

GRAPHICAL ABSTRACT

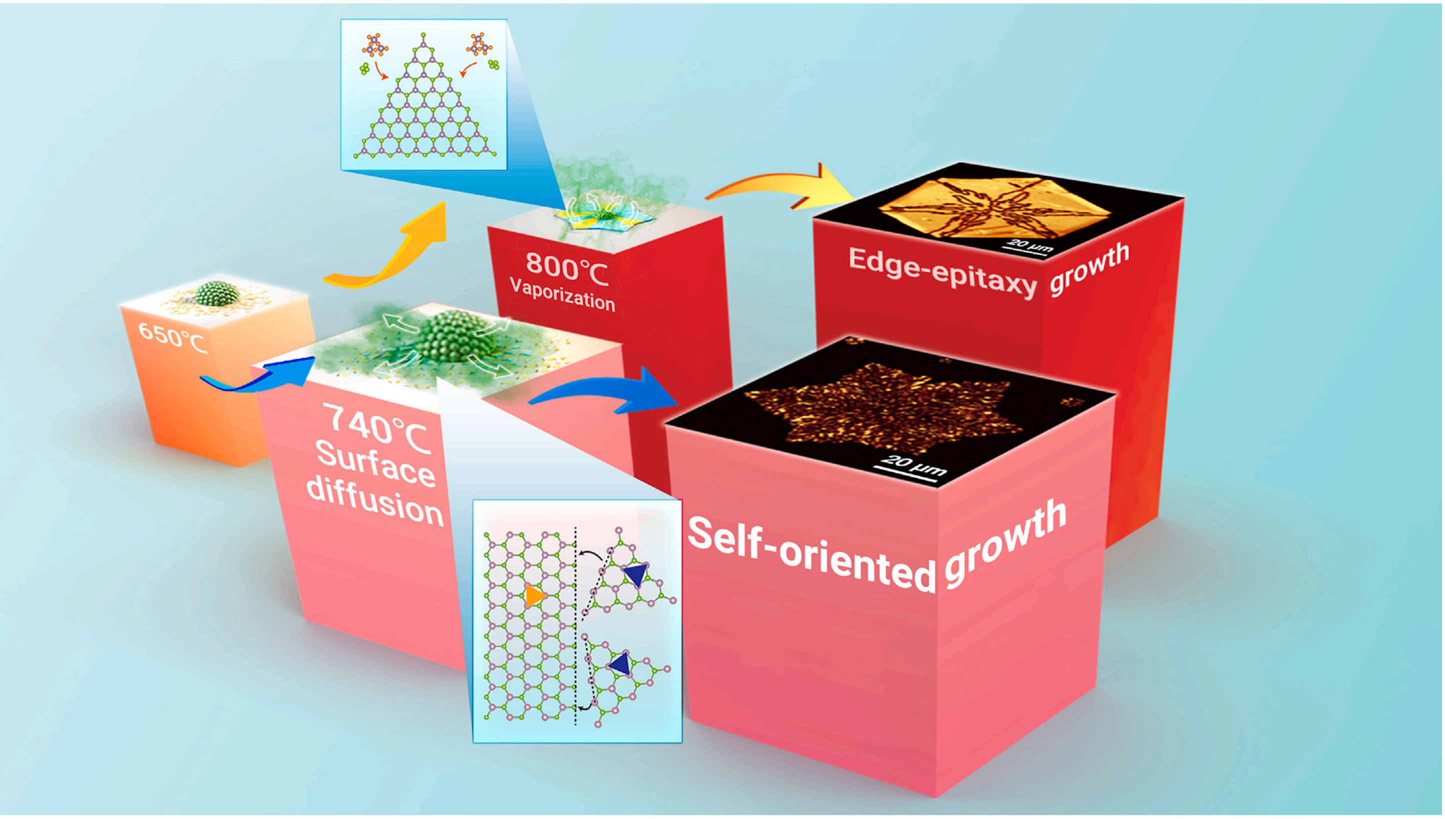

PUBLIC SUMMARY

- Differences in diffusivity of metal sources trigger the non-epitaxial growth of twin boundaries.
- The growth mechanism of high-density 1D twin boundaries on arbitrary substrates is revealed.
- A possible universal strategy for grain boundary engineering in 2D materials is proposed.
- Density-controllable twin boundaries provide a promising platform for exploring novel quantum states in 1D electronic systems.

Non-epitaxial growth of highly oriented transition metal dichalcogenides with density-controlled twin boundaries

Juntong Zhu,[1,5,*] Zhili Hu,[2,5] Shasha Guo,[3,5] Ruichun Luo,[1] Maolin Yu,[2] Ang Li,[1] Jingbo Pang,[1] Minmin Xue,[2] Stephen J. Pennycook,[1] Zheng Liu,[3,4,*] Zhuhua Zhang,[2,*] and Wu Zhou[1,*]

[1]School of Physical Sciences, CAS Key Laboratory of Vacuum Physics, University of Chinese Academy of Sciences, Beijing 100049, China

[2]State Key Laboratory of Mechanics and Control for Aerospace Structures, Key Laboratory for Intelligent Nano Materials and Devices of Ministry of Education, and Institute for Frontier Science, Nanjing University of Aeronautics and Astronautics, Nanjing 210013, China

[3]School of Materials Science and Engineering, Nanyang Technological University, Singapore 639798, Singapore

[4]Environmental Chemistry and Materials Centre, Nanyang Environment and Water Research Institute, Nanyang Technological University, Singapore 637141, Singapore

[5]These authors contributed equally

*Correspondence: zhujuntong@ucas.ac.cn (J.Z.); z.liu@ntu.edu.sg (Z.L.); chuwazhang@nuaa.edu.cn (Z.Z.); wuzhou@ucas.ac.cn (W.Z.)

Received: May 25, 2023; Accepted: August 21, 2023; Published Online: August 22, 2023; https://doi.org/10.1016/j.xinn.2023.100502

© 2023 The Authors. This is an open access article under the CC BY license (http://creativecommons.org/licenses/by/4.0/).

Citation: Zhu J., Hu Z., Guo S., et al., (2023). Non-epitaxial growth of highly oriented transition metal dichalcogenides with density-controlled twin boundaries. The Innovation **4(6)**, 100502.

Twin boundaries (TBs) in transition metal dichalcogenides (TMDs) constitute distinctive one-dimensional electronic systems, exhibiting intriguing physical and chemical properties that have garnered significant attention in the fields of quantum physics and electrocatalysis. However, the controlled manipulation of TBs in terms of density and specific atomic configurations remains a formidable challenge. In this study, we present a non-epitaxial growth approach that enables the controlled and large-scale fabrication of homogeneous catalytically active TBs in monolayer TMDs on arbitrary substrates. Notably, the density achieved using this strategy is six times higher than that observed in convention chemical vapor deposition (CVD)-grown samples. Through rigorous experimental analysis and multigrain Wulff construction simulations, we elucidate the role of regulating the metal source diffusion process, which serves as the key factor for inducing the self-oriented growth of TMD grains and the formation of unified TBs. Furthermore, we demonstrate that this novel growth mode can be readily incorporated into the conventional CVD growth method by making a simple modification of the growth temperature profile, thereby offering a universal approach for engineering of grain boundaries in two-dimensional materials.

INTRODUCTION

Transition metal dichalcogenides (TMDs) have garnered significant attention due to their fascinating physicochemical properties and emerging applications.[1–3] Defect engineering, involving the introduction of specific point defects (eg, vacancies[4] or dopants[5]) or one-dimensional defects (e.g., grain boundaries [GBs], domain boundaries,[6,7] or edges[8]) into the two-dimensional (2D) TMD crystal lattices, plays a crucial role in enhancing and diversifying the intrinsic properties of 2D materials. For instance, catalytic activity for hydrogen evolution can be enhanced by introducing strained sulfur vacancies into the MoS_2 basal planes,[9] and the electrical transport properties of MoS_2 can be tuned by GBs with varying misorientation angles.[10] Owing to the diverse atomic configurations adopted by GBs in TMDs, contingent upon the misorientation angle, GB engineering emerges as an exceptionally versatile means to manipulate material properties.[11] Compared to small-angle GBs with sparsely separated 5|7 dislocation cores (i.e., pentagon-heptagon pairs) or 4|6 membered rings (i.e., tetragon-hexagon pairs),[4] 60° GBs in TMDs, commonly referred to as twin boundaries (TBs), exhibit a distinct atomic configuration, primarily comprising strings of four-membered rings separated by individual eight-membered rings.[4,12–14] These TBs serve as remarkable one-dimensional electron systems, showcasing notable features such as quantum well states,[15] charge density wave ordering,[16] and Tomonaga-Luttinger liquid behavior.[17] Furthermore, the incorporation of eight-membered rings into the TBs, forming 4|8 membered pairs, significantly enhances the catalytic activity for the hydrogen evolution reaction (HER).[6,18] Consequently, the controlled and large-scale fabrication of TBs, featuring designated atomic structure and controlled density, in monolayer TMDs holds crucial importance for fully exploring their potential in electronic and electrocatalytic applications.

Numerous approaches have been developed to construct and control TBs in TMDs.[19,20] For instance, TBs can be generated by inducing Se deficiency through thermal annealing[19] or incorporating excess Mo atoms into the lattice during molecular beam epitaxial (MBE) growth on lattice-matched substrates.[20] Both approaches result in the creation of high-density inversion domains in $MoSe_2$ surrounded by TBs. However, the size of such inversion domains is typically limited to a few nanometers, leading to the formation of short segments of TBs. This limitation in size may cause experimentally measured properties to deviate from the intrinsic properties of TBs.[21] Moreover, the TB segments originated from structural inversion in MBE-grown samples consist exclusively of four-membered rings. On the other hand, the chemical vapor deposition (CVD) growth method, utilizing van der Waals epitaxy on single-crystalline sapphire or graphene substrates,[22] can produce much longer TBs, typically on the order of micrometers, through the coalescence of 60° rotated grains. These TBs often contain a higher proportion of eight-membered rings,[12] a structural feature that benefits catalytic applications. However, the density of TBs in such CVD-grown samples is constrained by the low density of nucleation sites, resulting in poor tunability.

Herein, we propose a novel non-epitaxial growth approach to produce uniform TBs in TMDs with adjustable density on arbitrary substrates, including amorphous SiO_2, glass, single-crystalline sapphire, and mica. During the nucleation phase, a high density of small precursor particles diffuses and re-deposits surrounding larger precursor mounds at low temperatures, acting as nucleation sites. In the subsequent growth stage, the significantly different diffusivities of metal feedstocks on the bare substrate and the as-formed TMD surface limit the growth and size of the satellite TMD grains evolved from the surrounding tiny particles. This phenomenon facilitates the self-orientation of these grains as they merge with the larger central grains evolved from the precursor mounds. By adjusting the growth temperature, we can effectively control the densities of nuclei and TBs, enabling large-scale TB-density engineering. This growth mechanism is well supported by experimental analysis of intermediate products and multigrain Wulff construction simulations with a newly developed phenomenological method. Furthermore, we demonstrate that the self-oriented growth can be achieved by incorporating a precipitation phase into the temperature profile in the conventional CVD method. The resulting TBs in monolayer $MoSe_2$ obtained from this new growth strategy contain highly reactive eight-membered rings, leading to substantially improved HER performance.

RESULTS AND DISCUSSION

Synthesis and characterization of TB-rich $MoSe_2$

Figure 1A illustrates the growth process schematically (see section "material and methods" and Figure S1 for details). Initially, the Mo precursor dissolved in KOH solution is spin coated onto the SiO_2/Si substrate, leading to the formation of discrete and sparsely distributed mounds as the solution dries (Figure S2). When the growth temperature reaches 660°C, the Mo precursors diffuse into numerous Mo-containing particles, serving as nucleation sites for the growth of $MoSe_2$ (Figure S3), which contrasts the vaporization and redeposition of metal sources in conventional CVD methods.[23] Finally, $MoSe_2$ flakes grow from the central mounds and the surrounding particles, seamlessly fusing into larger $MoSe_2$ flakes with fuzzy hexagonal or Star-of-David shapes at 740°C (Figures 1B and S4). Atomic force microscopy (AFM) measurements (Figure 1C) confirm that the resulting flakes are monolayers. Additionally, the characteristic Raman peak of the A_{1g} mode is observed at 238 cm^{-1} (Figure 1D), and the photoluminescence (PL) peak of the A exciton is found at 1.54 eV (Figure 1E), both verifying the formation of monolayer crystalline $MoSe_2$. Raman mapping of the A_{1g}

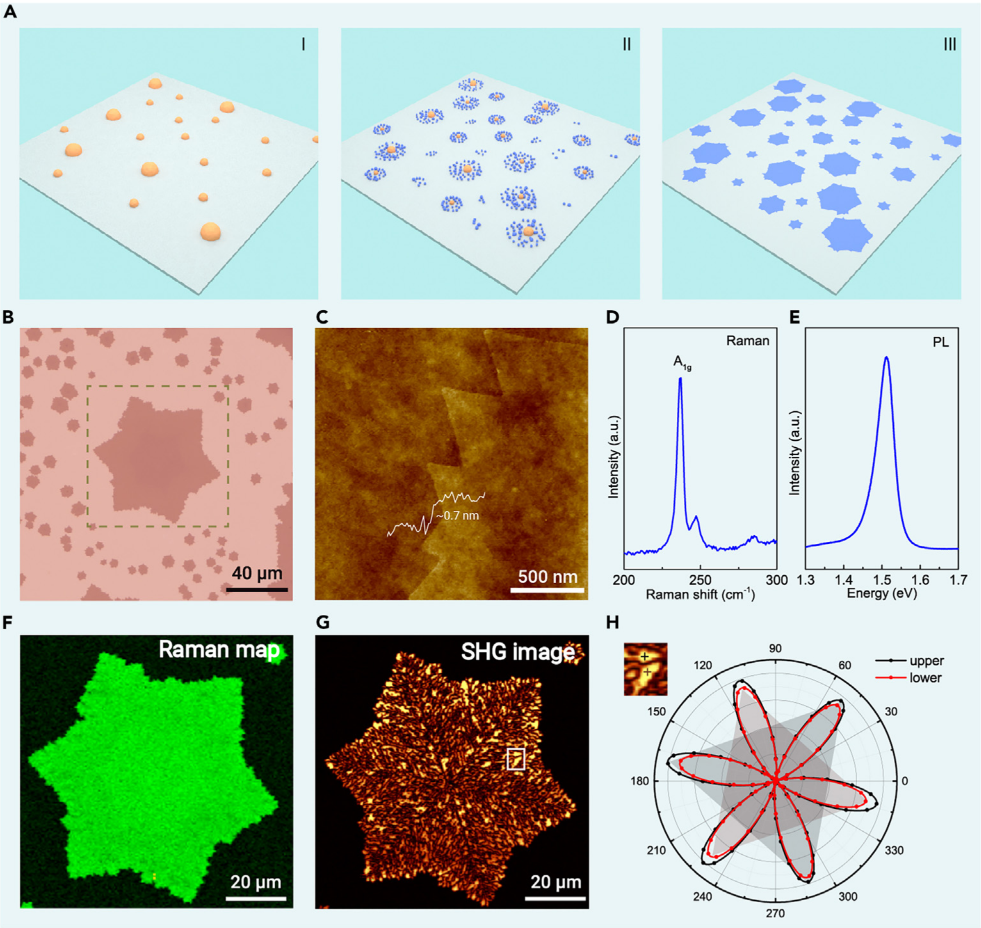

Figure 1. Growth and characterization of $MoSe_2$ with a high density of TBs (A) Schematic of the growth process. (i) Mo precursor (yellow mounds) is spin coated onto the substrate; (ii) small Mo-containing particles (blue spheres) diffuse out from the central precursor mounds at 660°C; (iii) fuzzy hexagonal or Star-of-David-shaped $MoSe_2$ flakes (blue flakes) grow at 740°C. (B) Optical image of a $MoSe_2$ flake grown on the SiO_2/Si substrate. (C) AFM image of the as-grown $MoSe_2$ flake with jagged edges. (D and E) Raman (D) and PL (E) spectra of the as-grown $MoSe_2$ monolayer. (F and G) Raman intensity mapping (F) and polarized SHG mapping (G) of the fuzzy Star-of-David-shaped $MoSe_2$ flake highlighted in (B). The Raman intensity mapping of the A_{1g} peak exhibits uniform contrast, while the SHG mapping contains a high density of low-contrast lines. (H) Polarization-resolved SHG spectra of adjacent domains as marked by the white frame in (G). The direction of each intensity maximum points to the armchair direction.

mode (Figure 1F) shows homogeneous spectroscopic quality in the as-formed $MoSe_2$ monolayer. Notably, unlike typical exfoliated single crystals (Figure S5B), the as-grown $MoSe_2$ monolayers exhibit jagged edges and an extra defect-related Raman peak at 247 cm^{-1} (Figure 1D).[24]

We utilized polarized second harmonic generation (SHG) mapping to investigate the presence of GBs in the as-grown $MoSe_2$ at the macroscopic scale. As depicted in Figure 1G, the pervasive low-contrast lines observed within the monolayer $MoSe_2$ flakes indicate a high density of GBs,[25] which can account for the sharp defect-related Raman peak observed in Figure 1D. To identify the tilt angle of these GBs, we collected polarization-resolved SHG spectra from adjacent grains, where the directions of the intensity maxima of the 6-fold anisotropic polarization pattern point to the armchair directions of the $MoSe_2$ lattice. As demonstrated in Figure 1H, the polarization patterns from the two adjacent grains exhibit the same armchair directions, possibly with a relative rotation of 60°. This, in conjunction with the clear presence of a GB between the two grains (inset of Figure 1H), implies that the two grains are misoriented by 60° and separated by a TB. Moreover, the polarization-resolved SHG images display similar levels of intensity inside each $MoSe_2$ flake (Figure S6), in contrast to the substantial intensity difference observed between flakes with non-60° relative rotations, suggesting that the GBs (the low-contrast lines in the SHG images) within individual $MoSe_2$ flakes are uniformly TBs.

Atomic structural analysis

Transmission electron microscopy (TEM) was employed to analyze the distribution of TBs in the monolayer $MoSe_2$ flakes. Figure 2A presents a TEM bright-field (BF) image of the flakes with jagged edges. The corresponding selected area electron diffraction (SAED) pattern shows only one set of diffraction spots, indicating the absence of non-60° GBs. To reveal the distribution of twin crystal domains, dark-field (DF) TEM imaging was performed by selecting one of the first-order diffraction spots to form the diffraction-contrast image. As shown in Figure 2B, distinct domains with alternating contrast are clearly visible across the entire $MoSe_2$ flake, arising from the 3-fold symmetry of the $MoSe_2$ lattice and the 60° rotation between adjacent twin domains. Typically, the twin domains show a trapezoidal shape with a length-to-width ratio of approximately 3 (Figure 2C), which differs from the equilateral triangular $MoSe_2$ inversion domains induced by Se deficiency.[19] The density of TB is estimated to be 5.1 μm/μm^2 (see details in Note S1 and Figure S7), which is 25 times higher than that observed in typical Star-of-David-shaped MoS_2 flakes (0.2 μm/μm^2) grown by the CVD method on SiO_2/Si substrates[12] and six times higher than that in the highly oriented MoS_2 film (0.8 μm/μm^2) grown on sapphire.[22]

The atomic configuration of the TBs was elucidated by aberration-corrected scanning transmission electron microscopy (STEM) high-angle annular dark-field (HAADF) imaging. As depicted by the yellow dashed lines in Figure 2D, a TB comprising multiple segments is highlighted, while the crystallographic orientations of the two adjacent domains are denoted by the violet triangles. The magnified HAADF-STEM image (Figure 2E) provides a direct visualization of the atomic arrangements along the TB, characterized by a distinctive alternation of four- and eight-membered rings, exhibiting a molybdenum-rich structure (a structural model is shown in Figure 2F). The relative populations of eight- and four-membered rings within the TBs are summarized in Figure 2G, based on an analysis performed across 10 randomly selected regions (Figure S8). The proportion of eight-membered rings within our sample is approximately 40%, a figure that significantly exceeds those previously reported within literature.[4,12] The incorporation of a substantial proportion of reactive eight-membered rings within the TBs is anticipated to introduce a dramatic enhancement in the electrocatalytic activity of the as-grown TB-rich monolayer $MoSe_2$. Further discussion on this aspect will be provided in the subsequent sections.

Non-epitaxial growth mechanism

To investigate the mechanism underlying the formation of high-density TBs in $MoSe_2$, we conducted a thorough examination of intermediate products at various growth temperatures. This was accomplished in conjunction with the execution of phenomenological simulations and density functional theory (DFT) calculations. Given the comprehensive information gathered, we propose a self-oriented nucleation and growth mechanism as the genesis of high-density TBs in monolayer TMDs.

Thermogravimetric analysis (TGA) (Figure 3A) demonstrates that the sublimation or decomposition of the Mo precursor is negligible within the temperature range of 500°C and 740°C. Within the temperature bracket of 660°C and 740°C, Mo precursors, reportedly in the form of Mo_3O_9 molecules, predominantly diffuse from the precursor mounds as surface adsorbates,[26,27] functioning as the primary source of Mo feedstock. Notably, the presence of circular clouds composed of minuscule particles surrounding the large Mo precursor mounds is observed in the intermediate sample obtained at 660°C (Figures 3D$_1$, S3, and

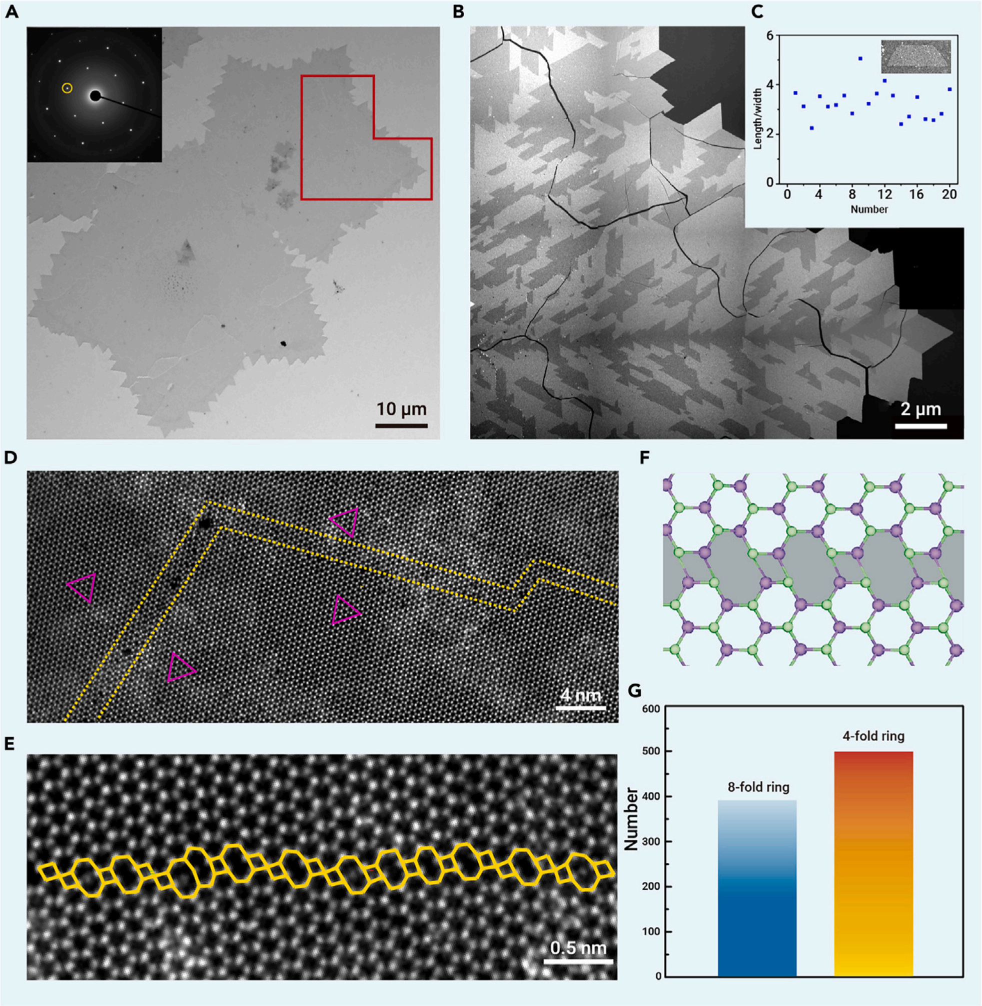

Figure 2. Distribution and atomic structure of the high-density TBs in $MoSe_2$ (A) Low-magnification BF-TEM image of $MoSe_2$ flakes. Inset: SAED pattern acquired from the upper-right corner of the flake. (B) DF-TEM image of the selected region highlighted by the red lines in (A). The image was stitched by multiple DF-TEM images at higher magnification. The black lines inside the $MoSe_2$ flake are cracks formed during the sample transfer. (C) Statistics of the aspect ratio of the twin domains. Inset shows the DF-TEM image of a trapezoid twin domain. (D) HAADF-STEM image of a segmented TB. The violet triangles illustrate the inversed lattice orientations on both sides of the TB, and the yellow dashed lines depict the TB region. (E) High-resolution HAADF-STEM image of a TB in $MoSe_2$. Alternating four- and eight-membered rings are marked by yellow tetragons and octagons. (F) The top view of a TB model with consecutive four- and eight-membered rings. The green and purple balls represent the Se and Mo atoms, respectively. (G) Statistics for eight- and four-membered rings in TBs from analysis of 10 different sample regions.

S10). This observation appears to challenge the established concept of Ostwald ripening, whereby larger particles tend to evolve through consumption of their smaller counterparts. This discrepancy suggests that the diffused Mo_3O_9 molecules have interacted with the surrounding Se to form MoO_xSe_y alloys (Figure S10). These alloys, possessing higher melting points, are inclined to deposit more readily on the substrate. Conversely, when the identical precursor was annealed at 660°C without an Se atmosphere, such minuscule particle clouds were absent (Figure S11).

Following the formation of particle clouds, both the central Mo precursor mounds and the minuscule particles in their vicinity function as nucleation sites for the subsequent growth of $MoSe_2$ grains. Apart from the local feedstock present at these nucleation sites, the Mo precursor mounds also act as the principal suppliers of Mo feedstock through surface diffusion, given the negligible source of vapor-phase metal at lower temperatures. DFT calculations reveal that the primary form of Mo feedstock (i.e., Mo_3O_9) diffuses 10 orders of magnitude faster on the newly formed $MoSe_2$ surfaces than on the bare SiO_2 substrate (Figure 3B; Table S1). Owing to this significant discrepancy in diffusion rates, isolated $MoSe_2$ grains distant from the Mo precursor mounds struggle to secure a sufficient supply of Mo feedstock, resulting in their slow growth. Consequently, a sizable central $MoSe_2$ "continent" emerges from each Mo precursor mound, while only small $MoSe_2$ "islands" evolve from the surrounding tiny MoO_xSe_y particles, adopting random orientations due to the absence of van der Waals epitaxy. As the growth proceeds, the central $MoSe_2$ continent expands, gradually incorporating the surrounding $MoSe_2$ islands. Importantly, upon contacting with the large $MoSe_2$ continents, the small $MoSe_2$ islands undergo reorientation to align with the lattice of the large domains. This alignment is achieved via capillary forces,[28] generating a seamless multigrain flake (Figure 3C) that exhibits either 0° or 60° misorientation crossing all GBs. We refer to this non-epitaxial growth model as the self-oriented growth mode.

Within the self-oriented growth mode, the disparity in diffusivity of Mo_3O_9 on SiO_2 and $MoSe_2$ surfaces, as well as the growth rate of $MoSe_2$, significantly influences the morphological evolution of the growth. As temperature escalates, the growth rate surges at a rate much more pronounced than the diffusion rate of feedstocks (see the details in Note S2). Therefore, we primarily examine the impact of temperature on the growth rate in the subsequent discussion. At low temperatures (≤670°C), the expansion of the central $MoSe_2$ continent is comparatively sluggish, permitting a small amount of Mo feedstock to reach offshore $MoSe_2$ nucleation sites through surface diffusion. This results in the formation of a cloud composed of either small MoO_xSe_y particles or diminutive $MoSe_2$ grains (Figure $3D_1$). Due to the isotropic diffusion of Mo feedstock on SiO_2, this cloud is circular and ultimately evolves into a loosely packed rounded rim of $MoSe_2$ as the growth continues (Figures $3E_1$ and S12). Upon reaching a temperature of 680°C, the $MoSe_2$ continent consumes the Mo feedstock at an accelerated rate, causing even slower growth of the cloud, which consequently results in a densely packed rounded $MoSe_2$ rim (Figures $3F_1$ and S13). At elevated temperatures of 710°C and 740°C, the $MoSe_2$ continent nearly depletes all available Mo feedstock, culminating in a fuzzy Star-of-David shape (see details in Note S3; Figures $3G_1$ and S14).

Our theory regarding the self-oriented nucleation and growth of multiple-twinned crystals is further supported by phenomenological simulations utilizing a custom multigrain Wulff construction algorithm (see section "material and methods" for details). In this context, we employ the kinetic constant *k* of growth fronts of $MoSe_2$ grains as a descriptor to represent the influence of the growth temperature via an Arrhenius equation (see details in Note S2),[29] with a higher *k* corresponding to a higher temperature. As depicted in Figures $3D_2$–$3G_2$, an increase in *k* triggers a morphological evolution in the sample. It transforms from a central $MoSe_2$ continent accompanied by a circular cloud of diminutive surrounding $MoSe_2$ grains (Figure $3D_2$) to a loosely packed rounded $MoSe_2$ rim (Figure $3E_2$), and subsequently to a compact rounded $MoSe_2$ rim (Figure $3F_2$). In an extreme scenario, where the difference between mobilities of feedstock on the substrate surfaces and $MoSe_2$ is virtually infinite and the Mo feedstock is in excess, the large-scale simulation (Figure $3G_2$) reproduces almost identically the morphology and distribution of twin crystals observed experimentally in Figure 2B. This outcome is in line with our previous predictions.

TB engineering and HER performance

The aforementioned discussion implies that, at low growth temperatures, the diffusion and redeposition of metal source play a critical role in facilitating the self-oriented nucleation and growth of TMDs with a high density of TBs

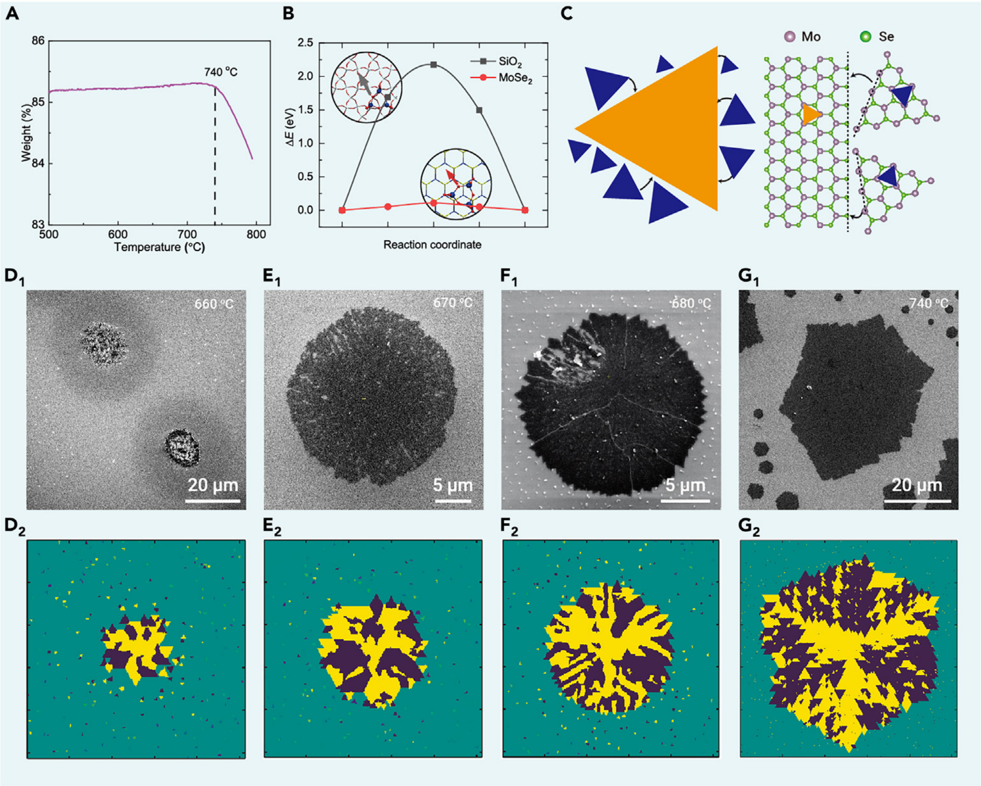

Figure 3. Self-oriented growth of $MoSe_2$ with high-density TBs (A) TGA measurement of the Mo precursor. The weight is normalized to the total weight of the Mo precursors containing $(NH_4)_6Mo_7O_{24}\cdot 4H_2O$ and KOH at room temperature (Figure S9). The TGA result indicates that the state of the precursor and the growth mode of $MoSe_2$ should differ dramatically below and above 740°C. (B) The calculated diffusion energy barriers of Mo_3O_9 on the surfaces of SiO_2 and $MoSe_2$, respectively. The diffusion barrier of Mo_3O_9 on the surface of SiO_2 is much higher than that on the surface of $MoSe_2$. (C) Schematic of the formation of TBs via self-oriented nucleation and growth. (D_1–G_1) SEM images of samples grown at 660°C, 670°C, 680°C, and 740°C, respectively. The morphology of the products transforms from a large mound to an incomplete rim and finally to a fuzzy Star-of-David-shaped flake. (D_2–G_2) Phenomenological simulations of $MoSe_2$ samples in D_1–G_1. The fractal landlocked distribution and length/height ratio of trapezoidal twinned domains in G_2 are similar to the experimental results revealed by DF-TEM imaging.

(Figure 4Ai). Conversely, when the growth temperature exceeds 740°C, the sublimation of the Mo precursor becomes substantial (Figure 3A), and the formation of surrounding MoO_xSe_y particles via diffusion and deposition is significantly reduced. This results in a marked decrease in the number of domains and GBs within each $MoSe_2$ flake (Figure 4Aii). Correspondingly, the high-temperature growth of $MoSe_2$ flakes is predominantly guided by edge-epitaxial growth using a vapor-phase metal source (Figure S15). This sharply contrasts with the self-oriented growth observed at lower temperatures, which operates via surface diffusion and the annexation of surrounding smaller islands.

The temperature-dependent competition between these two distinct growth mechanisms suggests that the density of TBs can be engineered simply through the careful modulation of the growth temperature. The SHG images in Figures 4B_2–D_2 indeed manifest the decrease of TB density in $MoSe_2$ samples as the growth temperature rises from 740°C to 770°C and finally 800°C. In addition, the transition from the self-oriented growth to edge-epitaxial growth, concurrent with rising temperature, likely reduces the availability of Mo feedstock due to the rapid depletion of the Mo mounds. Consequently, the monolayer flake edges evolve from Mo-terminating zigzag edges to Se-terminating edges, accompanying an increase in Se chemical potential (see the details in Note S4 and Figure S16). This transformation reshapes the flakes from a Star-of-David shape (740°C; Figure 4B_1) into a hexagon (800°C; Figure 4D_1). Notably, the edge regions of these samples exhibit lower TB densities, primarily due to the surrounding grains, which serve as nuclei for the subsequent growth of $MoSe_2$, are denser near the central mound, and become sparser as they move away from the central mound (Figure S3). This variation in nucleus distribution influences the TB density in the edge regions. Additionally, the edge epitaxy assumes a dominant role during the high-temperature growth (>740°C), contributing further to the lower grain density in the edge region. Consequently, the TBs in the edge regions of hexagonal-shaped $MoSe_2$ flakes are particularly scarce (Figure 4D_1). Remarkably, this self-oriented nucleation and growth strategy exhibits insensitivity toward the growth substrate (Table S1). We demonstrate that $MoSe_2$, with high-density TBs, can also be successfully grown on amorphous glass and single-crystalline sapphire and mica (Figure S17). Furthermore, this approach can be extended to the growth of other TMD materials, such as WSe_2 (Figure S18), thus serving as a universal methodology for TB engineering in 2D TMD materials.

Given the high proportion of the catalytically active eight-membered rings in our TBs (Figure 2G), we have adopted a single-entity on-chip micro-electrochemical cell (Figure S19) to assess the HER performance of the above-mentioned monolayer $MoSe_2$ samples with varying TB densities. For a systematic comparison, we examined monolayer $MoSe_2$ samples grown under 740°C, 770°C, and 800°C, displaying an averaged TB density of 5.1, 3.2, and 1 $\mu m/\mu m^2$, respectively. These results were compared with a $MoSe_2$ monolayer sample grown using the conventional CVD method, which exhibited negligible TBs (~0.2 $\mu m/\mu m^2$ in density). To exclude the potential impacts from the Au electrode[30] and edges of $MoSe_2$,[18] the as-prepared materials were first transferred onto HER-inert graphene electrodes, and then reaction windows were precisely defined on the basal plane of the $MoSe_2$ devices, while the rest of the sample area was protected by poly(methylmethacrylate) (PMMA) films (~1 μm thick) (Figure 4F). Interestingly, we discover that the presence of TBs with eight-membered rings can remarkably boost the HER activity of $MoSe_2$, as shown by the escalating HER current density in line with increasing TB density in these samples. Significantly, the 740°C-grown $MoSe_2$, possessing ultra-high-density TBs, demonstrated an approximately 100-times increase in current density at −300 mV vs. reversible hydrogen electrode (RHE) when compared to that in the conventional CVD-grown $MoSe_2$. The potential influence of Se vacancies[9,31] on the observed catalytic trend can be excluded by the similar Se vacancy concentrations across the samples (Figure S20). We also conducted electrochemical stability tests of 740°C-grown $MoSe_2$. Figure S21 demonstrates that the TB-rich $MoSe_2$ samples exhibit decent stability, maintaining their performance over a period of 15 h at a current density of 10 mA cm^{-2}. Together, these results suggest that the key catalytic contributors are TBs with eight-membered rings, while the contributions from Se vacancies and the pristine $MoSe_2$ basal plane are negligible. This observation is fully consistent with previous *ab initio* calculations.[13,32] It is worth noting that the monolayer $MoSe_2$ prepared at 740°C showed the best HER activity with an overpotential of −195 mV at a current density of 10 A cm^{-2} (Figure 4G) and a Tafel slope of 70 mV dec^{-1} (Figure 4H), which are superior to previously reported GB-rich monolayer TMD materials (Table S2).

Extending non-epitaxial TB growth to conventional CVD methods

In conventional CVD growth methods that use solid-state metal sources (Figure 5A), such as metal powders,[33] metal oxides,[34] and pure TMD powders,[35] the metal precursor powders typically undergo a vaporization-redeposition process. Therefore, if we add a precipitation stage to the temperature profile prior to TMD growth, the vaporized metal source could pre-deposit onto the substrate, generating large feedstock mounds similar to those observed in our OH-assisted CVD method discussed above and introducing high density of TBs through the self-oriented growth mode. We test this hypothesis using molten-salt-assisted CVD

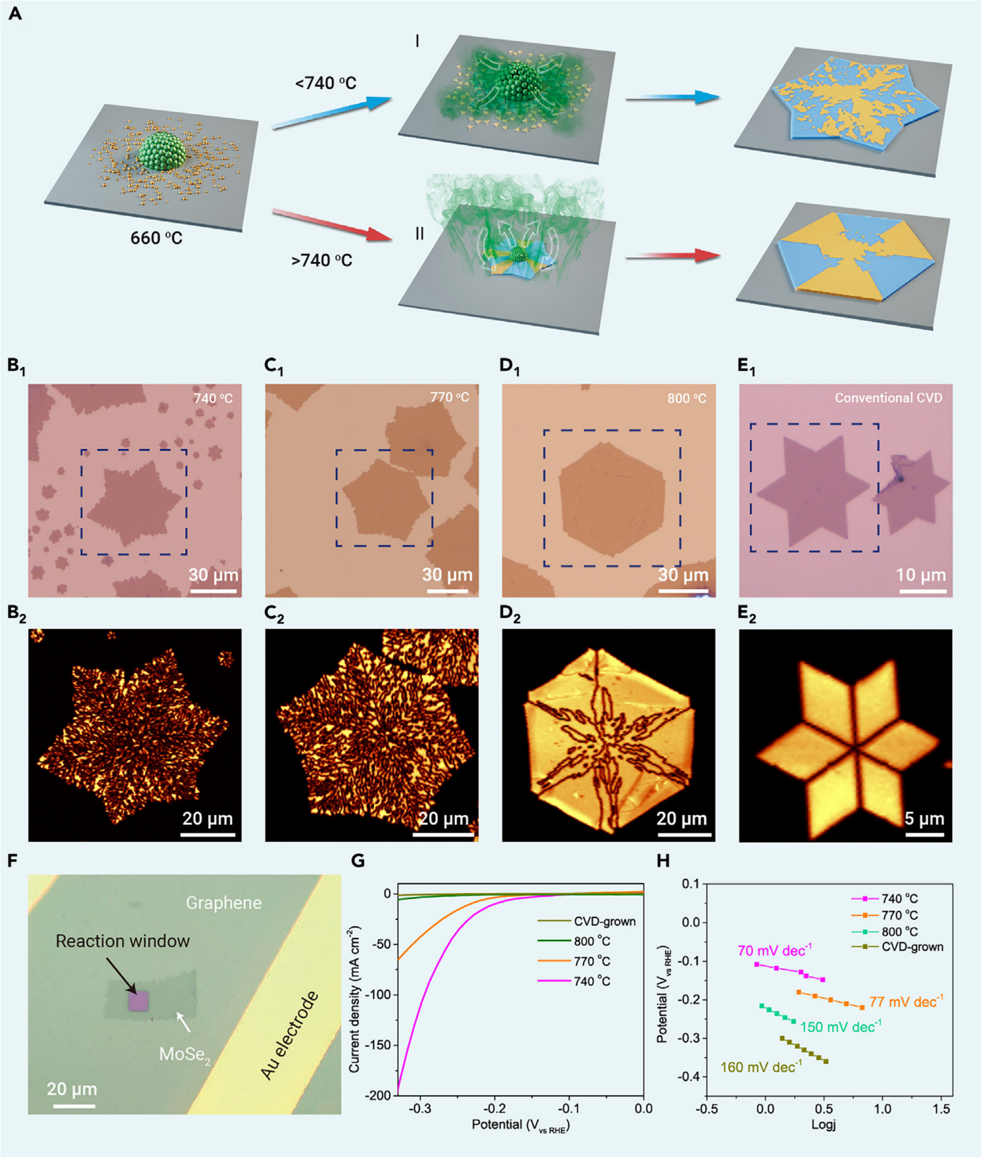

Figure 4. TB-density engineering in $MoSe_2$ monolayers and HER performance (A) Schematic of the self-oriented growth and the edge-epitaxial growth modes at different temperatures. (i) Below 740°C, where the gas-phase feedstock is negligible, the diffusion and redeposition of Mo precursors from the central mounds onto the substrate surface produces many $MoSe_2$ islands, and the expansion of the central $MoSe_2$ "continent" is accompanied by the annexation of surrounding islands under self-oriented growth. In contrast, (ii) above 740°C, the gas-phase feedstock dominates, resulting in fewer islands on the substrate, and the expansion of the continent is dominated by epitaxial growth along the edges. The green arrows represent the migration of Mo sources. (B–D) Optical (B_1–D_1) and SHG (B_2–D_2) images of monolayer $MoSe_2$ grown at 740°C, 770°C, and 800°C for 3 min, respectively, using the OH-assisted CVD method. (E) Optical (E_1) and SHG (E_2) images of $MoSe_2$ grown at 740°C by a conventional molten-salt-assisted CVD method without precipitation stage. The as-grown $MoSe_2$ flake contains only six TBs. (F) Optical micrograph of a $MoSe_2$ micro-device. The micro-device includes a PMMA reaction window on the $MoSe_2$ basal plane and graphene and Au electrodes. (G and H) Polarization curves of the current density (G) and the corresponding Tafel plots (H) of the micro-devices for the basal planes of 740°C, 770°C, and 800°C-grown and CVD-grown $MoSe_2$, respectively.

growth.[23] A precipitation stage at 650°C was added to the temperature profile, which is below the $MoSe_2$ growth temperature (Figure 5B; see more experimental details in section "material and methods"). This stage enables the deposition of large MoO_x particles onto the substrate surface (Figure S22). During the subsequent temperature ramp from 660°C to 740°C, the metal feedstock diffusing out from the large MoO_x particles generates a high density of nucleation sites, which then evolve into small $MoSe_2$ domains and merge with the central continents following the self-oriented nucleation and growth (Figure 4Ai). This modified CVD route produces $MoSe_2$ monolayers with high-density TBs (Figures 5C and 5D), providing strong evidence for our theory. In contrast, the molten-salt-assisted CVD growth, when performed without the precipitation stage, mainly produces Star-of-David $MoSe_2$ with only six TBs (Figures 4E_1 and S23).

Based on the results presented, we propose that surface diffusion and redeposition of metal precursors cannot be completely eliminated in CVD growth. As such, the self-oriented nucleation and growth mechanism should come into play to different extents in all CVD growth. The observed evolution of TB density and morphology of TMD at different temperatures (Figures 4B_2–4D_2) offers guidance for growing monolayer TMDs for specific applications. For instance, for electronic device applications where high-quality single crystals are preferred, conditions of low growth temperature and high metal flux conditions may not be ideal due to the dominance of the self-oriented nucleation and growth mechanism. On the contrary, elevated growth temperatures that inhibit the pre-deposition of metal precursors facilitates the edge-epitaxial growth of high-quality single crystals, which can also explain the use of ultra-high temperatures (over 950°C) for wafer-scale TMD single-crystal growth.[36–38]

CONCLUSION

We have unveiled and demonstrated a non-epitaxial growth strategy that introduces highly reactive TBs in $MoSe_2$ and WSe_2 monolayers through a self-oriented growth mechanism. The synthesized TB-rich $MoSe_2$ monolayers featuring plentiful eight-membered rings indeed exhibit impressive electrocatalytic performance. The mechanism is built on the surface diffusion and redeposition of metal sources at moderate temperatures, as well as the significant difference in the diffusivities of metal feedstocks on the surfaces of substrate and the newly formed TMD. We demonstrate that both self-oriented growth and the conventional edge-epitaxial growth modes are prevalent in various CVD-based methods. Therefore, the density and atomic structure of TBs can be engineered by modulating the competition between these two growth modes through adjusting the growth temperature. Overall, our study paves the way to large-scale GB engineering of TMD monolayers via CVD growth and provides a promising platform to explore novel quantum states in 1D electronic systems and their potential applications in electronics and electrocatalysis.

MATERIAL AND METHODS

Please refer to the supplemental information for details on methods.

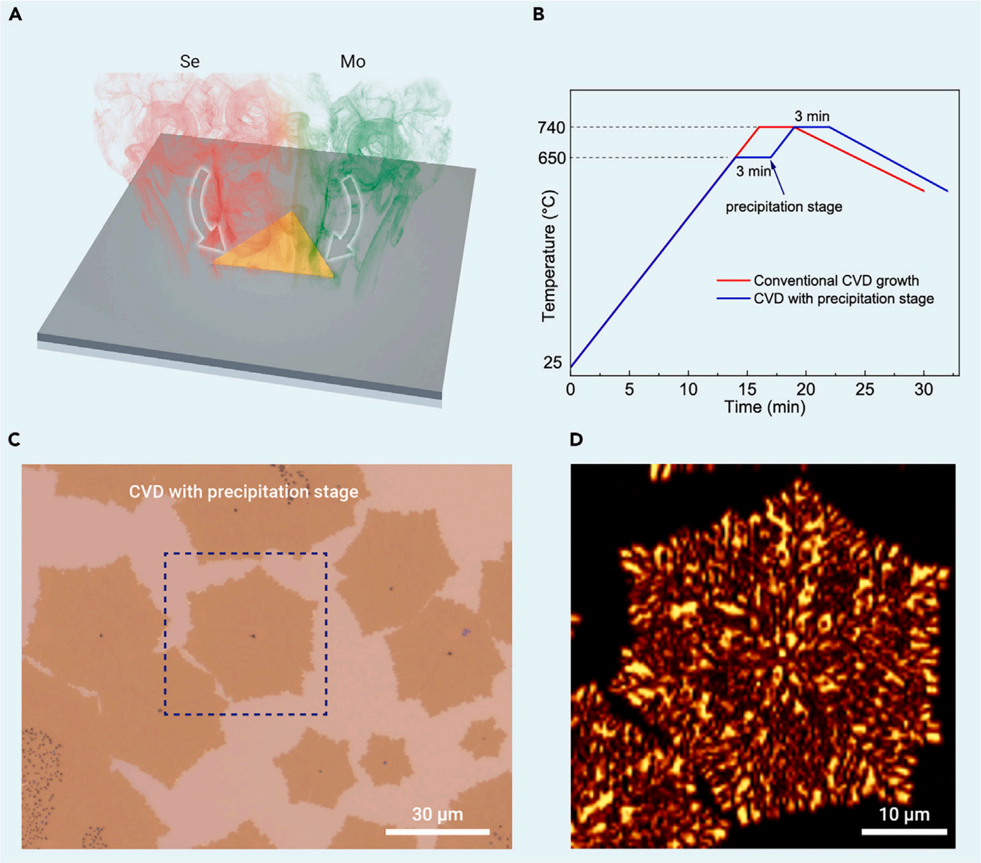

Figure 5. Growth of high-density TBs by a modified CVD method (A) Schematic of conventional CVD growth method using vaporized metal source. (B) Temperature profiles for a conventional CVD growth process (red) and a modified CVD process with a precipitation stage at 650°C (blue). (C and D) Optical (C) and SHG (D) images of $MoSe_2$ grown at 740°C using the modified CVD method with a precipitation stage, showing a high density of TBs.

REFERENCES

1. Wang, Q.H., Kalantar-Zadeh, K., Kis, A., et al. (2012). Electronics and optoelectronics of two-dimensional transition metal dichalcogenides. Nat. Nanotechnol. ***7***, 699–712.
2. Manzeli, S., Ovchinnikov, D., Pasquier, D., et al. (2017). 2D transition metal dichalcogenides. Nat. Rev. Mater. ***2***, 17033.
3. Zou, X., Xu, Y., and Duan, W. (2021). 2D materials: rising star for future applications. Innovation ***2***, 100115.
4. Zhou, W., Zou, X., Najmaei, S., et al. (2013). Intrinsic structural defects in monolayer molybdenum disulfide. Nano Lett. ***13***, 2615–2622.
5. Zhou, J., Lin, J., Sims, H., et al. (2020). Synthesis of Co-doped MoS_2 monolayers with enhanced valley splitting. Adv. Mater. ***32***, 1906536.
6. Zhu, J., Wang, Z.-C., Dai, H., et al. (2019). Boundary activated hydrogen evolution reaction on monolayer MoS_2. Nat. Commun. ***10***, 1348.
7. Lin, J., Fang, W., Zhou, W., et al. (2013). AC/AB stacking boundaries in bilayer graphene. Nano Lett. ***13***, 3262–3268.
8. Zhao, X., Fu, D., Ding, Z., et al. (2018). Mo-terminated edge reconstructions in nanoporous molybdenum disulfide film. Nano Lett. ***18***, 482–490.
9. Li, H., Tsai, C., Koh, A.L., et al. (2016). Activating and optimizing MoS_2 basal planes for hydrogen evolution through the formation of strained sulphur vacancies. Nat. Mater. ***15***, 48–53.
10. Ly, T.H., Perello, D.J., Zhao, J., et al. (2016). Misorientation-angle-dependent electrical transport across molybdenum disulfide grain boundaries. Nat. Commun. ***7***, 10426.
11. Man, P., Srolovitz, D., Zhao, J., et al. (2021). Functional grain boundaries in two-dimensional transition-metal dichalcogenides. Acc. Chem. Res. ***54***, 4191–4202.
12. van der Zande, A.M., Huang, P.Y., Chenet, D.A., et al. (2013). Grains and grain boundaries in highly crystalline monolayer molybdenum disulphide. Nat. Mater. ***12***, 554–561.
13. Yu, M., Zhu, C., He, Y., et al. (2021). Polymorphism of segmented grain boundaries in two-dimensional transition metal dichalcogenides. Nano Lett. ***21***, 6014–6021.
14. Batzill, M. (2018). Mirror twin grain boundaries in molybdenum dichalcogenides. J. Phys. Condens. Matter ***30***, 493001.
15. Xia, Y., Zhang, J., Jin, Y., et al. (2020). Charge density modulation and the Luttinger liquid state in $MoSe_2$ mirror twin boundaries. ACS Nano ***14***, 10716–10722.
16. Barja, S., Wickenburg, S., Liu, Z.-F., et al. (2016). Charge density wave order in 1D mirror twin boundaries of single-layer $MoSe_2$. Nat. Phys. ***12***, 751–756.
17. Ma, Y., Diaz, H.C., Avila, J., et al. (2017). Angle resolved photoemission spectroscopy reveals spin charge separation in metallic $MoSe_2$ grain boundary. Nat. Commun. ***8***, 14231.
18. Ouyang, Y., Ling, C., Chen, Q., et al. (2016). Activating inert basal planes of MoS_2 for hydrogen evolution reaction through the formation of different intrinsic defects. Chem. Mater. ***28***, 4390–4396.
19. Lin, J., Pantelides, S.T., and Zhou, W. (2015). Vacancy-induced formation and growth of inversion domains in transition-metal dichalcogenide monolayer. ACS Nano ***9***, 5189–5197.
20. Liu, H., Jiao, L., Yang, F., et al. (2014). Dense network of one-dimensional midgap metallic modes in monolayer $MoSe_2$ and their spatial undulations. Phys. Rev. Lett. ***113***, 066105.
21. Jolie, W., Murray, C., Weiß, P.S., et al. (2019). Tomonaga-Luttinger liquid in a box: electrons confined within MoS_2 mirror-twin boundaries. Phys. Rev. X ***9***, 011055.
22. Yu, H., Liao, M., Zhao, W., et al. (2017). Wafer-scale growth and transfer of highly-oriented monolayer MoS_2 continuous films. ACS Nano ***11***, 12001–12007.
23. Zhou, J., Lin, J., Huang, X., et al. (2018). A library of atomically thin metal chalcogenides. Nature ***556***, 355–359.
24. Zhao, S., Lu, M., Xue, S., et al. (2019). A Se vacancy induced localized Raman mode in two-dimensional MoSe2 grown by CVD. Preprint at arXiv.
25. Yin, X., Ye, Z., Chenet, D.A., et al. (2014). Edge nonlinear optics on a MoS_2 atomic monolayer. Science ***344***, 488–490.
26. Karma, A., and Plapp, M. (1998). Spiral surface growth without desorption. Phys. Rev. Lett. ***81***, 4444–4447.
27. Zhu, J., Xu, H., Zou, G., et al. (2019). MoS_2–OH bilayer-mediated growth of inch-sized monolayer MoS_2 on arbitrary substrates. J. Am. Chem. Soc. ***141***, 5392–5401.
28. Artyukhov, V.I., Hu, Z., Zhang, Z., et al. (2016). Topochemistry of bowtie- and star-shaped metal dichalcogenide nanoisland formation. Nano Lett. ***16***, 3696–3702.
29. Meca, E., Lowengrub, J., Kim, H., et al. (2013). Epitaxial graphene growth and shape dynamics on copper: phase-field modeling and experiments. Nano Lett. ***13***, 5692–5697.
30. Shi, Y., Wang, J., Wang, C., et al. (2015). Hot electron of Au nanorods activates the electrocatalysis of hydrogen evolution on MoS_2 nanosheets. J. Am. Chem. Soc. ***137***, 7365–7370.
31. Xia, B., Wang, T., Jiang, X., et al. (2018). Ar^{2+} beam irradiation-induced multivancancies in $MoSe_2$ nanosheet for enhanced electrochemical hydrogen evolution. ACS Energy Lett. ***3***, 2167–2172.
32. Zhu, C., Yu, M., Zhou, J., et al. (2020). Strain-driven growth of ultra-long two-dimensional nano-channels. Nat. Commun. ***11***, 772.
33. Gong, Y., Lin, J., Wang, X., et al. (2014). Vertical and in-plane heterostructures from WS_2/MoS_2 monolayers. Nat. Mater. ***13***, 1135–1142.
34. Wang, X., Gong, Y., Shi, G., et al. (2014). Chemical vapor deposition growth of crystalline monolayer $MoSe_2$. ACS Nano ***8***, 5125–5131.
35. Yang, T., Zheng, B., Wang, Z., et al. (2017). Van der Waals epitaxial growth and optoelectronics of large-scale WSe_2/SnS_2 vertical bilayer p–n junctions. Nat. Commun. ***8***, 1906.
36. Liu, L., Li, T., Ma, L., et al. (2022). Uniform nucleation and epitaxy of bilayer molybdenum disulfide on sapphire. Nature ***605***, 69–75.
37. Wang, J., Xu, X., Cheng, T., et al. (2022). Dual-coupling-guided epitaxial growth of wafer-scale single-crystal WS_2 monolayer on vicinal a-plane sapphire. Nat. Nanotechnol. ***17***, 33–38.
38. Liu, F., Chen, K., and Xue, D. (2023). How to fast grow large-size crystals? Innovation ***4***, 100458.

ACKNOWLEDGMENTS

We thank Liangzhuo Ma for help with the TGA measurements. This work was supported by the National Key R&D Program of China (2018YFA0305800), Natural Science Foundation of

China (51872285), the Beijing Outstanding Young Scientist Program (BJJWZYJH01201914430039), CAS Project for Young Scientists in Basic Research (YSBR-003), and the Fundamental Research Funds for the Central Universities. The work at NUAA was supported by the National Key R&D Program of China (2019YFA0705400) and Natural Science Foundation of China (1221101035, 12225205, 22073048), and the computations were in part performed at the High-performance Computational Center at NUAA. This work was also supported in part by the Singapore Ministry of Education AcRF Tier 2 (MOE2019-T2-2-105 and MOE-MOET2EP10121-0006) and AcRF Tier 1 (RG7/21).

AUTHOR CONTRIBUTIONS

W.Z. and J.Z. designed the project. J.Z performed the sample synthesis and structural characterization under the supervision of W.Z. Z.H. performed the simulations and analyzed the simulation results. M.Y. and M.X. performed the first-principles calculations. Z.Z. supervised the theoretical part. S.G. carried out the HER studies under the supervision of Z.L. A.L. contributed to the analysis of TEM/STEM data. J.Z. and W.Z. wrote the paper with inputs from Z.H., R.L., J.P., and S.J.P. All authors contributed to the discussion of the results and editing of the manuscript.

DECLARATION OF INTERESTS

The authors declare no competing interests.

SUPPLEMENTAL INFORMATION

It can be found online at https://doi.org/10.1016/j.xinn.2023.100502.

LEAD CONTACT WEBSITE

http://zhouwu.ucas.ac.cn

The Innovation, Volume *4*

Supplemental Information

Non-epitaxial growth of highly oriented transition metal dichalcogenides with density-controlled twin boundaries

Juntong Zhu, Zhili Hu, Shasha Guo, Ruichun Luo, Maolin Yu, Ang Li, Jingbo Pang, Minmin Xue, Stephen J. Pennycook, Zheng Liu, Zhuhua Zhang, and Wu Zhou

Methods

Engineering TBs in $MoSe_2$ and WSe_2 monolayers by OH-assisted CVD method.

We first dissolved 45 mg of ammonium molybdate tetrahydrate and 128 mg of KOH or NaOH in 10 ml of deionized water, forming Mo precursor, OH^- ions were used to guarantee the monolayer nature of the grown TMD[1]. The Mo precursor was then spin-coated onto a clean SiO_2/Si substrate (300 nm thick SiO_2 over Si) at 8000 rpm. Mo precursor-covered substrate was put into tube furnace, the details of the growth process and parameters are depicted in **Supplemental Figure 1**, the carrier gas is 5% Ar/H_2. TB engineered $MoSe_2$ monolayers were grown at 740, 770 and 800 °C for 3 minutes. The synthesis recipe for WSe_2 is similar to that of $MoSe_2$, where the sodium tungstate, sapphire and 10% Ar/H_2 are used as the W source, growth substrate and carrier gas, respectively. We found that the Mo precursors grown at 650 °C remain in their original state without any diffusion of Mo nucleation site (**Supplemental Figure 2c**). When the growth temperature rises to 660 °C, high density Mo nucleation sites start to diffuse out from the central mound (**Supplemental Figure 3**). Therefore, the investigated intermediate products were grown at 660, 670, 680, 710 and 740 °C (**Figure 3D-G**), being cooled directly after reaching the target temperature without maintaining a constant temperature. All the reactions were carried out under atmospheric pressure.

Growth of $MoSe_2$ monolayers by molten-salt-assisted CVD method.

A powder mixture of 3 mg MoO_3 and 0.5 mg NaCl in an aluminium oxide boat was placed in the centre of the quartz tube. We added a precipitation stage to the

temperature profile prior to TMDs growth at 650 °C, which exceeds the melting point of the MoO_3 and NaCl mixture, ensuring that sufficient metal source is pre-deposited onto the substrate to activate self-oriented growth. The typical Star-of-David $MoSe_2$ (**Figure 4E**) was produced at 740 °C without the precipitation stage at 650 °C.

TEM and STEM sample preparation.

We used a poly (methyl methacrylate) (PMMA) assisted method to prepare STEM samples. A thin layer of PMMA (Allersist, AR-P 672.045) film was spin coated onto the SiO_2/Si substrate and then baked at 85 °C for 10 min. The PMMA-covered substrate was then immersed in 2M NaOH solution for corroding SiO_2 layers of the substrate. The PMMA/sample film was immersed in deionized water and washed after the separation from the substrate. After that, the film was fished out by a Cu TEM grid (Quantifoil, 50 nm Cu foil of 200 mesh). Finally, the PMMA support layer was dissolved by soaking the grid in acetone at 80 °C for 2 minutes.

Material characterization.

The morphology was characterized by SEM (FEI Helios G4) and Raman spectroscopy (Alpha 300RS+, WITec). Raman and PL spectroscopy was performed with a 532nm laser whose power is ~1.5 mW at room temperature (Alpha 300RS+, WITec). SHG mapping was obtained under excitation from a 1064 nm laser with an average power of 20 mW (Rainbow 1064 OEM). AFM images were obtained using a Bruker Dimension Icon AFM system. Bright and dark-field TEM characterization was

carried out on JEOL 2100Plus TEM with an accelerating voltage of 80 kV. STEM measurements were performed on an aberration-corrected Nion U-HERMES100 microscope operating at 60 kV. The probe-forming semi-angle is 32 mrad, and the collection half angle for HAADF imaging is between 75 and 210 mrad. The beam current was about 3 pA for a single shot image. TGA measurements were produced on TGA 50.

Multi-grain Wulff construction algorithm.

The phase-field method can simulate multi-grain shapes such as the David star shape of MoS_2 flakes[27], but is not employed here due to its low efficiency under the situation where hundreds of $MoSe_2$ grains are involved in a single simulation. Instead, we use a self-developed multi-grain Wulff construction algorithm, which is more efficient to predict the growth morphology of multi-grain systems without growth instabilities (thus the growth shape of any standalone single grains is simply their Wulff shape). For the ease of description, the single-grain Wulff shape is designated to be the equilibrium shape of TMDs, *i.e.* triangle, in the following discussion.

In the algorithm, N grains coexist in a 2D canvas. Each grain has a fixed center. An order parameter φ is used to represent the state of each pixel/point of the canvas, where $\varphi = 1$ means the coverage of grains and $\varphi = 0$ denotes the bare substrate. Initially each grain occupies a predefined area, or a nucleus. The jth edge of the ith grain moves away from the grain center according to,

$$\dot{l_{ij}} = k(\xi_{ij} - \xi_{eq}),$$

where l_{ij} is the distance between the edge and the grain center (*i.e.*, growth rate), k the kinetic constant, ξ the dimensionless supersaturation, ξ_{eq} the equilibrium value of ξ, and ξ_{ij} the mean ξ along the edge exposed to the bare substrate. At each pixel, the dimensionless supersaturation $\xi = c/c_s$, where c is the feedstock concentration, and c_s is the nominal concentration of precursor in solid 2D crystals. In each time step of the simulation, if a pixel of the canvas has $\varphi=0$ and is inside the enclosure of the three edge extension lines of grain i, where $i=1,2,\ldots,N$, and at least one of its neighboring pixels is already occupied by grain i, then this pixel belongs to grain i.

Meanwhile, the evolution of ξ needs to be solved by the equation,

$$\dot{\xi} = \nabla(D\nabla\xi) - \frac{\xi}{\tau_s} + F - \frac{\delta\varphi}{\delta t},$$

where F is the deposition flux, D is the diffusion coefficient of ξ, τ_s is the desorption time, and the last term denotes the rate of the change of φ.

Phenomenological simulation setups.

A 200×200 canvas is used in each simulation. Initially, a central grain is placed at the center pixel of the canvas and its orientation is fixed to 30° such that a tip always points upward (the north direction). This grain is also a permanent part of the central "continent". Except for the central grain, each satellite grain is initially assigned with a random grain orientation and occupies a random pixel. Once a satellite grain attaches to the "continent", it self-orients by rotation such that it differs from its neighboring grain by either 60° or 0°. The initial value of ξ is 0.1 everywhere. To mimic the effect of self-oriented growth, ξ within a distance of 5 pixels to the canvas center is set to a

value ξ_c, where $\xi_c = t$ when $t<50$, and $\xi_c=50$ otherwise. To exhibit the effect of a non-uniform diffusivity D, we set $D=100$ where $\varphi=1$, and $D=0.01$ where $\varphi=0$. We further simplify the simulation by presuming the small growth rates for all edges of each grain. Different k is tested to examine the effect of temperature. Other parameters in simulations include $N=200$, $F=0$, $\tau_s=1$, and $\xi_{eq}=0.1$. With a time step of 1 and a grid size of 1, the simulation is solved with the finite difference method.

First-principles calculations of diffusion coefficients.

First-principles calculations were performed using the Vienna Ab-initio Simulation Package (VASP) code[2], with the Perdew-Burke-Ernzerh (PBE) parametrization of the generalized gradient approximation (GGA) as the exchange correlation potential and projector-augmented wave (PAW) method for the core region. The kinetic energy cutoff was chosen to be 400 eV for the plane-wave expansion, and a vacuum region of 20 Å was set to avoid spurious interaction between adjacent slabs. Structures were fully relaxed until the force on each atom was less than 0.01 eV/Å. The Brillouin zone integration was sampled by a 5 × 5 × 1 k-grid mesh for the $MoSe_2$ and SiO_2 supercells with lattice constants of ~15 Å. The energy barriers of Mo_3O_9 migration were computed with the climbing image nudged elastic band (NEB) method.

According to the random walk model for diffusion, the diffusion coefficient, D_{H_a}, of activated Mo_3O_9 molecules can be computed as

$$D_{H_a} = \frac{va^2}{z},$$

where a is the traveling distance of Mo_3O_9 in a single hop, v denotes the microscopic

jump frequency, and z is the number of neighboring sites to which Mo_3O_9 can hop. According to the transition state theory, the microscopic jump frequency v is related to the activation energy E_{act} for surface diffusion, determined by the following equation

$$v = \frac{kT}{h}\exp(\frac{-E_{act}}{kT}),$$

where T is temperature, k the Boltzmann constant, and h the Planck constant.

Fabrication of micro-electrochemical device and electrocatalytic measurement.

First, we grew large-scale continuous graphene films on the Cu foils by the CVD method. Second, the as-grown graphene films were transferred onto pre-patterned chips using the PMMA-assisted method, then as-grown $MoSe_2$ monolayers were transferred on the graphene surface. Third, an annealing process at 200 °C under high-vacuum conditions (1×10^{-5} torr) was used to eliminate residual molecules between the graphene and MoS_2 films to obtain optimized interfaces. Fourth, e-beam lithography (EBL) was used to fabricate the electrodes (Cr/Au, 2 nm/60 nm) on graphene to connect the device. Fifth, a 500 nm-thick layer of PMMA film was spin-coated on the device chip, and then EBL was employed to remove the PMMA film from the interested region where six-point star or hexagonal $MoSe_2$ flakes exist, making sure it is the only exposed region of the active catalyst in the HER test.

The typical four-electrode micro-electrochemical measurements were conducted in a 0.5M H_2SO_4 electrolyte solution. The scan rate was set to be 5 mV per step. The electrocatalytic current and conductance current are simultaneously detected.

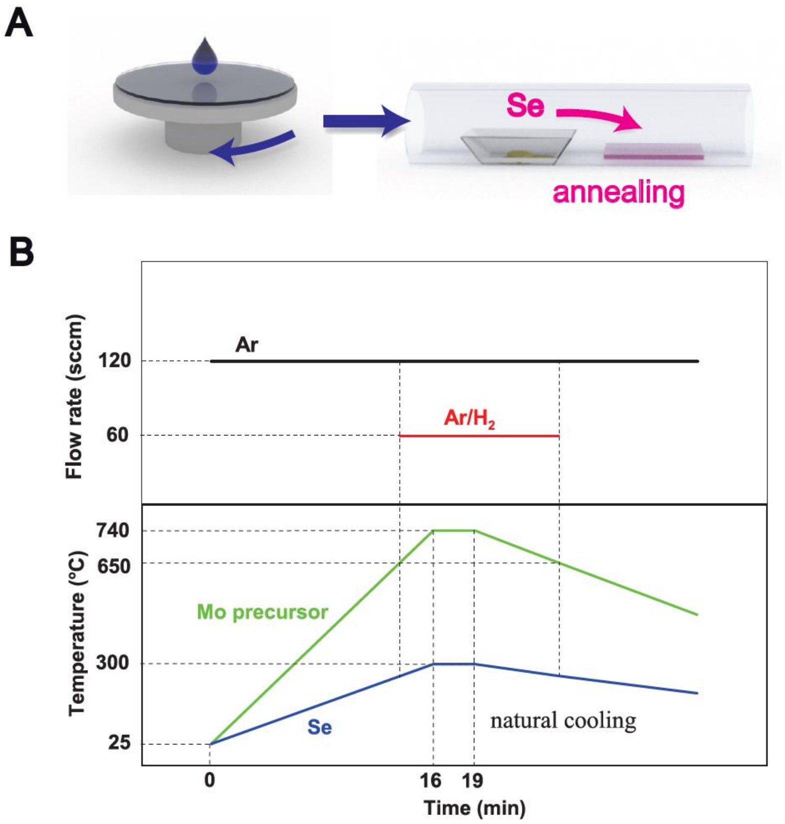

Figure S1 Parameters for the growth of $MoSe_2$ by OH-assisted CVD method. **(A)** Schematic diagram of OH-assisted CVD method, including spin-coating Mo precursor solution and annealing under Se atmosphere. **(B)** Detailed growth parameters of OH-assisted CVD method. When the temperature rises to 650 °C, Se powders partially evaporate into the ambient air. At the same time, the introduced reducing gas (H_2) starts to initiate the reaction between Se and MoO_x precursors. With the increase of temperature, the growth rate of $MoSe_2$ increases and eventually forms the highly crystalline monolayer $MoSe_2$.

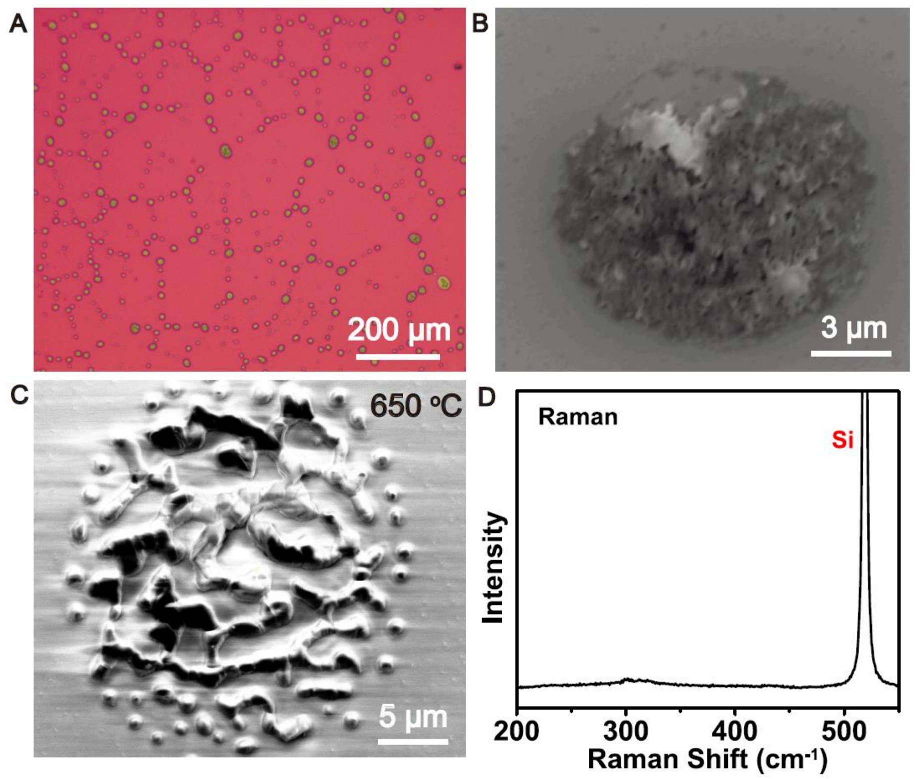

Figure S2 Morphology of Mo precursor after spin-coating and annealed at 650 °C. **(A and B)** Optical and SEM images of spin-coated Mo precursor. Due to the poor hydrophilicity of SiO_2/Si, the Mo precursor solute is deposited on the substrate surface in the form of large mounds immediately after evaporation of water. **(C)** SEM image of Mo precursor annealed at 650 °C, remaining in their original state without the diffusion of tiny Mo-contained particles. **(D)** Raman spectrum of 650 °C-grown samples absents the characteristic peaks of $MoSe_2$.

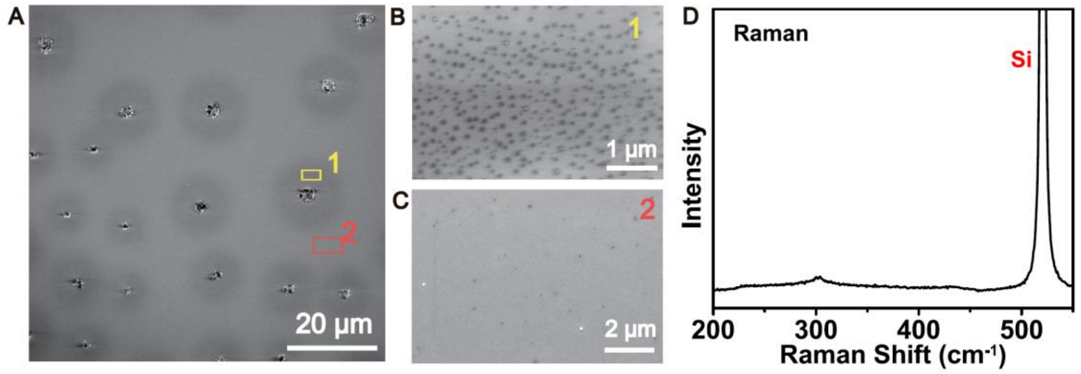

Figure S3 Morphology of Mo precursor annealed at 660 °C. **(A)** Low-magnification SEM image of Mo precursor annealed at 660 °C. **(B and C)** Magnified SEM image of region 1 and 2 in **A**. A high-density of Mo nucleation sites begins to diffuse out from central mounds at 660 °C. **(D)** Raman spectrum of the annealed Mo precursor indicates that the diffused particles have not formed $MoSe_2$ crystals.

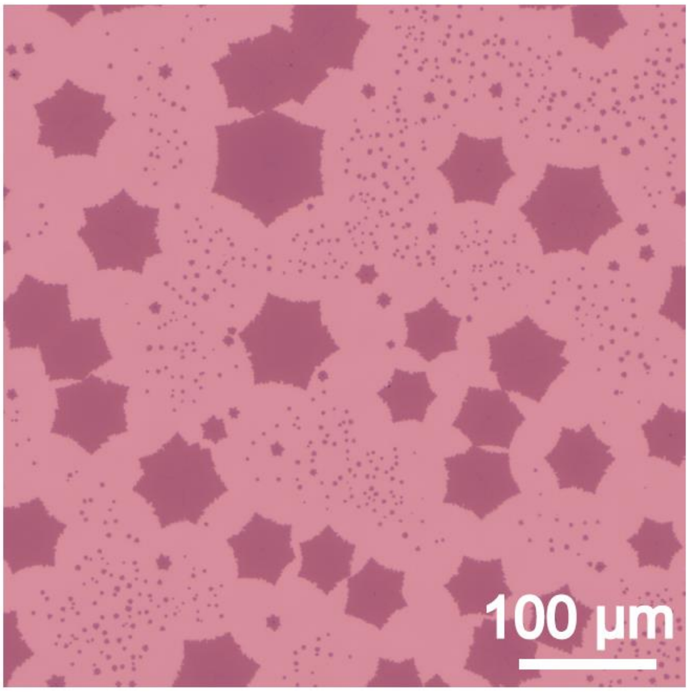

Figure S4 Optical image of the $MoSe_2$ grown at 740 °C for 3 min. The average transverse length of the fuzzy hexagonal $MoSe_2$ flake is about 50 µm.

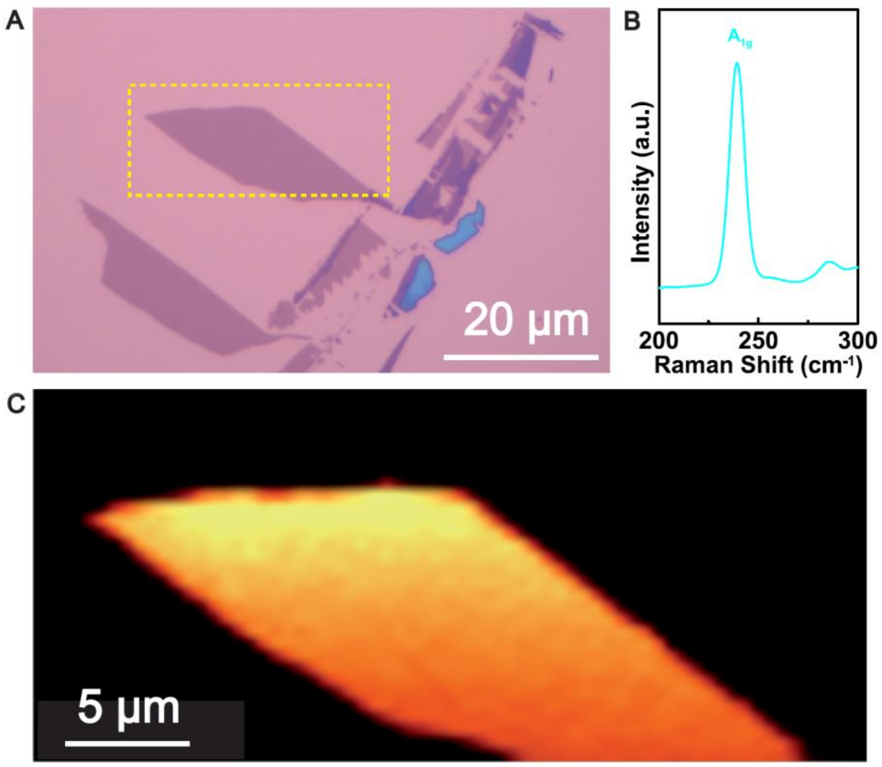

Figure S5 Morphology and spectroscopy of exfoliated $MoSe_2$ single-crystal. **(A)** Optical image of exfoliated $MoSe_2$ nanosheet. **(B)** Raman spectrum of exfoliated $MoSe_2$. **(C)** SHG image of exfoliated $MoSe_2$ in **A**. The uniform contrast indicates the single-crystal nature of the exfoliated $MoSe_2$.

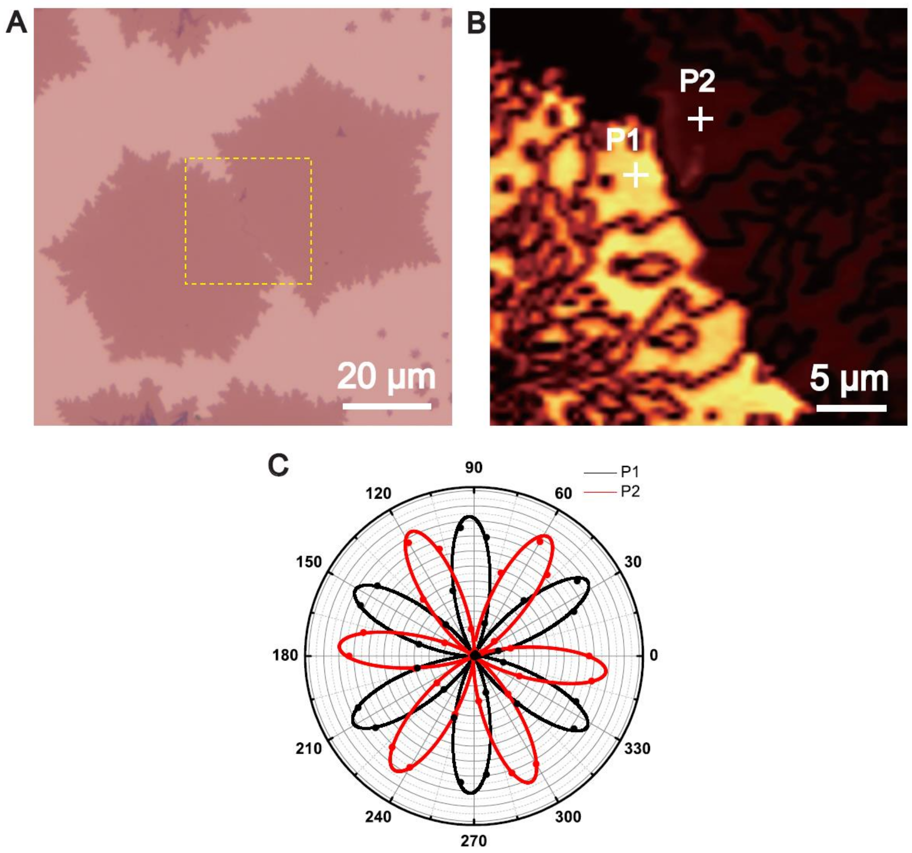

Figure S6 Spectroscopy of as-grown TB-rich $MoSe_2$. **(A)** Optical image of as-grown $MoSe_2$ monolayers. **(B)** Polarized SHG images of the adjacent $MoSe_2$ flakes. (**C**) Polarization-resolved SHG spectra of adjacent flakes marked by the white crosses in **B**. The difference in orientation angle between the two flakes is about 25º

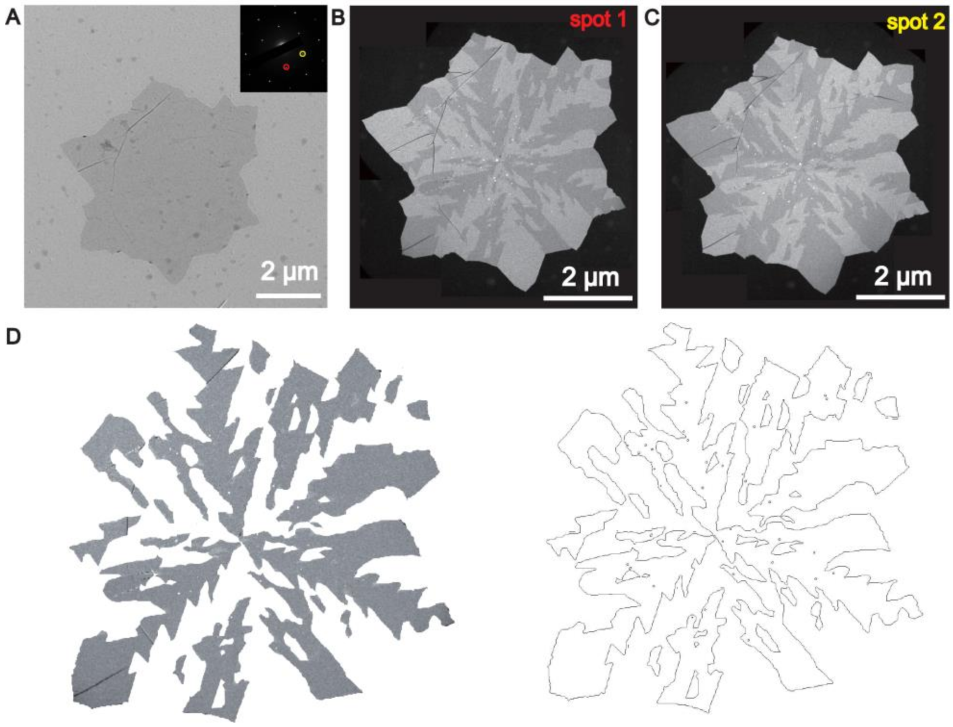

Figure S7 Structure of TB-rich $MoSe_2$ flake. **(A)** Bright-field image of 740 °C-grown $MoSe_2$ specimen, with diffraction pattern inseted. **(B and C)** Dark-field images collected from the selected diffraction spots in the inseted diffraction pattern of **A**. **B** and **C** show the opposite contrast, confirming their twinned structure. **(D)** Calculation of total length of TB in $MoSe_2$ (**A**).

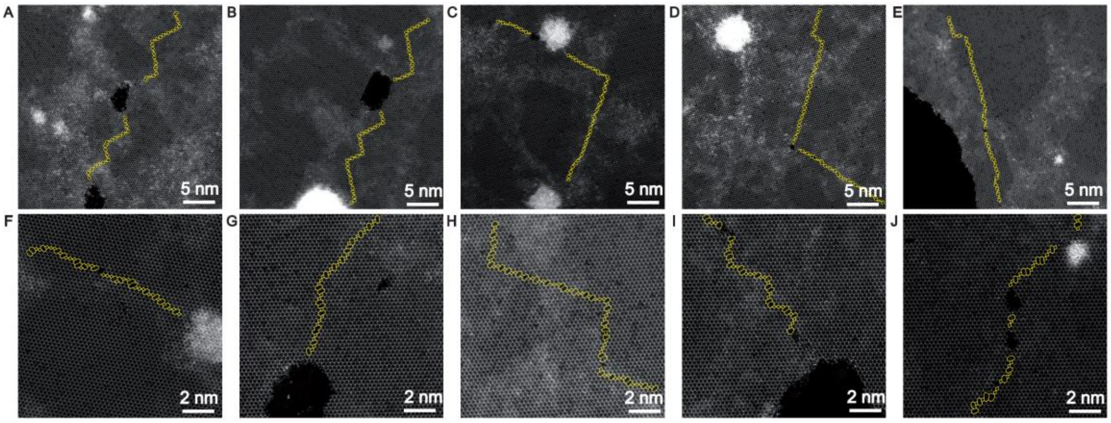

Figure S8 Microstructure of TBs in 10 different areas. **(A-J)** STEM images show the TBs with a high percentage of 8-membered rings.

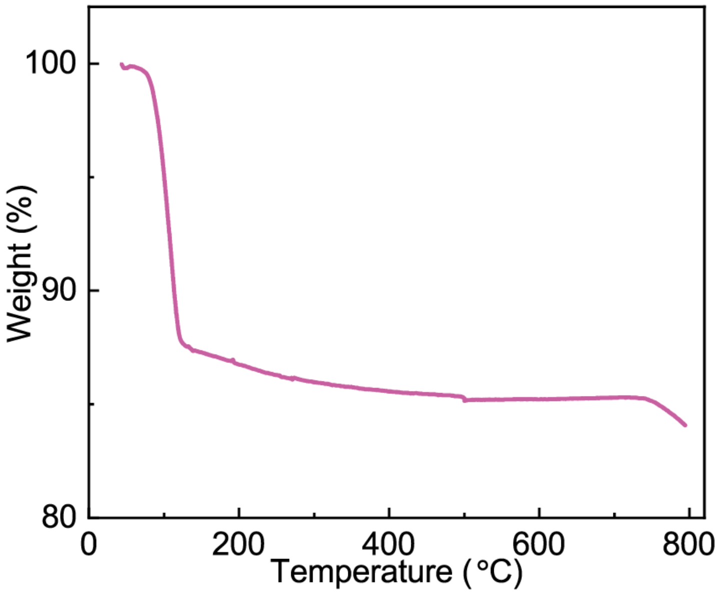

Figure S9 TGA curve of Mo precursor from room temperature to 800 °C.

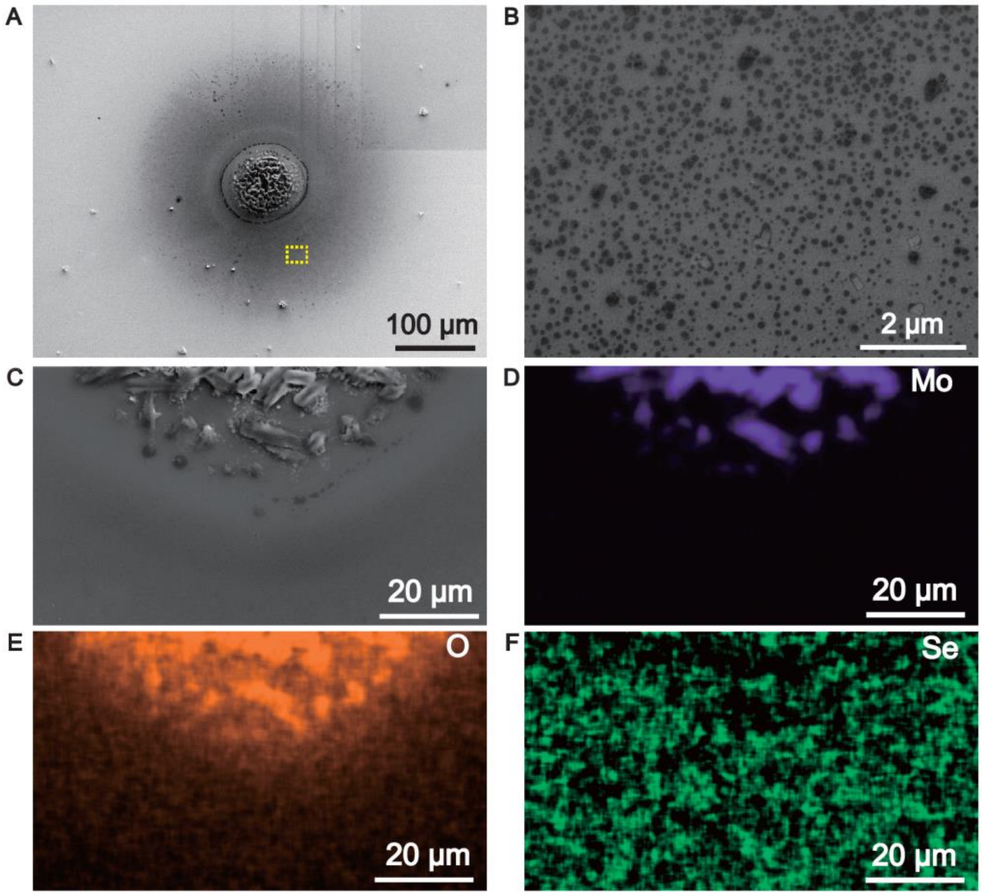

Figure S10 Structure of 660 °C-grown $MoSe_2$ on Si substrate. **(A)** Low-magnification SEM image of 660 °C-grown $MoSe_2$ on Si substrate. **(B)** The magnified SEM image of the marked region in **A** shows a high-density of tiny particles around the Mo precursor mound. **(C-F)** SEM image (**c**) and corresponding EDX maps of Mo (**D**), O (**E**) and Se (**F**) elements. The inner mound shows obvious Mo and O signals but a low Se signal intensity, indicating its MoO_x structure. Meanwhile, the Se signal intensity of the surrounding tiny particles is significantly stronger than that of the inner Mo precursor mound, confirming the selenization of diffused tiny Mo particles, which prevents the Ostwald ripening and generates a high density of diffused tiny particles. It is worth noting that, in order to exclude the influence of substrate on the distribution of O, the $MoSe_2$ is grown on Si substrate. The as-grown samples have the same morphology as those grown on SiO_2 substrate (**Figure S3**).

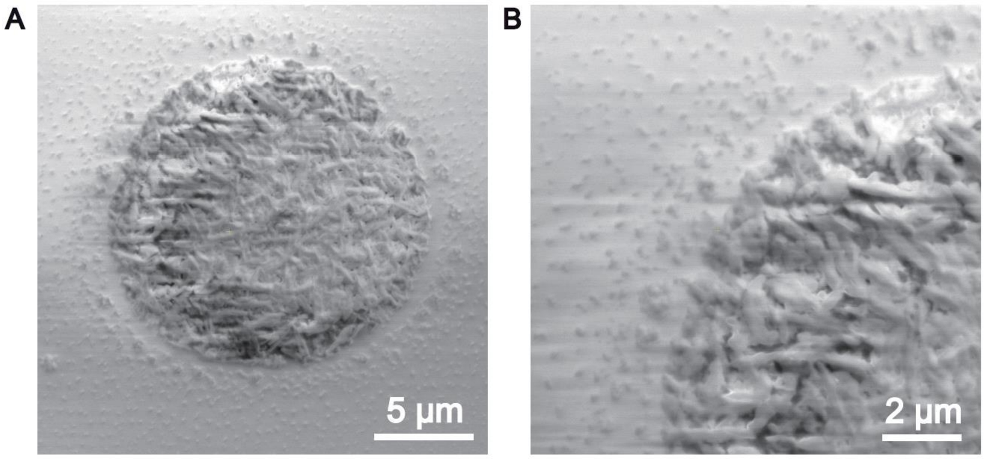

Figure S11 Structure of 660 °C-grown $MoSe_2$ without Se atmosphere. **(A)** Low-magnification SEM image of 660 °C-grown $MoSe_2$ on Si substrate. **(B)** The magnified SEM image shows sparse large-sized particles around the central mound and no dense tiny Mo feedstock.

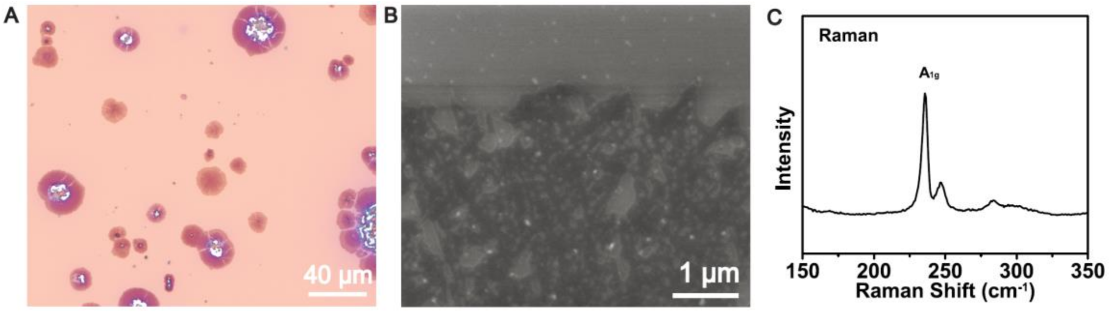

Figure S12 Structure of 670 °C-grown $MoSe_2$. **(A)** Low-magnification optical image of 670 °C-grown $MoSe_2$. Tiny grains grow around the large Mo precursor mounds. **(B)** Enlarged SEM image of circular $MoSe_2$. The loosely packed rounded rim of $MoSe_2$ contains many holes and tiny particles. **(C)** Raman spectrum of 670 °C-grown $MoSe_2$ with a typical peak at 239 cm^{-1}.

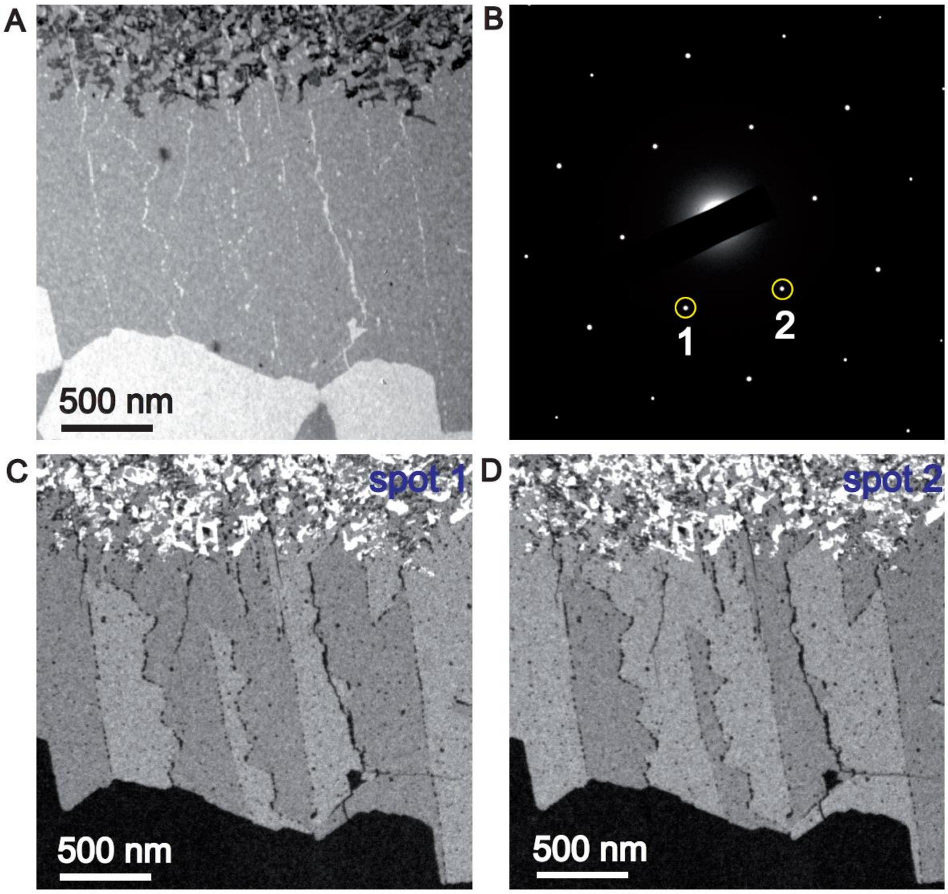

Figure S13 TEM images of 680 °C-grown $MoSe_2$. **(A)** Bright-field TEM image of as-grown $MoSe_2$ shows a relatively complete structure. **(B)** electron diffraction pattern of $MoSe_2$ in **A**. **(C and D)** Dark-field images collected from the selected diffraction spot 1 (**C**) and 2 (**D**) in **B**, respectively. The opposite contrast of **C** and **D** is consistent with their twin structure. The above results show that once the islands merge, TBs are formed.

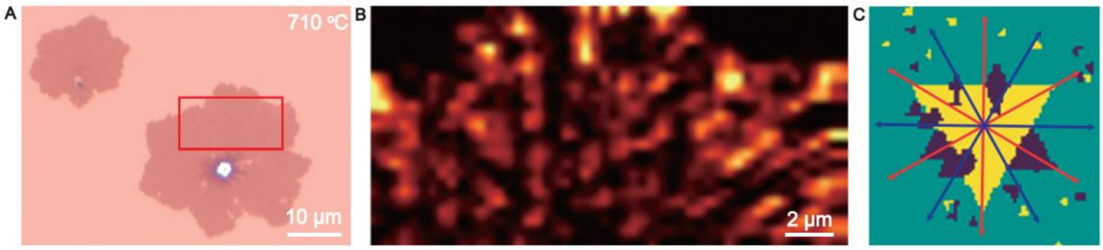

Figure S14 Structure of 710 °C-grown $MoSe_2$. **(A)** Optical image of an irregular shaped 710 °C-grown $MoSe_2$ with jagged edges, different from the rounded $MoSe_2$ grown at 680 °C. **(B)** SHG image of $MoSe_2$ grown at 710 °C shows high-density TBs and the twinned crystals show a nanoribbon shape. (**C**) Schematic diagram of a fuzzy Star-of-David shape appears.

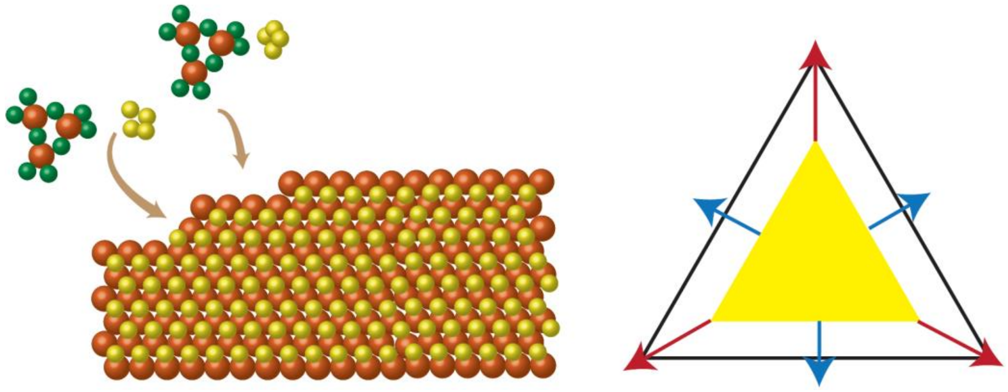

Figure S15 $MoSe_2$ grows epitaxially along the edges by vapor-phase metal sources. When the growth temperature is above the melting point of Mo precursor, vapor-phase Mo sources become the main source of Mo feedstock. The ratio of the growth rate along the three tip directions (red arrows) to the three edge directions (blue arrows) is 2:1.

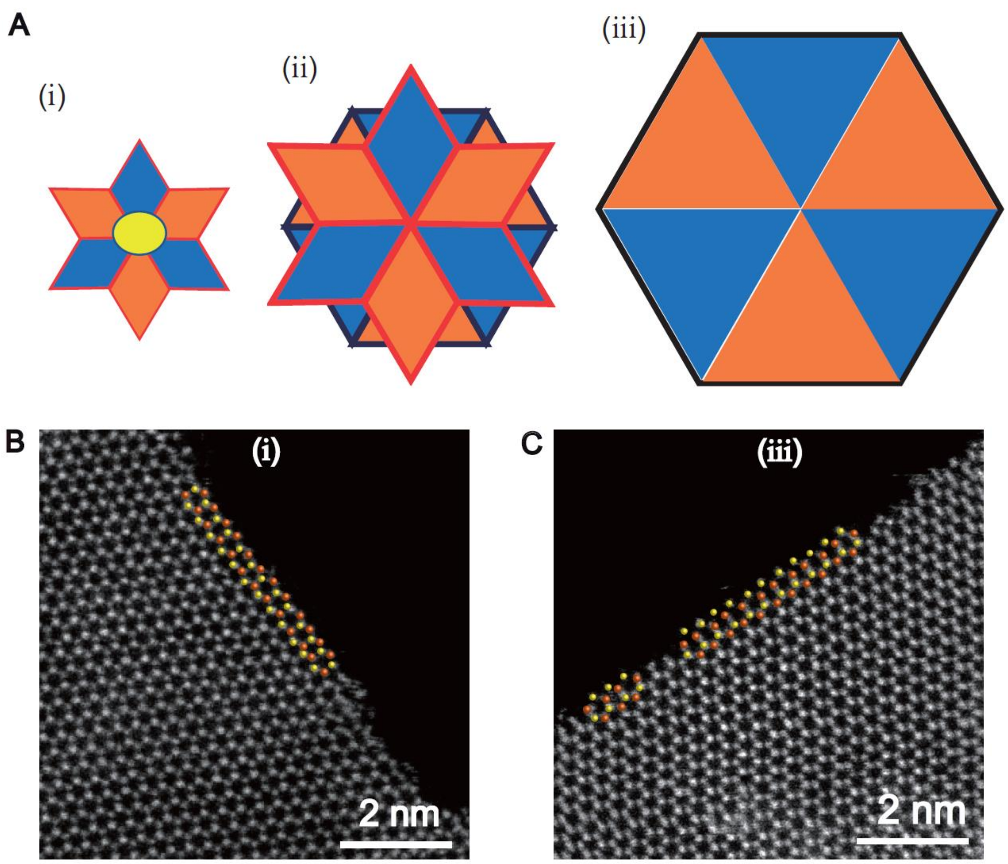

Figure S16 Edge termination of fuzzy David star (740 °C-grown sample) and hexagonal $MoSe_2$ (800 °C-grown sample). (A) (**i**) At a temperature below 740 °C, $MoSe_2$ self-oriented grows in a Mo-rich environment (large Mo precursor mounds provide a sufficient source of Mo) and forms a Mo termination as a matter of course; (**ii**) At higher growth temperatures above 740 °C, the mound is quickly exhausted, resulting in a reduction in the abundance of Mo feedstock. Consequently, once Mo feedstock becomes too scarce compared to Se, the edge termination of the subsequently grown $MoSe_2$ is Se; (**iii**) When the growth of $MoSe_2$ occurs in a Se-rich environment, the final sample with Se termination has a hexagonal shape. The red and black lines in edge refer to the Mo and Se terminations, respectively. **(B and C)** Edge termination of 740 °C-grown (**B**) and 800 °C-grown samples (**C**).

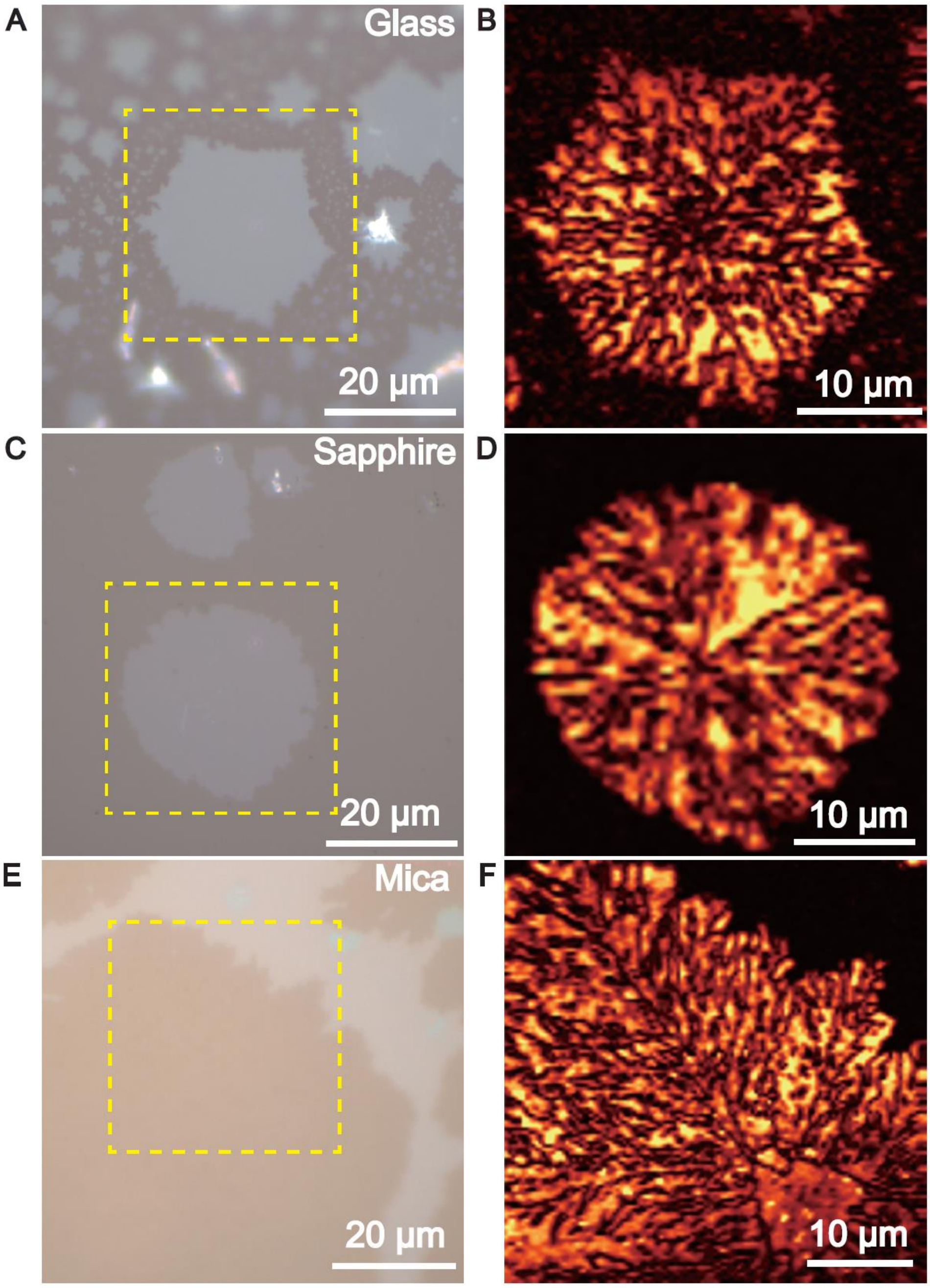

Figure S17 TB-rich $MoSe_2$ grown on arbitrary substrates. (A and B) Optical and SHG images of $MoSe_2$ grown on glass. $MoSe_2$ hexagon contains high density TBs. **(C and D)** Optical and SHG images of $MoSe_2$ grown on sapphire. $MoSe_2$ circle contains high density TBs. **(E and F)** Optical and SHG images of $MoSe_2$ grown on mica. Large-size $MoSe_2$ domain contains high density TBs.

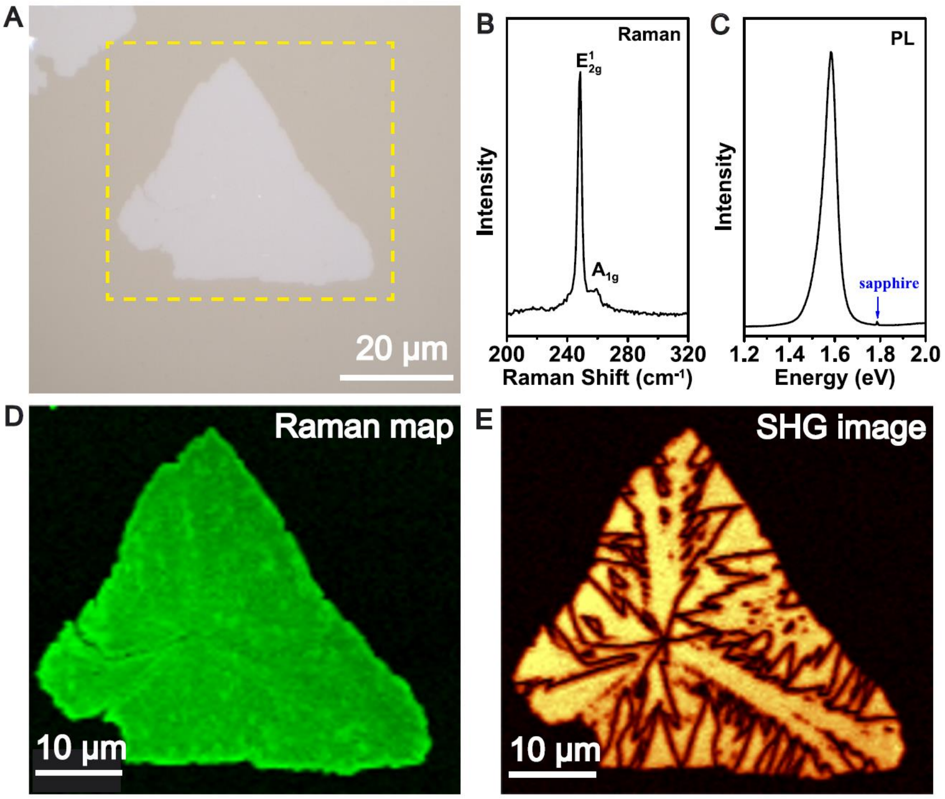

Figure S18 Morphology and spectroscopy of TB-rich WSe_2. (A) Optical image of WSe_2 grown by OH-assisted CVD method. **(B)** Raman spectrum of WSe_2 monolayers. The obvious typical peak at 240 cm^{-1} belongs to the in-plane E^1_{2g} mode of natural WSe_2. **(C)** PL spectrum of WSe_2 monolayers confirms its high crystallinity. **(D)** Raman intensity mapping of the E^1_{2g} peak. The uniform intensity demonstrates the high degree of uniform spectroscopic quality of as-grown WSe_2 flake. **(E)** SHG image of triangular WSe_2 flake. A high-density of TB in WSe_2 is similar to the as-grown $MoSe_2$.

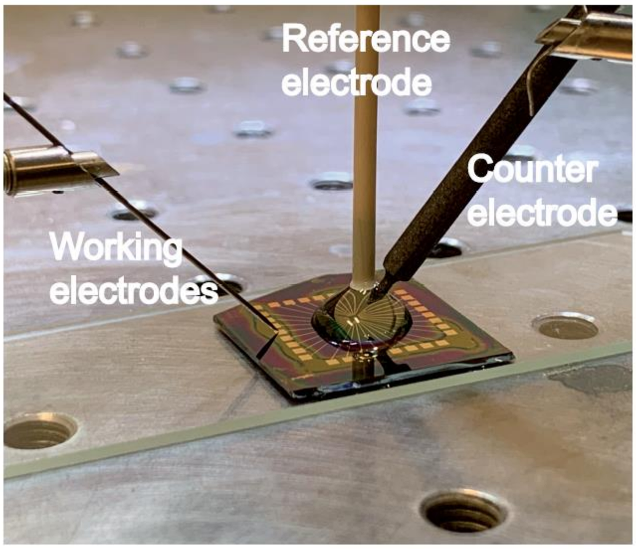

Figure S19 Photograph of a micro-electrochemical cell.

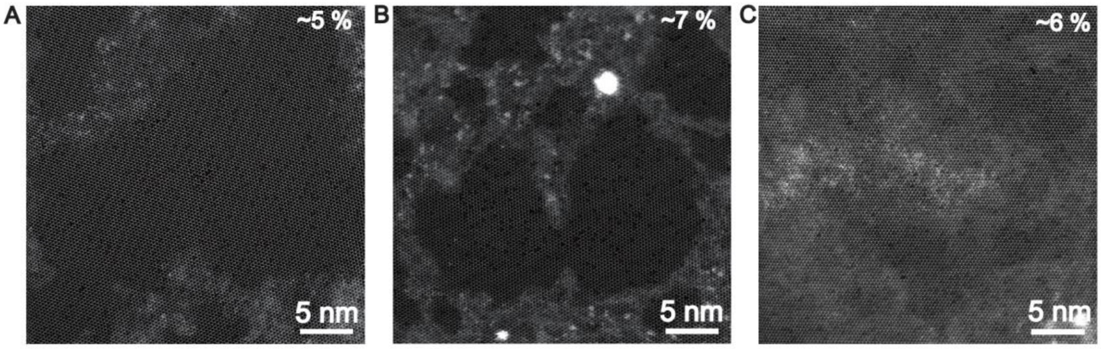

Figure S20 Atomic structure of the resultant $MoSe_2$ monolayers basal planes. High resolution STEM images of (**A**) 740 °C, (B) 800 °C and (**C**) David star shaped CVD-grown $MoSe_2$. The Se vacancy concentrations of 740 °C, 800 °C and CVD-grown $MoSe_2$ are 5%, 7% and 6%, respectively. The Se vacancy concentration in these samples are qualitatively similar, suggesting that the major catalytic contributors are TBs with 8-membered rings, while the contributions from Se vacancies and the pristine $MoSe_2$ basal plane are negligible.

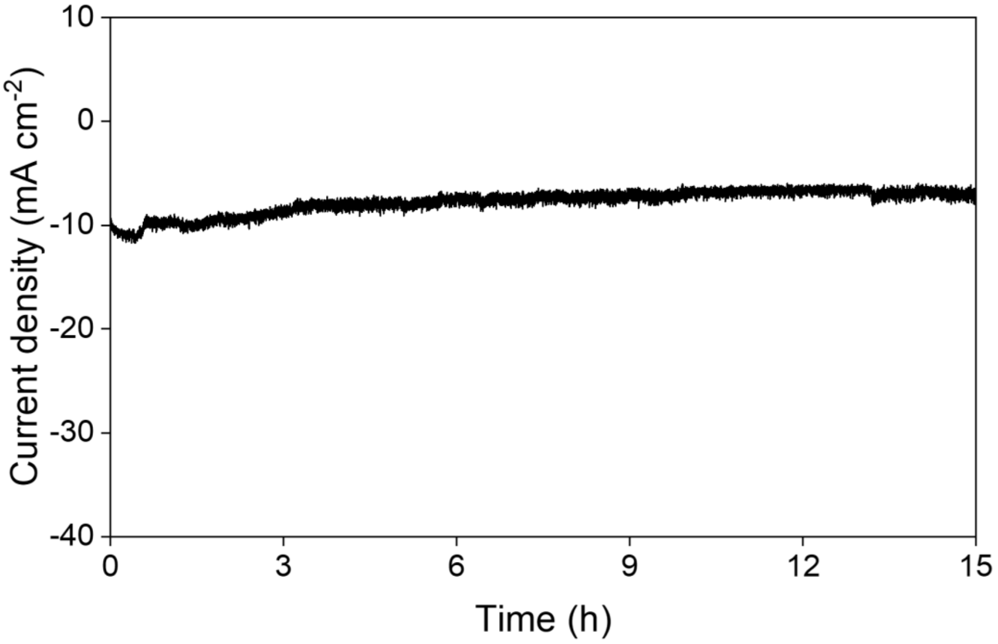

Figure S21 Electrochemical stability test of 740 °C-grown-$MoSe_2$ monolayers.

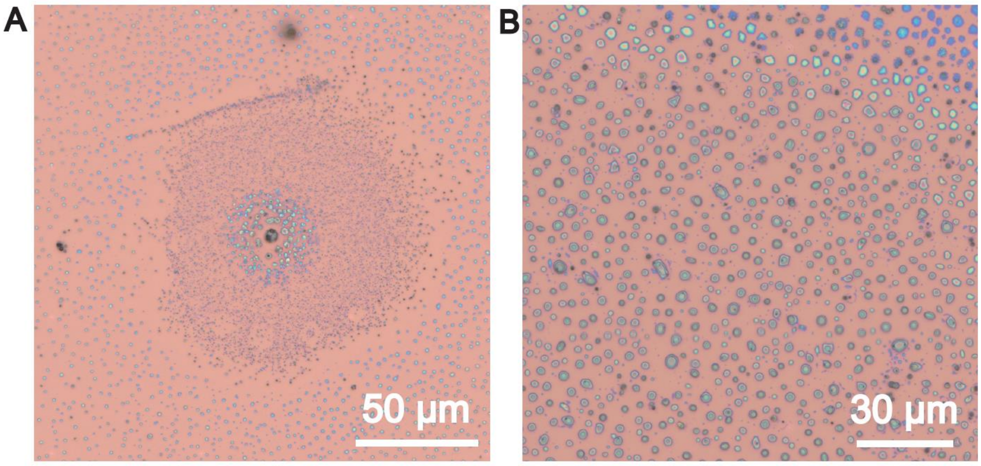

Figure S22 Morphology of intermediate products grown by CVD method at 650 °C. (A and B) Optical images of different regions. The abundant MoO_x particles precipitated onto the substrate surface.

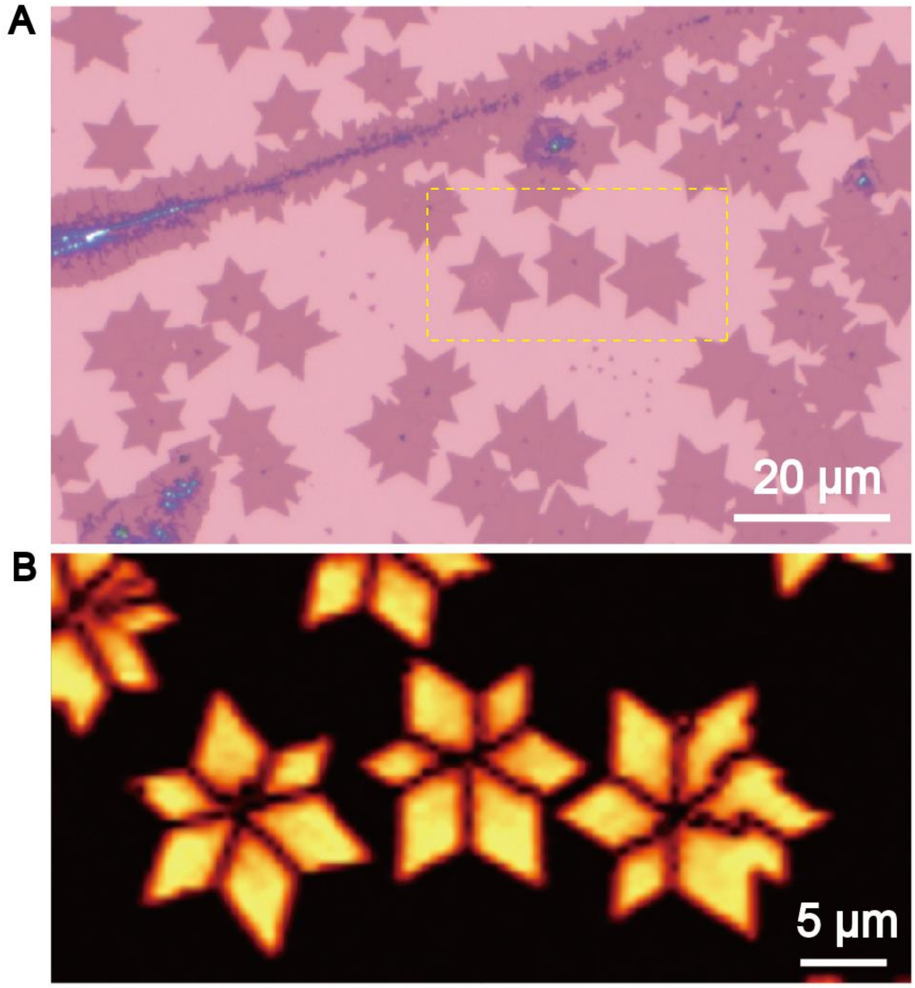

Figure S23 Morphology and spectroscopy of $MoSe_2$ grown by CVD method without precipitation stage. **(A)** Optical image of typical David star shaped $MoSe_2$. **(B)** Corresponding SHG image of these David stars in **A** shows that they only have six TBs.

T		D (m^2/s)
1000K	$MoSe_2$	6.42504E-08
	SiO_2 (glass)	3.96233E-18
	Sapphire	1.75183E-26

Table S1 Diffusion coefficient of Mo_3O_9 on the surface of $MoSe_2$, SiO_2 (glass) and sapphire at 1000 K. Due to the immense difference in diffusivities of metal feedstocks on various growth substrates and the as-formed TMD surface, high-density of TBs can be formed by the self-oriented nucleation and growth strategy.

MoS_2 or $MoSe_2$ Monolayers	η10	Tafel	References
GB-rich MoS_2 (random 4, 5, 7, 8-membered rings)	-325	95	[3]
GB-rich MoS_2 (1-3 layers)	-250	110	[4]
Segmented MoS_2 grain boundaries	-240	75	[5]
MoS_2 GB nano-channel	-266	90	[6]
Janus SMoSe	-250		[7]
TB-rich $MoSe_2$ (1D 4-membered rings)	-620	115	[8]
TB-rich $MoTe_2$ (1D 4-membered rings)	-520	72	[8]
**TB-rich $MoSe_2$ (high-density 8-membered rings)**	**-195**	**70**	**This work**

Table S2 HER performances of other grain boundary engineered TMDs.

Supplementary Notes

Supplementary Note 1

Calculation of the length of the TB per unit area. To get the total boundary length, we used Photoshop software to cut out the darker part of the flake, and used the Laplacian second-order zero-cross operator in MATLAB software to detect its edges, resulting in a domain boundary outline totaling 16560 pixels (**Figure S7d**). Considering that the 2-micron ruler occupies 380 pixels, we can calculate that the total boundary length is about 87.2 microns. The entire area of this flake is about 17.1 μm^2, so the length of the TB per unit area in our $MoSe_2$ is about 5.1 μm/μm^2. We also used the same method to calculate the TB length in typical MoS_2 David star grown on SiO_2/Si substrate[9] the highly-oriented MoS_2 film grown on the sapphire substrate[10], the results are 0.2 μm/μm^2 and 0.8 μm/μm^2, respectively.

Supplementary Note 2

Effect of temperature on the growth rate of $MoSe_2$, the diffusion rate of feedstocks and the kinetic constant (*k*). With the increase of growth temperature from T_1 to T_2, the growth rate of $MoSe_2$ increases by a factor of $\exp(-\Delta E_{\text{growth}}/k_B T_2)/\exp(-\Delta E_{\text{growth}}/k_B T_1) = \exp(\Delta E_{\text{growth}}/k_B * (1/T_1 - 1/T_2))$. Likewise, the diffusion rate of feedstocks increases by a factor of $\exp(\Delta E_{\text{diffuse}}/k_B * (1/T_1 - 1/T_2))$. Since ΔE_{growth} is much larger than $\Delta E_{\text{diffuse}}$, the growth rate should be significantly more sensitive to the temperature than the diffusion rate of feedstocks. Therefore, we focus on the effect of T on growth rates.

The influence of temperature on *k* can be inferred from Arrhenius equation[11], which formulates *k* as,

$$k = k^0 \exp(-E_a/k_B T)$$

where T is the local temperature, k_B is the Boltzmann constant, E_a is the activation energy, k^0 the prefactor.

According to

$$\dot{l_{ij}} = k(\xi_{ij} - \xi_{eq}),$$

we mentioned in **Methods**, the growth rate $(\dot{l_{ij}})$ can be represented by k, which is related to the growth temperature and follows an Arrhenius type behavior. We then provide a qualitative trend of k of 0.001, 0.01 and 0.1 at 660, 670 and 680 ℃. To obtain large-size simulation at 740 ℃, we used an extreme case to simplify the model, where the k can be considered as 1.

Supplementary Note 3

Formation process of $MoSe_2$ in a Star-of-David shape. To better understand the formation process of a fuzzy Star-of-David shape, we consider an extreme case where isolated islands cannot grow at all and suppose the Mo feedstock is excessive so that it distributes evenly on the continent surface. Indeed, the growth of $MoSe_2$ single crystals exhibits anisotropic behavior, with distinct growth rates along different directions. The ratio of the growth rate along the three tip directions to the three edge directions is 2:1 (**Figure S15**). In the case of a continent consisting of multiple grains with two opposite orientations (violet and yellow in **Figure S14C**), the radial growth rate of the continent away from its center is largest along six tip directions (red lines in **Figure S14C**) of either orientation (denoted as v_l), and smaller along bisectors of tip directions (denoted as v_s) (blue lines in **Figure S14C**), and the ratio is v_l/v_s=√3:1. As a result of these different growth rates, even though the precursor distribution is initially isotropic, the variation in growth rate among different directions can contribute to the formation of a fuzzy Star-of-David shape in the final samples.

Supplementary Note 4

Morphology evolution of $MoSe_2$ flakes from 740 °C to 800 °C. The diffusivity difference of Mo_3O_9 on SiO_2 and $MoSe_2$ surfaces strongly influences the growth morphology from 660 °C to 740 °C. A fuzzy David star shape $MoSe_2$ is grown at 740 °C. Interestingly, this fuzzy David star shape does not keep at an even longer growth time (740 °C for 3 min). In that case, the longer growth time of the continent makes Mo feedstock supplied by the central mound is no longer over abundant even for the growth of the continent itself. Then, the continent "coastline" closer to the center grows faster because it can obtain relatively more feedstock. This makes the flake shape more isotropic, resulting in a 6-point star shape (**Figure 4B$_1$**). However, when increase the annealing temperature over 740 °C, the transition of growth mode from self-oriented to edge-epitaxial is likely to reduce the abundance of Mo feedstock. Then, once Mo feedstock becomes too scarce compared to Se, the edge termination would switch from Mo to Se atoms (**Figure S16**), resulting in a multi-grain hexagon flake shape at 800 °C (**Figure 4D$_1$**).

References

1. Zhu, J., Xu, H., Zou, G. et al. (2019). MoS_2–OH bilayer-mediated growth of inch-sized monolayer MoS2 on arbitrary substrates. J. Am. Chem. Soc. ***141***, 5392-5401.
2. Kresse, G. & Furthmüller, J. (1996). Efficiency of ab-initio total energy calculations for metals and semiconductors using a plane-wave basis set. Comp. Mater. Sci. ***6***, 15-50.
3. Zhu, J., Wang, Z.-C., Dai, H. et al. (2019). Boundary activated hydrogen evolution reaction on monolayer MoS_2. Nat. Commun. ***10***, 1348.
4. He, Y., Tang, P., Hu, Z. et al. (2020). Engineering grain boundaries at the 2D limit for the hydrogen evolution reaction. Nat. Commun. ***11***, 57.
5. Yu, M., Zhu, C., He, Y. et al. (2021). Polymorphism of segmented grain boundaries in two-

dimensional transition metal dichalcogenides. Nano Lett. ***21***, 6014-6021.

6. Zhu, C., Yu, M., Zhou, J. et al. (2020). Strain-driven growth of ultra-long two-dimensional nano-channels. Nat. Commun. ***11***, 772.

7. Zhang, J., Jia, S., Kholmanov, I. et al. (2017). Janus monolayer transition-metal dichalcogenides. ACS Nano ***11***, 8192-8198.

8. Kosmala, T., Coy Diaz, H., Komsa, H. P. et al. (2018). Metallic twin boundaries boost the hydrogen evolution reaction on the basal plane of molybdenum selenotellurides. Adv. Energy Mater. ***8***, 1800031.

9. van der Zande, A. M., Huang, P. Y., Chenet, D. A. et al. (2013). Grains and grain boundaries in highly crystalline monolayer molybdenum disulphide. Nat. Mater. ***12***, 554-561.

10. Yu, H., Liao, M., Zhao, W. et al. (2017). Wafer-scale growth and transfer of highly-oriented monolayer MoS_2 continuous films. ACS Nano ***11***, 12001-12007.

11. Tabatabaei, F., Boussinot, G., Spatschek, R. et al. (2017). Phase field modeling of rapid crystallization in the phase-change material AIST. J. Appl. Phys. ***122***, 045108.
